# Comparative safety and effectiveness of apixaban and rivaroxaban for treatment of cancer-associated venous thromboembolism: A retrospective cohort study

**DOI:** 10.1371/journal.pmed.1004754

**Published:** 2025-09-26

**Authors:** Jingjing Sun, Hemalkumar B. Mehta, Jodi B. Segal, G. Caleb Alexander

**Affiliations:** 1 Center for Drug Safety and Effectiveness, Johns Hopkins Bloomberg School of Public Health, Baltimore, Maryland, United States of America; 2 Department of Epidemiology, Johns Hopkins Bloomberg School of Public Health, Baltimore, Maryland, United States of America; 3 Division of General Internal Medicine, Johns Hopkins Medicine, Baltimore, Maryland, United States of America; Leiden University Medical Center, NETHERLANDS, KINGDOM OF THE

## Abstract

**Background:**

While apixaban has demonstrated advantages over alternative direct oral anticoagulants (DOACs) in some settings, its comparative safety and effectiveness in cancer-associated venous thromboembolism (VTE) remain uncertain. Current guidelines recommend DOACs as first-line treatment for cancer-associated VTE, though they do not recommend any specific DOAC over another. This study aimed to quantify the risk of recurrent VTE, major bleeding, and clinically relevant non-major bleeding among individuals with cancer-associated VTE treated with apixaban versus rivaroxaban.

**Methods and findings:**

In this retrospective cohort study, we used data from Medicare fee-for-service (2016–2020) and MarketScan (2016–2022), two U.S. administrative claims databases covering publicly and commercially insured individuals. We included individuals aged ≥65 years (Medicare) or 18–64 years (MarketScan) with active cancer, defined as a cancer diagnosis within 6 months before an index VTE event, who newly initiated apixaban or rivaroxaban within 30 days of that event. The outcomes were (1) hospitalization for recurrent VTE; (2) hospitalization for major bleeding; and (3) hospitalization or outpatient visit for clinically relevant non-major bleeding events. Eligible individuals were followed for outcomes at 6 months (consistent with guideline recommendations) and during the entire follow-up period. We used inverse probability of treatment weighting to adjust for baseline differences, including demographics, comorbidities (e.g., prior bleed), VTE risk factors, cancer type and treatments, and medication use, and applied inverse probability of censoring weighting to account for differential loss to follow-up. We analyzed outcomes using adjusted Cox proportional hazards models, pooling estimates using an inverse variance-weighted fixed-effects model. The final cohort included 6,329 apixaban and 4,260 rivaroxaban users across both databases. At 6 months, apixaban was associated with similar risks of recurrent VTE (hazard ratio [HR] 0.66, 95% confidence interval [CI] [0.40, 1.11]; *p*-value = 0.11) and major bleeding (HR 0.95, 95% CI [0.73, 1.23]; *p* = 0.70), and a lower risk of clinically relevant non-major bleeding (HR 0.84, 95% CI [0.74, 0.96]; *p* = 0.009) compared to rivaroxaban. The same pattern persisted during the extended follow‑up. The main limitation is the observational design, which may leave residual confounding despite adjustments using inverse probability weighting.

**Conclusions:**

In cancer-associated VTE, apixaban was associated with similar risks of recurrent VTE and major bleeding, and a lower risk of clinically relevant non-major bleeding compared with rivaroxaban. These findings suggest apixaban may be a favorable option for anticoagulation in cancer-associated VTE when minimizing bleeding risk is a priority.

## Author summary

**Why was this study done?:**

- Individuals with cancer-associated venous thromboembolism (VTE) face risks of both recurrent clots and bleeding, but direct head-to-head evidence comparing apixaban and rivaroxaban in this population is lacking.
- Among individuals with cancer-associated VTE, this study examined how apixaban and rivaroxaban compare in terms of bleeding risk and prevention of recurrent VTE.

**Where did the researchers do and find?:**

- This retrospective cohort study included 6,329 apixaban and 4,260 rivaroxaban users from the U.S. Medicare and MarketScan claims data.
- The study found that apixaban was associated with similar risks of recurrent VTE and major bleeding, and a lower risk of clinically relevant non-major bleeding compared to rivaroxaban at six months; the same pattern persisted throughout extended follow‑up.

**What do these findings mean?:**

- Apixaban may offer similar effectiveness in preventing recurrent VTE and major bleeding, and a more favorable safety profile in terms of clinically relevant non-major bleeding compared to rivaroxaban among individuals with cancer-associated VTE.
- These findings may help guide treatment choices, but the results should be interpreted cautiously since this was not a randomized trial, and some important factors may not have been considered.

## Abstract 3

Jingjing Sun and colleagues compare the risk of recurrent venous thromboembolism, major bleeding, and clinically relevant non-major bleeding among individuals with cancer-associated venous thromboembolism treated with apixaban versus rivaroxaban.

## Introduction

Cancer-associated venous thromboembolism (VTE), which includes pulmonary embolism and deep vein thrombosis, accounts for approximately 20% of all VTE cases and is a leading cause of morbidity and mortality among individuals with cancer [1,2]. Compared to individuals without cancer who have VTE, those with cancer face significantly higher risks of both recurrent VTE as well as hemorrhagic complications from pharmacotherapy for VTE prevention and treatment [3,4].

Current guidelines, including those from the American Society of Clinical Oncology (ASCO) and the National Comprehensive Cancer Network (NCCN), generally recommend direct oral anticoagulants (DOACs) (i.e., apixaban, edoxaban, and rivaroxaban) or low molecular weight heparin as first-line treatment for cancer-associated VTE [5–9]. All guidelines recommend at least 3–6 months of anticoagulation, with extended treatment for active cancer or persistent risk; both the European Society of Cardiology (ESC) and NCCN specifically recommend indefinite therapy while cancer remains active [6,7].

Given DOAC’s prominent role in preventing and treating cancer-associated VTE, there is continued interest in the effectiveness and safety of apixaban and rivaroxaban, which account for nearly two-thirds of DOAC use among these individuals [10]. Three network meta-analyses of trials, three single-center studies, and a multicenter registry in Japan found a similar risk of major bleeding and recurrent VTE between apixaban and rivaroxaban in individuals with cancer-associated VTE [11–17]. These findings are limited by low event rates and small sample sizes. Two single-center studies found a significantly lower risk of clinically relevant non-major bleeding with apixaban compared to rivaroxaban [15,16], whereas two network meta-analyses found no significant difference in clinically relevant non-major bleeding risk [11,13]. An additional retrospective study reported no difference in bleeding rates but studied only lower-risk individuals with cancer, which may limit generalizability [18]. Moreover, no clinical trials have compared apixaban and rivaroxaban head-to-head for individuals with cancer-associated VTE [5,8], and real-world comparative evidence remains limited.

We quantified the safety and effectiveness of apixaban versus rivaroxaban among individuals with cancer-associated VTE. We used both Medicare claims and commercial claims and focused on clinically relevant endpoints, including recurrent VTE and major bleeding.

## Methods

### Data source and study design

We performed a retrospective, new-user cohort study using two large databases. First, we used the Merative MarketScan Research Database (January 2016 to December 2022), a large U.S. claims database containing de-identified data from employer-sponsored and Medicare Supplemental insurance plans, which includes individuals aged 18–64 years. Second, we used a 20% random sample of Medicare fee-for-service beneficiaries (January 2016 to December 2020), which includes claims for older adults (age ≥ 65 years) and individuals with disabilities in the U.S. Our reporting adheres to the Reporting of Studies Conducted using Observational Routinely-Collected Data for Pharmacoepidemiology (RECORD-PE) reporting guidelines (S1 Checklist RECORD-PE Checklist) [19].

### Study population and drug exposure

We identified adults with active cancer who newly initiated apixaban or rivaroxaban within 30 days of an index VTE event. We identified index VTE events using inpatient or outpatient ICD-10-CM codes for pulmonary embolism or deep vein thrombosis in the primary or secondary positions, which has a positive predictive value (PPV) of 91% in claims-based studies [20–23]. We defined active cancer as either two or more medical claims for a cancer diagnosis (excluding nonmelanoma skin cancer) or one cancer diagnosis claim combined with at least one claim for cancer treatment, including chemotherapy, radiation, immunotherapy, or surgery, within the 6 months prior to index VTE event [21,24,25] (Fig A in S1 File).

The index date was the first apixaban or rivaroxaban prescription filled within 30 days of the index VTE event. We included individuals if they had at least 365 days of continuous medical and pharmacy coverage before the index date. We excluded those with a prior VTE diagnosis, other indications for anticoagulation such as atrial fibrillation or a mechanical heart valve, evidence of inferior vena cava filter placement, pregnancy, or use of oral or parenteral anticoagulants in the 365 days before the index date. We also excluded individuals who used multiple anticoagulants on the index date and performed a sensitivity analysis, including those with concurrent low molecular weight heparin use at the time of the event.

### Outcomes

The primary outcomes were: (1) recurrent VTE leading to hospitalization, (2) major bleeding leading to hospitalization, and (3) clinically relevant non‑major bleeding leading to either hospitalization or an outpatient visit. We identified recurrent VTE and major bleeding using ICD-10-CM primary diagnosis codes or corresponding procedure codes in the first-listed position on inpatient claims [21,22,25]. The PPV for recurrent VTE varies widely based on study setting and approach [25–27], while PPV for major bleeding is much higher (90%–94%) [28]. To mitigate misclassification, we did not classify VTE hospitalizations occurring within 30 days of the index event as recurrent VTE.

The definition of major bleeding was informed by the International Society on Thrombosis and Haemostasis (ISTH) criteria and was operationalized as bleeding at a critical site or organ (gastrointestinal, intracranial, and other major sites requiring hospitalization), using first-listed inpatient ICD-10-CM diagnosis codes. We categorized major bleeding events into three groups: gastrointestinal (GI) bleeding, intracranial hemorrhage (ICH), and other critical-site bleeding (genitourinary, respiratory tract, ocular, and joint bleeding/hemarthrosis, as well as events requiring transfusion of blood products and bleeding events without specified locations) [25]. Clinically relevant non-major bleeding was defined as non-critical site bleeding that did not meet major bleeding criteria but required hospitalization (with bleeding recorded as a secondary diagnosis, excluding admissions where major bleeding was the primary diagnosis) or an outpatient/emergency department visit without an ICH diagnosis [25]. In analyses of clinically relevant non‑major bleeding (which was further classified as gastrointestinal or other sites), any events occurring after a major bleed were excluded [25].

### Follow-up, censoring, and treatment patterns

We adopted a per-protocol approach and followed individuals from the index date, i.e., the initiation of apixaban or rivaroxaban, until the first occurrence of any of the following: study outcome, disenrollment from medical or pharmacy insurance, the end of data availability (December 31, 2022, for MarketScan and December 31, 2020, for Medicare) or deviation from the “assigned” treatment. This was defined as discontinuation of the index anticoagulant (gap of more than 30 days between fills), switching to a non-index anticoagulant, occurrence of pregnancy, or placement of an inferior vena cava filter. Each study outcome was analyzed independently, with individuals censored at the time of the first occurrence of the respective outcome. We conducted analyses using two follow-up periods: a 6‑month window, reflecting typical treatment duration, and an unrestricted period to capture extended use. We examined treatment persistence, discontinuation, and switching. Prescription discontinuation was defined as the absence of a prescription fill for more than 30 days after the last prescription’s supply ended, with the discontinuation date set as 30 days after the last supply date [29]. Treatment switching was defined as filling a prescription for a different oral anticoagulant within 30 days before or after the end of the index prescription’s supply [30].

### Baseline covariates

We used the 1-year baseline period before the index date to define the following covariates: demographic characteristics, comorbidities, medication use, type of VTE diagnosis, VTE risk scale, bleeding history, cancer type, cancer metastasis, and cancer-related treatment. Comorbidity was measured using the Beyrer adaption of Charlson Comorbidity Index (CCI), which is validated for use with ICD-10-CM and ICD-10-PCS codes in U.S. claims data [31]. Additionally, we included other baseline comorbidities relevant to our study, such as anemia, coagulation defects, thrombophilia, recent history of falls, and fracture/trauma involving lower extremities, to provide a more comprehensive assessment of individual risk factors [25]. Medication history captured relevant drug classes, including antiarrhythmics, antiplatelets, statins, NSAIDs, hormone therapies, and others. Cancer-related therapies included chemotherapy, radiation, immunotherapy, and surgery. To address confounding by underlying thrombotic and bleeding risk, we used a claims-based version of the Khorana VTE risk score, stratifying individuals into very high, high, or other risk based on cancer type [25,32]. Bleeding risk was assessed using a modified VTE-BLEED score, which incorporates seven predictors: active cancer, male sex, age ≥60 years, prior bleeding, renal dysfunction, anemia, and antiplatelet use [33,34].

### Statistical analysis

We applied inverse probability of treatment weighting (IPTW) to balance baseline characteristics between treatment groups and minimize confounding [35]. For each database separately, we estimated propensity scores using logistic regression based on pretreatment covariates, then calculated IPTW weights [36–38]. Covariate balance was evaluated using absolute standardized mean difference, with values <0.1 indicates good balance [39]. To account for potential informative censoring due to loss to follow-up, we applied inverse probability of censoring weights (IPCW) which was calculated using logistic regression model [40]. The final analytic weight for each individual was the product of IPTW and IPCW [41].

In the weighted population, we calculated incidence rates of recurrent VTE, major bleeding, and clinically relevant non-major bleeding. We estimated hazard ratios (HRs) using weighted Cox proportional hazards models, representing the average treatment effect among the treated. Database-specific estimates were pooled using an inverse variance-weighted fixed-effects meta-analysis [42]. Analyses were conducted over two follow-up periods: a 6-month window and the entire available observation period. Sensitivity analyses included an intention-to-treat approach in which individuals were not censored at treatment discontinuation or switching. In the Medicare cohort, we also applied Fine and Gray competing risk regression to account for death as a competing event [43].

All analyses were performed using SAS version 9.4 (SAS Institute, Cary, NC) between November 2024 and April 2025. The analyses were planned a priori, and the study was approved by the Johns Hopkins Bloomberg School of Public Health Institutional Review Board (IRB# IRB00025718). The study used administrative claims data from MarketScan and Medicare. MarketScan data are fully de-identified and were accessed under license; IRB oversight and informed consent were not required. Medicare data were accessed under a Data Use Agreement with the Centers for Medicare & Medicaid Services and analyzed under IRB oversight, with informed consent waived.

## Results

### Characteristics of study population

**Fig 1:**
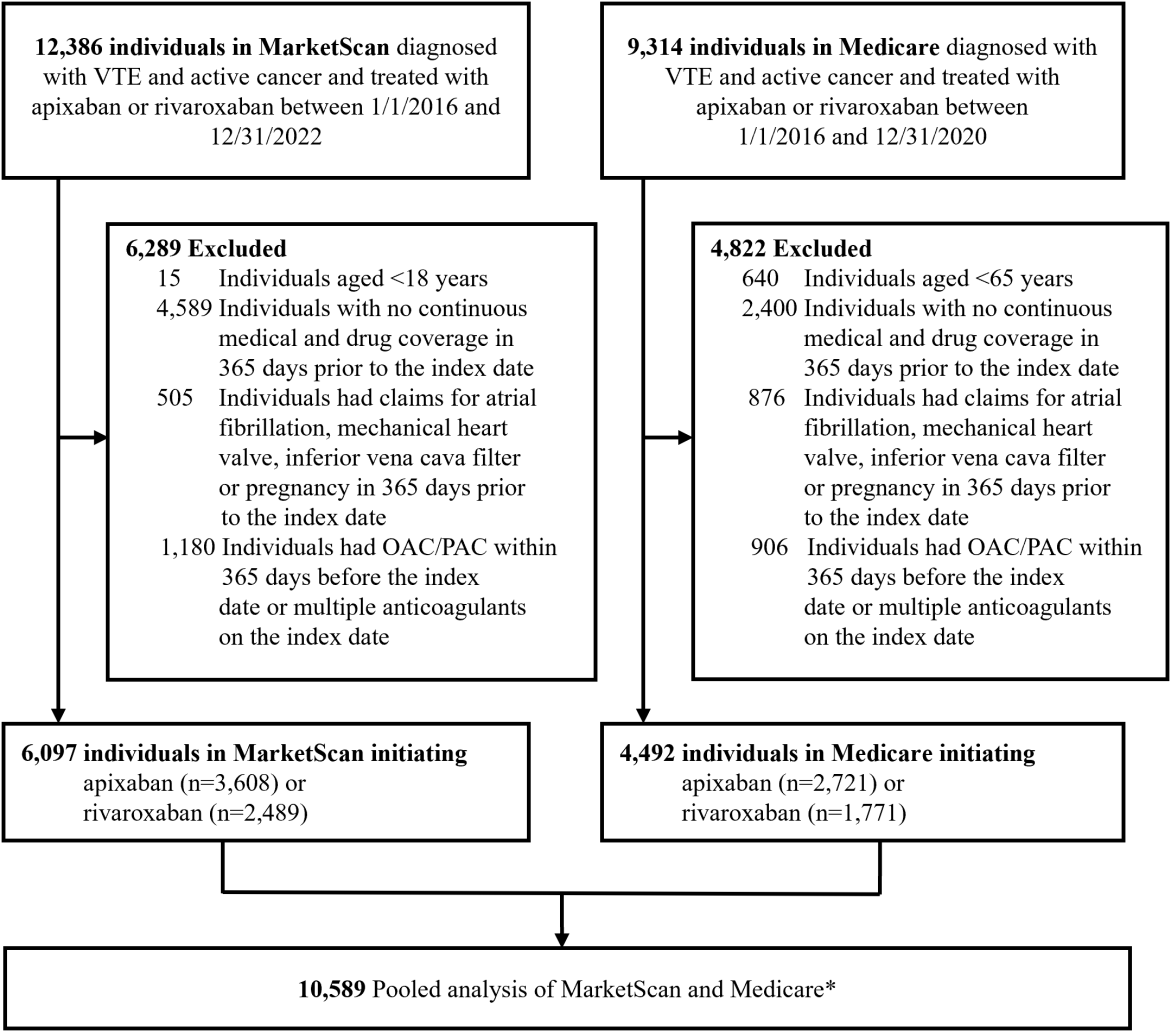
Study population flow diagram. *Pooled database-specific estimates using an inverse variance-weighted fixed effects model; OAC, oral anticoagulation; PAC, parenteral anticoagulation; VTE, venous thromboembolism.

We identified 10,589 individuals with cancer-associated VTE who initiated apixaban or rivaroxaban within 30 days following their index VTE event: 6,097 from MarketScan and 4,492 from Medicare. Across the two databases, 6,329 (59.8%) individuals were prescribed apixaban and 4,260 (40.2%) rivaroxaban (Fig 1). Commercially insured individuals were generally younger with fewer comorbidities, while Medicare beneficiaries were older with a higher burden of comorbidities and elevated VTE-related bleeding risk.

Before applying weights, apixaban users were slightly older, had a higher VTE bleed score, and had a greater burden of comorbidities compared to rivaroxaban users. Apixaban users also had higher rates of pulmonary embolism as their index VTE event and were more likely to receive their diagnosis in the inpatient setting compared to rivaroxaban users. After applying IPTW and IPCW, all baseline covariates were well-balanced between the treatment groups in both the MarketScan and Medicare cohorts (Table 1).

**Table 1:** Baseline demographics and characteristics of study cohort before and after IPTW and IPCW weighting.

	MarketScan database						Medicare database					
	Before weighting			After weighting^**^			Before weighting			After weighting^**^		
	Apixaban (*N* = 3,608)	Rivaroxaban (*N* = 2,489)	ASMD^※^	Apixaban (*N* = 3,758)	Rivaroxaban (*N* = 3,711)	ASMD^※^	Apixaban (*N* = 2,721)	Rivaroxaban (*N* = 1,771)	ASMD^※^	Apixaban (*N* = 2,921)	Rivaroxaban (*N* = 1,894)	ASMD^※^
	*n* (%) mean SD	*n* (%) mean SD		*n* (%) mean SD	*n *(%) mean SD		*n* (%) mean SD	*n *(%) mean SD		*n* (%) mean SD	*n* (%) mean SD	
Age years^*^	54.9 ± 8.5	54.1 ± 8.6	0.09	55.5 ± 8.3	55.3 ± 9.6	0.03	75.8 ± 6.9	74.9 ± 6.4	0.14	75.3 ± 6.9	75.3 ± 6.7	0.01
**Age Group**												
18–54	1,359 (37.7)	1,060 (42.6)	0.10	1,244 (33.1)	1,312 (35.3)	0.05	N/A	N/A	N/A	N/A	N/A	N/A
55–64	2,223 (61.6)	1,418 (57.0)	0.10	2,451 (65.2)	2,370 (63.9)	0.03	N/A	N/A	N/A	N/A	N/A	N/A
65–74	26 (0.7)	11 (0.4)	0.04	63 (1.7)	30 (0.8)	0.08	1,322 (48.6)	958 (54.1)	0.11	1,508 (51.6)	977 (51.6)	0.00
75–79	N/A	N/A	N/A	N/A	N/A	N/A	642 (23.6)	418 (23.6)	0.00	668 (22.9)	458 (24.2)	0.03
80+	N/A	N/A	N/A	N/A	N/A	N/A	757 (27.8)	395 (22.3)	0.13	745 (25.5)	459 (24.2)	0.03
**Sex** ^*^												
Female	2,020 (56.0)	1,323 (53.2)	0.06	2,069 (55.1)	2,046 (55.1)	0.00	1,499 (55.1)	1,004 (56.7)	0.03	1,684 (57.6)	1,071 (56.5)	0.02
**Geographic Region** ^*^												
Northeast	431 (11.9)	430 (17.3)	0.15	453 (12.1)	460 (12.4)	0.01	457 (16.8)	355 (20.0)	0.08	517 (17.7)	355 (18.7)	0.03
South	856 (23.7)	545 (21.9)	0.04	916 (24.4)	900 (24.2)	0.00	1,101 (40.5)	620 (35.0)	0.11	1,135 (38.9)	708 (37.4)	0.03
Midwest	1,843 (51.1)	1,149 (46.2)	0.10	1,902 (50.6)	1,876 (50.6)	0.00	550 (20.2)	347 (19.6)	0.02	573 (19.6)	381 (20.1)	0.01
West	474 (13.1)	363 (14.6)	0.04	482 (12.8)	473 (12.7)	0.00	476 (17.5)	365 (20.6)	0.08	544 (18.6)	363 (19.1)	0.01
Other	4 (0.1)	2 (0.1)	0.01	5 (0.1)	3 (0.1)	0.01	137 (5.0)	84 (4.7)	0.01	152 (5.2)	88 (4.7)	0.03
**Setting of index VTE event** ^*^												
Outpatient	2,982 (82.6)	2,237 (89.9)	0.21	3,114 (82.9)	3,120 (84.1)	0.03	1,827 (67.1)	1,381 (78.0)	0.25	2,010 (68.8)	1,328 (70.1)	0.03
Inpatient	626 (17.4)	252 (10.1)	0.21	644 (17.1)	591 (15.9)	0.03	894 (32.9)	390 (22.0)	0.25	911 (31.2)	566 (29.9)	0.03
**VTE diagnosis** ^*^												
PE with or without DVT	1,604 (44.5)	939 (37.7)	0.14	1,698 (45.2)	1,643 (44.3)	0.02	1,207 (44.4)	706 (39.9)	0.09	1,297 (44.4)	825 (43.5)	0.02
DVT only	2,089 (57.9)	1,597 (64.2)	0.13	2,152 (57.3)	2,159 (58.2)	0.02	1,514 (55.6)	1,065 (60.1)	0.09	1,624 (55.6)	1,070 (56.5)	0.02
**Baseline Comorbidities** ^*^												
Charlson Comorbidity Index	5.8 ± 3.3	5.6 ± 3.3	0.07	6.0 ± 3.4	6.0 ± 4.1	0.00	6.4 ± 3.5	6.1 ± 3.5	0.08	6.9 ± 3.6	6.9 ± 3.7	0.00
VTE Bleed Score	3.8 ± 1.6	3.6 ± 1.5	0.12	3.9 ± 1.6	3.8 ± 1.9	0.03	5.3 ± 1.6	5.0 ± 1.4	0.22	5.2 ± 1.6	5.2 ± 1.5	0.05
Cerebrovascular Disease	208 (5.8)	140 (5.6)	0.01	232 (6.2)	244 (6.6)	0.02	233 (8.6)	125 (7.1)	0.06	238 (8.2)	167 (8.8)	0.02
Peptic Ulcer Disease	57 (1.6)	38 (1.5)	0.00	63 (1.7)	59 (1.6)	0.01	62 (2.3)	40 (2.3)	0.00	75 (2.6)	48 (2.5)	0.00
Diabetes	651 (18.0)	374 (15.0)	0.08	684 (18.2)	636 (17.1)	0.03	673 (24.7)	399 (22.5)	0.05	703 (24.1)	487 (25.7)	0.04
Hemiplegia	92 (2.5)	39 (1.6)	0.07	106 (2.8)	84 (2.3)	0.04	60 (2.2)	17 (1.0)	0.10	56 (1.9)	33 (1.7)	0.02
Renal Disease	233 (6.5)	118 (4.7)	0.08	241 (6.4)	227 (6.1)	0.01	565 (20.8)	248 (14.0)	0.18	542 (18.6)	307 (16.2)	0.06
Liver Disease	716 (19.8)	428 (17.2)	0.07	774 (20.6)	757 (20.4)	0.01	401 (14.7)	293 (16.5)	0.05	521 (17.8)	334 (17.6)	0.01
Central Venous Catheter	276 (7.6)	136 (5.5)	0.09	277 (7.4)	287 (7.7)	0.01	223 (8.2)	90 (5.1)	0.13	224 (7.7)	129 (6.8)	0.03
Coagulation Defects	164 (4.5)	86 (3.5)	0.06	169 (4.5)	156 (4.2)	0.02	318 (11.7)	194 (11.0)	0.02	360 (12.3)	225 (11.9)	0.01
Ischemic Heart Disease	126 (3.5)	51 (2.0)	0.09	136 (3.6)	124 (3.3)	0.02	690 (25.4)	377 (21.3)	0.10	707 (24.2)	468 (24.7)	0.01
Peripheral Vascular Disease	259 (7.2)	174 (7.0)	0.01	270 (7.2)	254 (6.9)	0.01	443 (16.3)	280 (15.8)	0.01	508 (17.4)	327 (17.2)	0.00
Hyperlipidemia	1,150 (31.9)	748 (30.1)	0.04	1,218 (32.4)	1,200 (32.3)	0.00	1,522 (55.9)	891 (50.3)	0.11	1,567 (53.6)	994 (52.5)	0.02
Hypertension	1,661 (46.0)	1,042 (41.9)	0.08	1,766 (47.0)	1,720 (46.3)	0.01	1,998 (73.4)	1,196 (67.5)	0.13	2,092 (71.6)	1,347 (71.1)	0.01
Obesity	973 (27.0)	600 (24.1)	0.07	998 (26.5)	976 (26.3)	0.01	510 (18.7)	285 (16.1)	0.07	540 (18.5)	341 (18.0)	0.01
Thrombophilia	144 (4.0)	93 (3.7)	0.01	153 (4.1)	144 (3.9)	0.01	107 (3.9)	64 (3.6)	0.02	117 (4.0)	78 (4.1)	0.01
COPD	423 (11.7)	209 (8.4)	0.11	492 (13.1)	442 (11.9)	0.04	681 (25.0)	343 (19.4)	0.14	714 (24.4)	447 (23.6)	0.02
Inflammatory bowel disease	64 (1.8)	32 (1.3)	0.04	66 (1.8)	74 (2.0)	0.02	27 (1.0)	29 (1.6)	0.06	39 (1.3)	30 (1.6)	0.02
Recent History of Falls	58 (1.6)	31 (1.2)	0.03	58 (1.5)	52 (1.4)	0.01	13 (0.5)	6 (0.3)	0.02	14 (0.5)	8 (0.4)	0.01
Any Bleeding	689 (19.1)	410 (16.5)	0.07	745 (19.8)	708 (19.1)	0.02	573 (21.1)	315 (17.8)	0.08	630 (21.6)	388 (20.5)	0.03
Cancer Metastasis	1,559 (43.2)	1,051 (42.2)	0.02	1,721 (45.8)	1,694 (45.6)	0.00	1,073 (39.4)	710 (40.1)	0.01	1,468 (50.2)	960 (50.7)	0.01
**Cancer type** ^*^												
Non-Hematological	3,132 (86.8)	2,136 (85.8)	0.03	3,360 (89.4)	3,316 (89.3)	0.00	2,269 (83.4)	1,499 (84.6)	0.03	383 (13.1)	251 (13.2)	0.00
Hematological	476 (13.2)	353 (14.2)	0.03	398 (10.6)	396 (10.7)	0.00	452 (16.6)	272 (15.4)	0.03	2,538 (86.9)	1,643 (86.8)	0.00
**VTE Risk Scale** ^*^												
Other Cancer	2,031 (56.3)	1,402 (56.3)	0.00	1,989 (52.9)	2,012 (54.2)	0.03	1,388 (51.0)	884 (49.9)	0.02	1,334 (45.7)	843 (44.5)	0.02
High-risk	1,156 (32.0)	806 (32.4)	0.01	1,275 (33.9)	1,231 (33.2)	0.02	1,059 (38.9)	696 (39.3)	0.01	1,158 (39.6)	769 (40.6)	0.02
Very high risk	421 (11.7)	281 (11.3)	0.01	494 (13.1)	468 (12.6)	0.02	274 (10.1)	191 (10.8)	0.02	430 (14.7)	282 (14.9)	0.01
**Cancer-related treatment** ^*^												
Any Cancer Treatment	2,381 (66.0)	1,669 (67.1)	0.02	2,554 (68.0)	2,539 (68.4)	0.01	1,283 (47.2)	882 (49.8)	0.05	1,424 (48.8)	965 (51.0)	0.04
Chemotherapy	2,095 (58.1)	1,491 (59.9)	0.04	2,236 (59.5)	2,234 (60.2)	0.01	671 (24.7)	439 (24.8)	0.00	739 (25.3)	470 (24.8)	0.01
Hormone therapy	123 (3.4)	106 (4.3)	0.04	118 (3.1)	136 (3.7)	0.03	343 (12.6)	236 (13.3)	0.02	359 (12.3)	245 (12.9)	0.02
Immunotherapy	229 (6.3)	121 (4.9)	0.07	352 (9.4)	300 (8.1)	0.05	90 (3.3)	43 (2.4)	0.05	108 (3.7)	56 (2.9)	0.04
Radiation Therapy	727 (20.1)	437 (17.6)	0.07	902 (24.0)	838 (22.6)	0.03	589 (21.6)	426 (24.1)	0.06	698 (23.9)	484 (25.6)	0.04
Cancer Surgery	380 (10.5)	263 (10.6)	0.00	367 (9.8)	349 (9.4)	0.01	216 (7.9)	141 (8.0)	0.00	226 (7.7)	150 (7.9)	0.01

** After weighing, sample sizes reflect the sum of stabilized weights and do not represent actual patient counts.

※ Absolute standardized mean difference < 0.1 indicates that the baseline characteristics between the treatment groups are well-balanced, suggesting comparability and reduced confounding.

* Variables that were adjusted to balance individual characteristics between apixaban and rivaroxaban cohorts.

**Abbreviations:** ACE Inhibitors/ARBs: Angiotensin-Converting Enzyme Inhibitors/ Angiotensin II Receptor Blockers; ASMD: Absolute standardized mean difference; COPD: Chronic obstructive pulmonary disease; DVT: Deep vein thrombosis; IPCW: Inverse probability of censoring weighting; IPTW: Inverse probability of treatment weighting; N: Number; N/A: Not available; NSAIDs: Non-Steroidal Anti-Inflammatory Drugs; PE: Pulmonary embolism; SD: standard deviation; VTE: Venous thromboembolism.

In the weighted MarketScan cohort, apixaban and rivaroxaban exhibited similar treatment patterns both at 6 months and over the entire follow-up period. Comparable patterns were observed in the Medicare cohort at 6 months (Fig B in S1 File). Over the entire follow-up period, persistence in the Medicare cohort was modestly higher for apixaban (41%) than for rivaroxaban (36%).

### Comparative safety of apixaban and rivaroxaban (bleeding)

**Fig 2:**
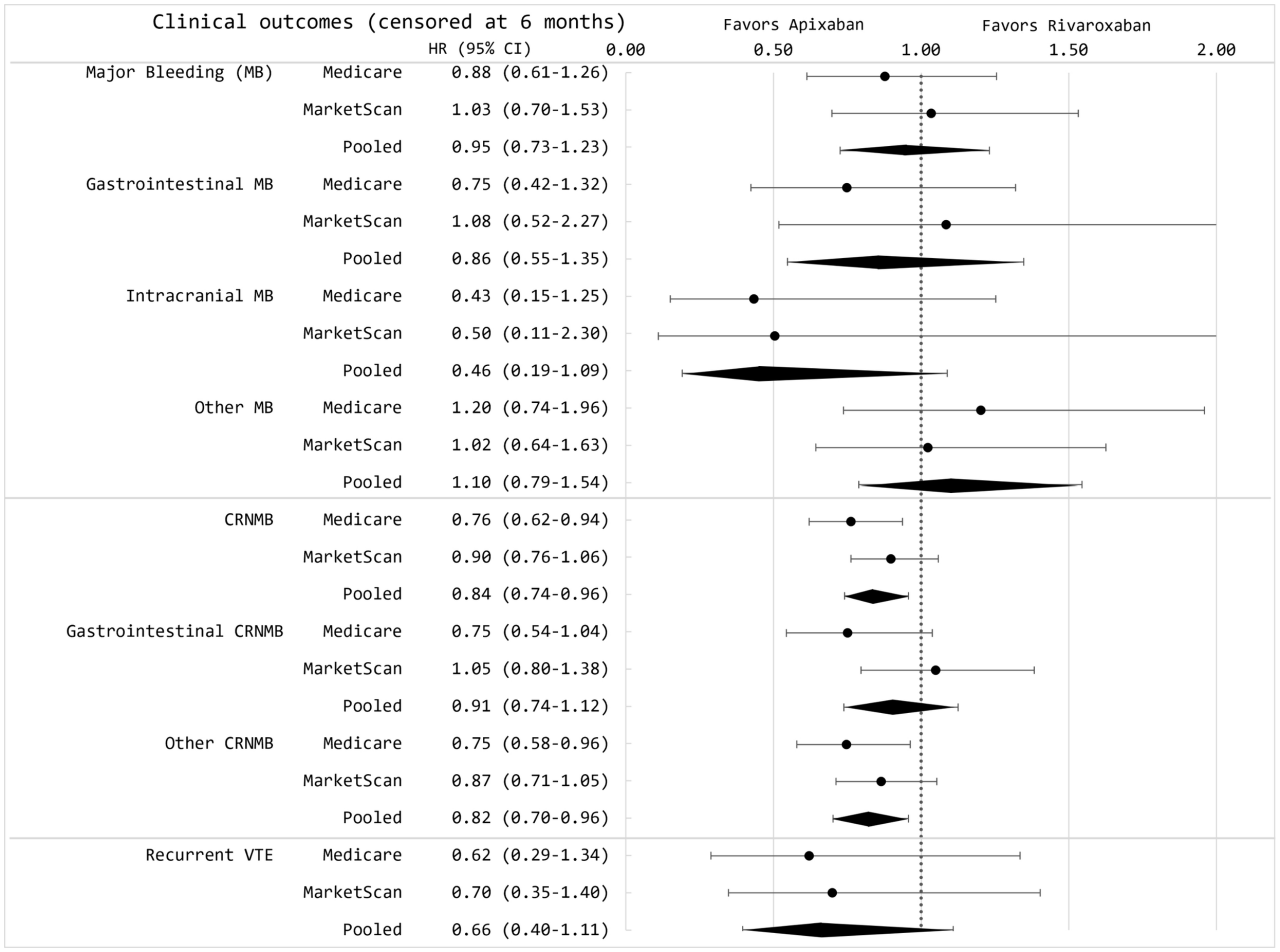
Hazard ratios of major bleeding, clinically relevant non-major bleeding, and recurrent VTE among individuals with cancer-associated VTE, censored at 6 months. CRNMB, clinically relevant non-major bleeding; GI, gastrointestinal; MB, major bleeding; VTE, venous thromboembolism.

At six months, the mean follow-up duration was similar between groups, with apixaban users averaging approximately 126 days and rivaroxaban users 129 days. The adjusted incidence rates per 100 person-years for apixaban versus rivaroxaban were 7.44 versus 7.36 for major bleeding (including GI bleeding, ICH, and other bleeding) and 30.54 versus 36.43 for clinically relevant non-major bleeding at 6 months (Table B in S1 File). Among Medicare beneficiaries, apixaban demonstrated a lower risk of clinically relevant non-major bleeding (Hazard ratio [HR]: 0.76, 95% CI [0.62, 0.94]; *p*-value = 0.01; Fine Gray sub-distribution HR 0.73, 95% CI [0.61, 0.87]; *p* < 0.001), whereas no significant difference was observed in the MarketScan cohort (Fig 2 and Table A in S1 File). In the combined analysis across both databases, apixaban was associated with a lower risk of clinically relevant non-major bleeding (HR 0.84, 95% CI [0.74, 0.96]; *p* = 0.009; *p* = 0.4 for heterogeneity), with no difference in major bleeding (HR 0.95, 95% CI [0.73, 1.23]; *p* = 0.70; *p* = 0.7 for heterogeneity) compared to rivaroxaban (Fig 2). Kaplan–Meier curves for each database are shown in Figs C and D in S1 File.

**Fig 3:**
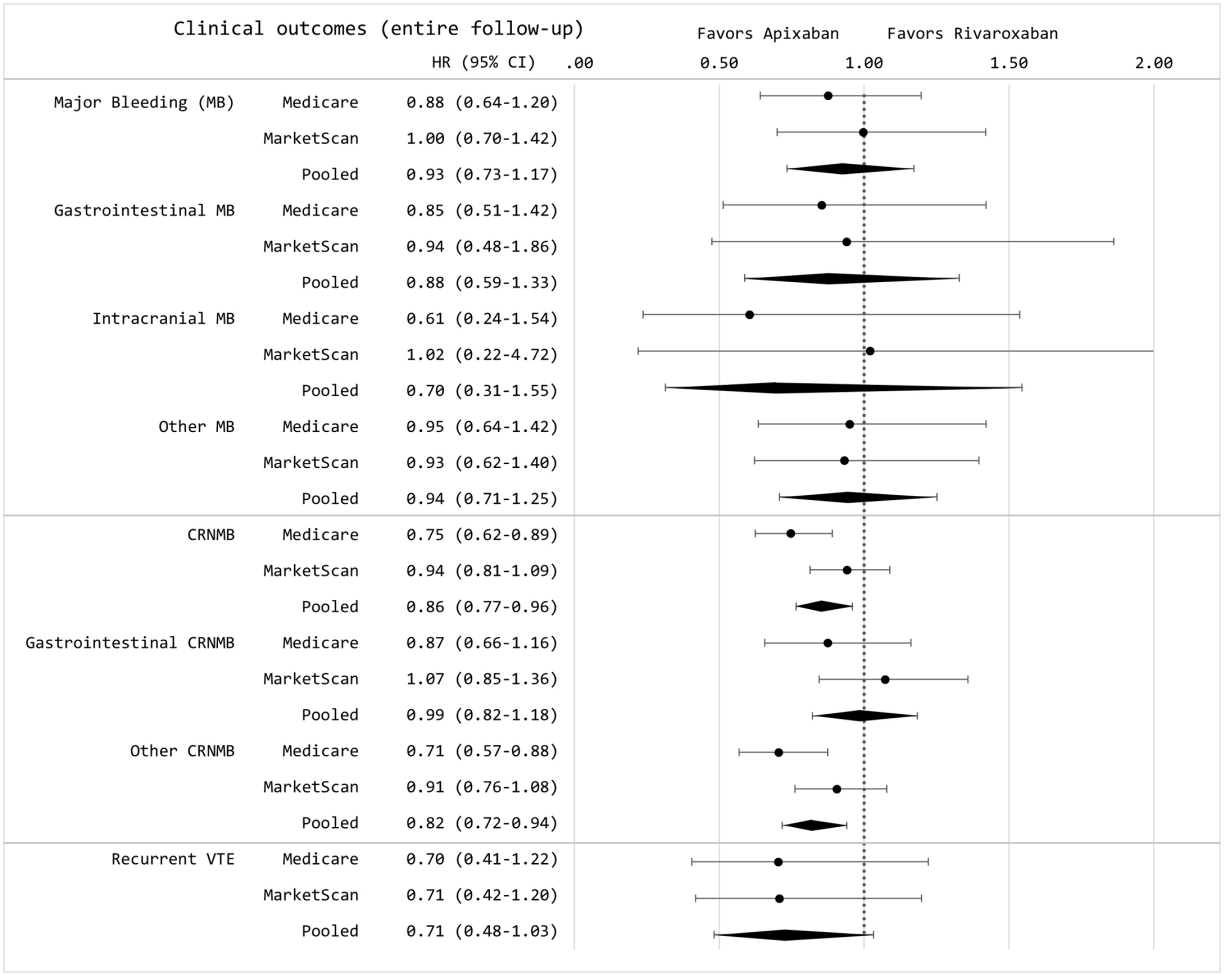
Hazard ratios of major bleeding, clinically relevant non-major bleeding, and recurrent VTE among individuals with cancer-associated VTE over the entire follow-up. CRNMB, clinically relevant non-major bleeding; GI, gastrointestinal; MB, major bleeding; VTE, venous thromboembolism.

In the extended follow-up period, averaging 289 days for apixaban and 342 days for rivaroxaban users, nearly half of patients remained on treatment. Over this duration, the adjusted incidence rates per 100 person-years for apixaban versus rivaroxaban were 4.00 versus 3.63 for major bleeding, and 17.20 versus 18.33 for clinically relevant non-major bleeding (Table B in S1 File). Apixaban remained associated with a lower risk of clinically relevant non-major bleeding (HR 0.86, 95% CI: [0.77, 0.96]; *p* = 0.007; *p* = 0.2 for heterogeneity), while the risk of major bleeding remained similar between the two DOACs (Fig 3). These results remained consistent when including or excluding individuals with short-term use of low molecular weight heparin at baseline.

### Comparative effectiveness of apixaban and rivaroxaban (recurrent VTE)

The adjusted incidence rate of recurrent VTE for apixaban versus rivaroxaban was 1.71 versus 2.50 per 100 person-years at 6 months, and 1.23 versus 1.61 per 100 person-years over the entire follow-up period (Table B in S1 File). The risk of recurrent VTE was not significantly different at either 6 months (HR 0.66, 95% CI [0.40, 1.11]; *p* = 0.11; *p* = 0.9 for heterogeneity) or over the full follow-up (HR 0.71, 95% CI [0.48, 1.03]; *p* = 0.08; *p* = 0.9 heterogeneity) (Figs 2 and 3). To assess the robustness of our findings, we conducted sensitivity analyses using alternative exclusion windows of 7 days for defining recurrent VTE (instead of the 30-day window used in the primary analysis), which yielded similar results (Tables A, C, and D from S1 File). Findings were also unchanged in an intention-to-treat analysis in which patients were not censored for treatment switching or discontinuation (Table C in S1 File).

## Discussion

Despite the high prevalence of VTE among individuals with cancer, the comparative safety and effectiveness of apixaban and rivaroxaban are unclear. In this retrospective cohort study using U.S. Medicare and MarketScan data, we found that apixaban was associated with similar risk of recurrent VTE and major bleeding, and a lower risk of clinically relevant non-major bleeding compared to rivaroxaban. These findings were consistent in sensitivity analyses that altered time exclusion window for recurrent VTE definition, varied the criteria for baseline low molecular weight heparin usage exclusion, applied an intention-to-treat analysis approach, or followed individuals for more than six months. The lower clinically relevant non-major bleeding risk with apixaban was primarily observed in the Medicare cohort, possibly reflecting differences in patient mix or healthcare use across databases, and was consistent in the pooled analysis.

Our findings extend the work of prior observational studies comparing the safety and effectiveness of apixaban and rivaroxaban in cancer-associated VTE [11–18]. While most prior studies have suggested apixaban’s superior safety profile and comparable effectiveness, our study offers several methodological advantages. For instance, we included individuals from two large U.S. databases, enhancing generalizability and offering comprehensive insights, overcoming limitations of previous studies that relied on single-center data [15,16], low-bleeding risk populations[18], indirect trial comparison [11–13], or non-U.S. studies [14,17]. We carefully controlled for potential confounders and employed several sensitivity analyses to check the robustness of the study findings. The COBRRA randomized trial recently reported the first direct comparative evidence between apixaban and rivaroxaban for acute VTE treatment [44]. Results presented at the ISTH 2025 Congress demonstrated that apixaban had comparable efficacy for preventing recurrent VTE and significantly less clinically relevant bleeding (including both major and non-major bleeding) compared to rivaroxaban [45]. The present study’s findings are broadly consistent with the COBRRA results and complement them by focusing on cancer-associated VTE, a population with distinct bleeding risks and clinical complexity. As COBRRA excluded patients with active cancer, our real-world study provides much-needed evidence in this high-risk group not addressed by this randomized trial.

Several potential mechanisms may explain our finding of greater risk of clinically relevant non-major bleeding with rivaroxaban compared to apixaban. The first mechanism relates to the drugs’ pharmacokinetics and dosing schedules, which can lead to greater fluctuations in anticoagulant effect with rivaroxaban [22]. Rivaroxaban is administered just once daily after the initial treatment period, while apixaban is dosed twice daily throughout treatment [22,46]. To maintain therapeutic efficacy with this once-daily dosing, rivaroxaban requires higher peak concentrations than apixaban [22,47,48], which may increase bleeding risk [22,49]. Additionally, rivaroxaban’s higher bioavailability and prolonged exposure in the digestive tract create higher local drug concentrations that directly damage the gastrointestinal mucosal lining [50].

Our results have implications for patients, providers and other stakeholders in maximizing quality of care for individuals at risk of cancer-associated VTE. The lower risk of clinically relevant non-major bleeding with apixaban could be especially important for patients at higher risk of treatment complications, such as those on concurrent chemotherapy or with thrombocytopenia. For individuals with cancer requiring consistent anticoagulation, the reduced clinically relevant non-major bleeding risk might help minimize treatment interruptions while maintaining protective effects against recurrent VTE. Clinically relevant non-major bleeding can also impair quality of life and cause distress for patients, families, and care teams, particularly among individuals with advanced cancer [51]. However, clinicians should continue to consider individual patient factors such as renal function, drug interactions, and patient preference when selecting between DOACs [22,52].

The clinical significance of our findings becomes apparent when considering population-level impacts. Our analysis demonstrates that treating individuals with cancer-associated VTE with apixaban versus rivaroxaban reduces clinically relevant non-major bleeding from 36.43 to 30.54 events per 100 person-years at 6 months, translating to 1 non-major bleeding event prevented for every 17 individuals treated with apixaban versus rivaroxaban for one year. In a typical oncology practice caring for 1,000 individuals with cancer-associated VTE annually, this intervention could prevent approximately 58 clinically relevant non-major bleeding episodes each year. In 2019, apixaban accounted for 40.7% of anticoagulant prescriptions in individuals with cancer-associated VTE, compared to 23.0% for rivaroxaban [10]. Even a modest additional shift toward apixaban could prevent hundreds of clinically relevant non-major bleeding events nationwide, highlighting how relative risk reductions can translate into substantial absolute benefits at the population level [53].

Our study has several limitations. First, given the observational nature of our analysis, we cannot rule out the possibility of unmeasured confounding. Although IPTW balances the measured confounders, it may not control for unmeasured confounders such as patient or clinician preferences. Second, our outcomes may be subject to misclassification—our definition of recurrent VTE included only inpatient events and has a widely ranging positive predictive value (PPV) of 26%–93% [26]. We chose this definition to maximize specificity, and this is unlikely to be differential across the two treatment groups. Some of the algorithms that we used, such as those to identify clinically relevant non-major bleeding, have not been externally validated. However, we applied conservative definitions based on diagnosis position and care setting to improve specificity, and any misclassification would likely affect both treatment groups equally. Third, although we accounted for competing risk of death in the Medicare cohort, incomplete mortality data in MarketScan limits a similar adjustment in that cohort. As above, we think this will not be differential across treatment groups. Fourth, we used outpatient prescription claims as a proxy for medication utilization. Fifth, while our use of both commercial and Medicare claims enhanced the diversity of our population, our study’s generalizability is limited by the exclusion of other populations such as those who have Medicaid, are uninsured, or lack 12 months of continuous enrollment. Sixth, analyses by cancer site were beyond our scope due to limited sample sizes and lack of granular clinical information in claims data. Finally, while this study provides valuable real-world evidence, it did not include edoxaban or dabigatran due to their low prescription rates in the United States. Researchers should explore the effectiveness of all DOACs for cancer-associated VTE, particularly in regions where edoxaban and dabigatran are more widely used. In addition, researchers should assess reduced-dose strategies for extended therapy, as a recent randomized trial supports the non-inferiority of low-dose apixaban for cancer-associated VTE [54].

In this real-world study of cancer-associated VTE, apixaban was associated with similar risks of recurrent VTE and major bleeding compared to rivaroxaban, along with a lower risk of clinically relevant non-major bleeding, suggesting a more favorable overall safety profile. The results provide guidance for clinicians managing cancer-associated VTE, especially where clinically relevant non-major bleeding is a concern.

## Supporting information

S1 File **Table A.** Fine Gray risk regression outcomes for Medicare database. *CI, confidence interval; CRNM, clinically relevant non-major; Ref, reference group; VTE, venous thromboembolism.* **Table B.** Incidence rate per 100 person-years by outcome (pooled analysis). *CRNM, clinically relevant non-major; VTE, venous thromboembolism.* **Table C.** Adjusted hazard ratios using an intention-to-treat approach (pooled analysis). *CI, confidence interval; CRNM, clinically relevant non-major; Ref, reference group; VTE, venous thromboembolism.* **Table D.** Results from additional sensitivity analyses. *Sensitivity analysis using alternative exclusion windows of seven days for defining recurrent VTE, instead of the 30-day window used in the primary analysis. **Table E.** List of inclusion and exclusion criteria codes. *CPT, Current Procedural Terminology; HCPCS, Healthcare Common Procedure Coding System; ICD-10-CM, International Classification of Diseases, 10th Revision, Clinical Modification; ICD-10-PCS, International Classification of Diseases, 10th Revision, Procedure Coding System; VTE, venous thromboembolism.* **Table F.** List of chemotherapy agents, chemotherapy administration, and radiation therapy codes. *CPT, Current Procedural Terminology; HCPCS, Healthcare Common Procedure Coding System; ICD-10-CM, International Classification of Diseases, 10th Revision, Clinical Modification; ICD-10-PCS, International Classification of Diseases, 10th Revision, Procedure Coding System; IV, intravenous; NDC, National Drug Code.* **Table G.** List of baseline covariates. *AIDS, acquired immunodeficiency syndrome; COPD, chronic obstructive pulmonary disease; CPT, Current Procedural Terminology; GI, gastrointestinal; HCPCS, Healthcare Common Procedure Coding System; ICD-10-CM, International Classification of Diseases, 10th Revision, Clinical Modification; ICD-10-PCS, International Classification of Diseases, 10th Revision, Procedure Coding System.* **Table H.** List of baseline medication codes. *NDC codes will be used wherever applicable. ACE, angiotensin-converting enzyme; ARBs, angiotensin II receptor blockers; HCPCS, Healthcare Common Procedure Coding System; NDC, National Drug Code; NSAID, nonsteroidal anti-inflammatory drug. **Table I.** List of major bleeding, clinically relevant non-major bleeding, and recurrent VTE codes. *CRNMB, clinically relevant non-major bleeding; ICD-10-CM, International Classification of Diseases, 10th Revision, Clinical Modification; ICD-10-PCS, International Classification of Diseases, 10th Revision, Procedure Coding System; MB, major bleeding; VTE, venous thromboembolism.* **Table J.** List of oral and parenteral anticoagulation codes. *Codes from the NDC system will also be used to characterize the use of PAC. HCPCS, Healthcare Common Procedure Coding System; NDC, National Drug Code; OAC, oral anticoagulant; PAC, parenteral anticoagulation. **Fig A.** Graphical depiction of study design. **Fig B.** MarketScan and Medicare treatment patterns. **Fig C.** Adjusted Kaplan–Meier curves for major bleeding by database and follow-up time. **Fig D.** Adjusted Kaplan–Meier curves for clinically relevant non-major bleeding by database and follow-up time. **Fig E.** Adjusted Kaplan–Meier curves for recurrent VTE by database and follow-up time.
(DOCX)

S1 Checklist RECORD-PE Checklist. (DOCX)

## Abbreviations

ASCO: American Society of Clinical Oncology
CI: confidence interval
CRNMB: clinically relevant non‑major bleeding
DOAC: direct oral anticoagulant
ESC: European Society of Cardiology
FDA: Food and Drug Administration
GI: gastrointestinal
HR: hazard ratio
ICD‑10‑CM: international classification of diseases, 10th revision, clinical modification
ICH: intracranial hemorrhage
IPCW: inverse probability of censoring weighting
IPTW: inverse probability of treatment weighting
IRB: institutional review board
ISTH: International Society on Thrombosis and Haemostasis
NCCN: National Comprehensive Cancer Network
PPV: positive predictive value
RECORD-PE: reporting of studies conducted using observational routinely-collected data for pharmacoepidemiology
SD: standard deviation
U.S.: United States
VTE: venous thromboembolism
VTE‑BLEED: venous thromboembolism bleeding risk score
