# Supplementary material for: Comparative safety and effectiveness of apixaban and rivaroxaban for treatment of cancer-associated venous thromboembolism: A retrospective cohort study
Source: PLoS Med. 2025 Sep 26;22(9):e1004754. doi: 10.1371/journal.pmed.1004754 (PMC12494242; doi:10.1371/journal.pmed.1004754)
Supplement: S1 File — Table A. Fine Gray risk regression outcomes for Medicare database. CI, confidence interval; CRNM, clinically relevant non-major; Ref, reference group; VTE, venous thromboembolism. Table B. Incidence rate per 100 person-years by outcome (pooled analysis). CRNM, clinically relevant non-major; VTE, venous thromboembolism. Table C. Adjusted hazard ratios using an intention-to-treat approach (pooled analysis). CI, confidence interval; CRNM, clinically relevant non-major; Ref, reference group; VTE, venous thromboembolism. Table D. Results from additional sensitivity analyses. *Sensitivity analysis using alternative exclusion windows of seven days for defining recurrent VTE, instead of the 30-day window used in the primary analysis. Table E. List of inclusion and exclusion criteria codes. CPT, Current Procedural Terminology; HCPCS, Healthcare Common Procedure Coding System; ICD-10-CM, International Classification of Diseases, 10th Revision, Clinical Modification; ICD-10-PCS, International Classification of Diseases, 10th Revision, Procedure Coding System; VTE, venous thromboembolism. Table F. List of chemotherapy agents, chemotherapy administration, and radiation therapy codes. CPT, Current Procedural Terminology; HCPCS, Healthcare Common Procedure Coding System; ICD-10-CM, International Classification of Diseases, 10th Revision, Clinical Modification; ICD-10-PCS, International Classification of Diseases, 10th Revision, Procedure Coding System; IV, intravenous; NDC, National Drug Code. Table G. List of baseline covariates. AIDS, acquired immunodeficiency syndrome; COPD, chronic obstructive pulmonary disease; CPT, Current Procedural Terminology; GI, gastrointestinal; HCPCS, Healthcare Common Procedure Coding System; ICD-10-CM, International Classification of Diseases, 10th Revision, Clinical Modification; ICD-10-PCS, International Classification of Diseases, 10th Revision, Procedure Coding System. Table H. List of baseline medication codes. *NDC codes will be used wherever [file pmed.1004754.s001.docx]

**Supporting information**

*Comparative safety and effectiveness of apixaban and rivaroxaban for treatment of cancer-associated venous thromboembolism: A retrospective cohort study*

## Table of contents

Table of contents

[Table of contents 1](#_Toc208234057)

[Additional supporting figures and tables 2](#_Toc208234058)

[Figure A. Graphical depiction of study design 2](#_Toc208234059)

[Figure B. MarketScan and Medicare treatment patterns. 3](#_Toc208234060)

[Figure C. Adjusted Kaplan-Meier curves for major bleeding by database and follow-up time 4](#_Toc208234061)

[Figure D. Adjusted Kaplan-Meier curves for clinically relevant non-major bleeding by database and follow-up time 5](#_Toc208234062)

[Figure E. Adjusted Kaplan-Meier curves for recurrent VTE by database and follow-up time 6](#_Toc208234063)

[Table A. Fine Gray risk regression outcomes for Medicare database 7](#_Toc208234064)

[Table B. Incidence rate per 100 person-years by outcome (pooled analysis) 8](#_Toc208234065)

[Table C. Adjusted hazard ratios using an intention-to-treat approach (pooled analysis) 9](#_Toc208234066)

[Table D. Results from additional sensitivity analyses 9](#_Toc208234067)

[List of ICD-10 diagnosis and procedure codes, CPT and HCPCS codes 10](#_Toc208234068)

[Table E. List of inclusion and exclusion criteria codes 10](#_Toc208234069)

[Table F. List of chemotherapy agents, chemotherapy administration, and radiation therapy codes 15](#_Toc208234070)

[Table G. List of baseline covariates 26](#_Toc208234071)

[Table H. List of baseline medication codes 38](#_Toc208234072)

[Table I. List of major bleeding, clinically relevant non-major bleeding, and recurrent VTE codes 40](#_Toc208234073)

[Table J. List of oral and parenteral anticoagulation codes 42](#_Toc208234074)

[References 43](#_Toc208234075)

## Additional supporting figures and tables

Figure A. Graphical depiction of study design


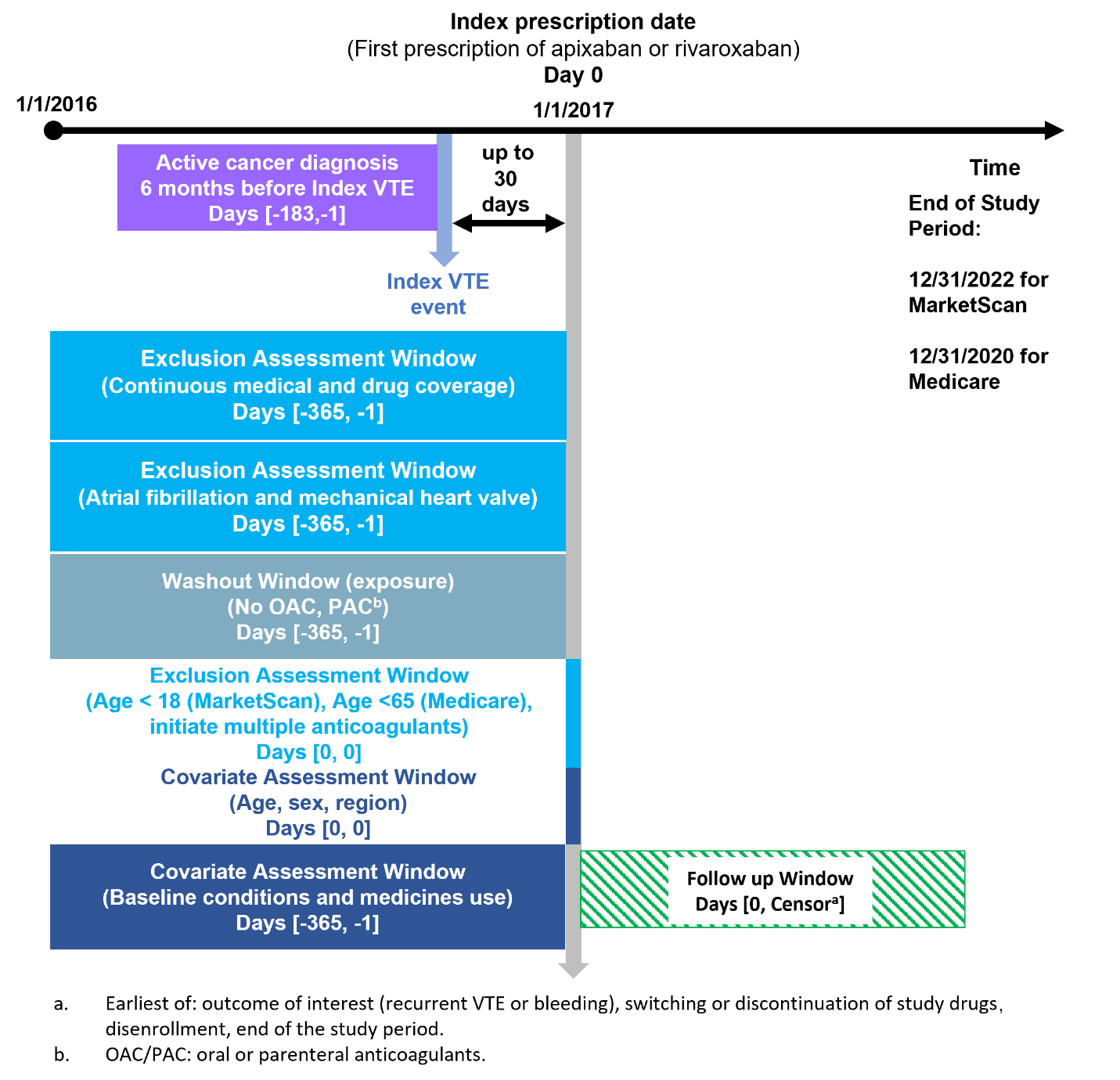


Figure B. MarketScan and Medicare treatment patterns.


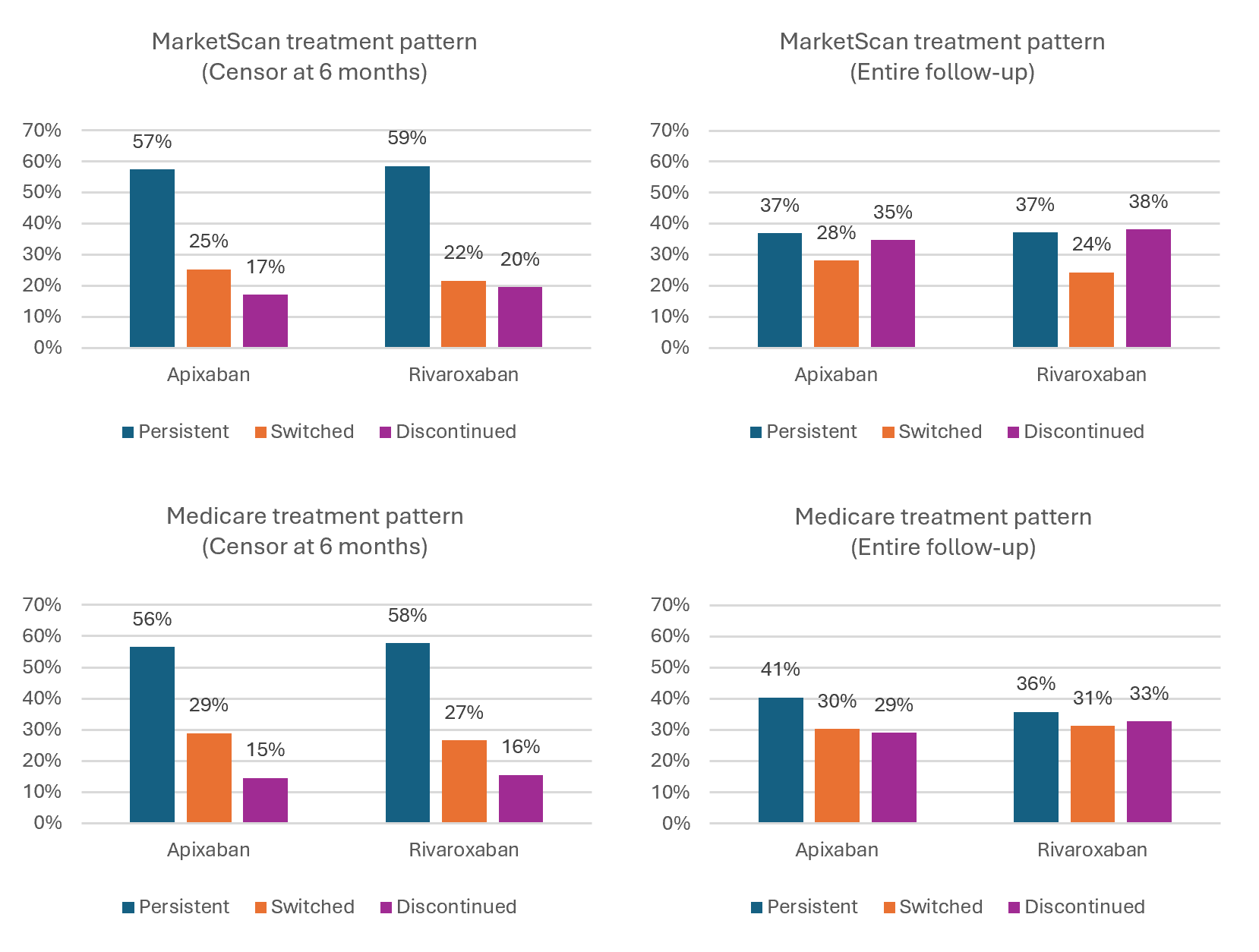


Figure C. Adjusted Kaplan-Meier curves for major bleeding by database and follow-up time


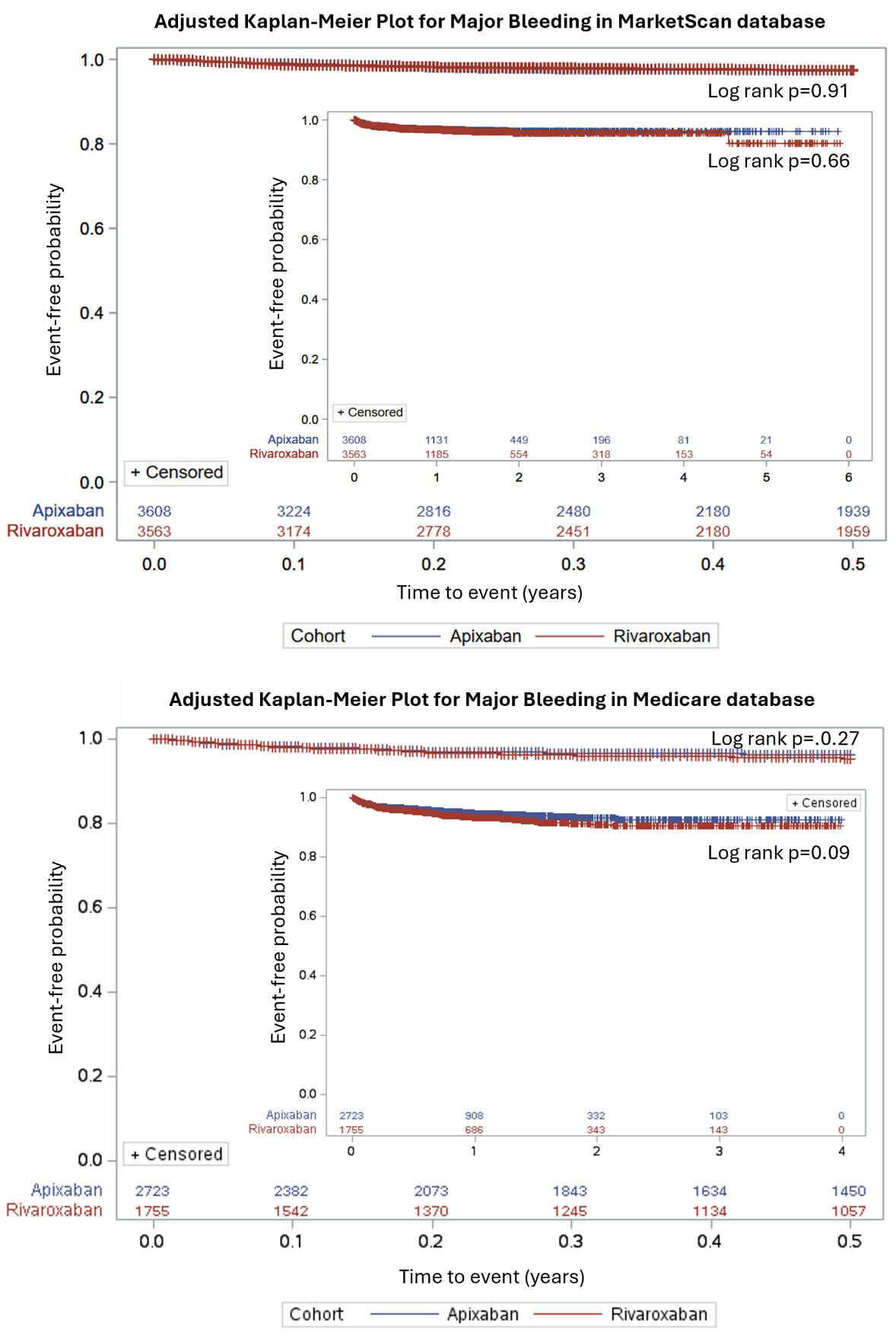


Figure D. Adjusted Kaplan-Meier curves for clinically relevant non-major bleeding by database and follow-up time


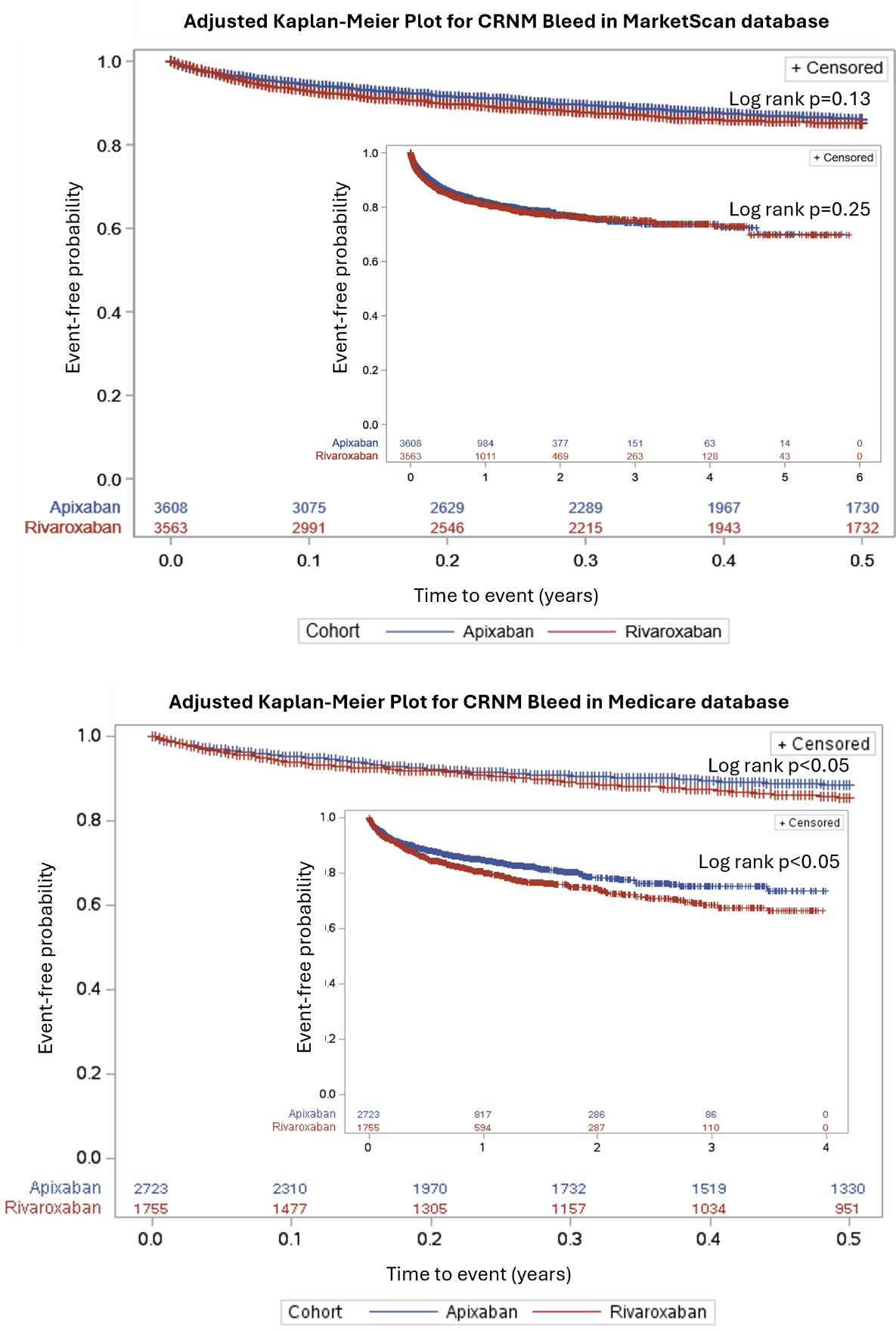


Figure E. Adjusted Kaplan-Meier curves for recurrent VTE by database and follow-up time

***
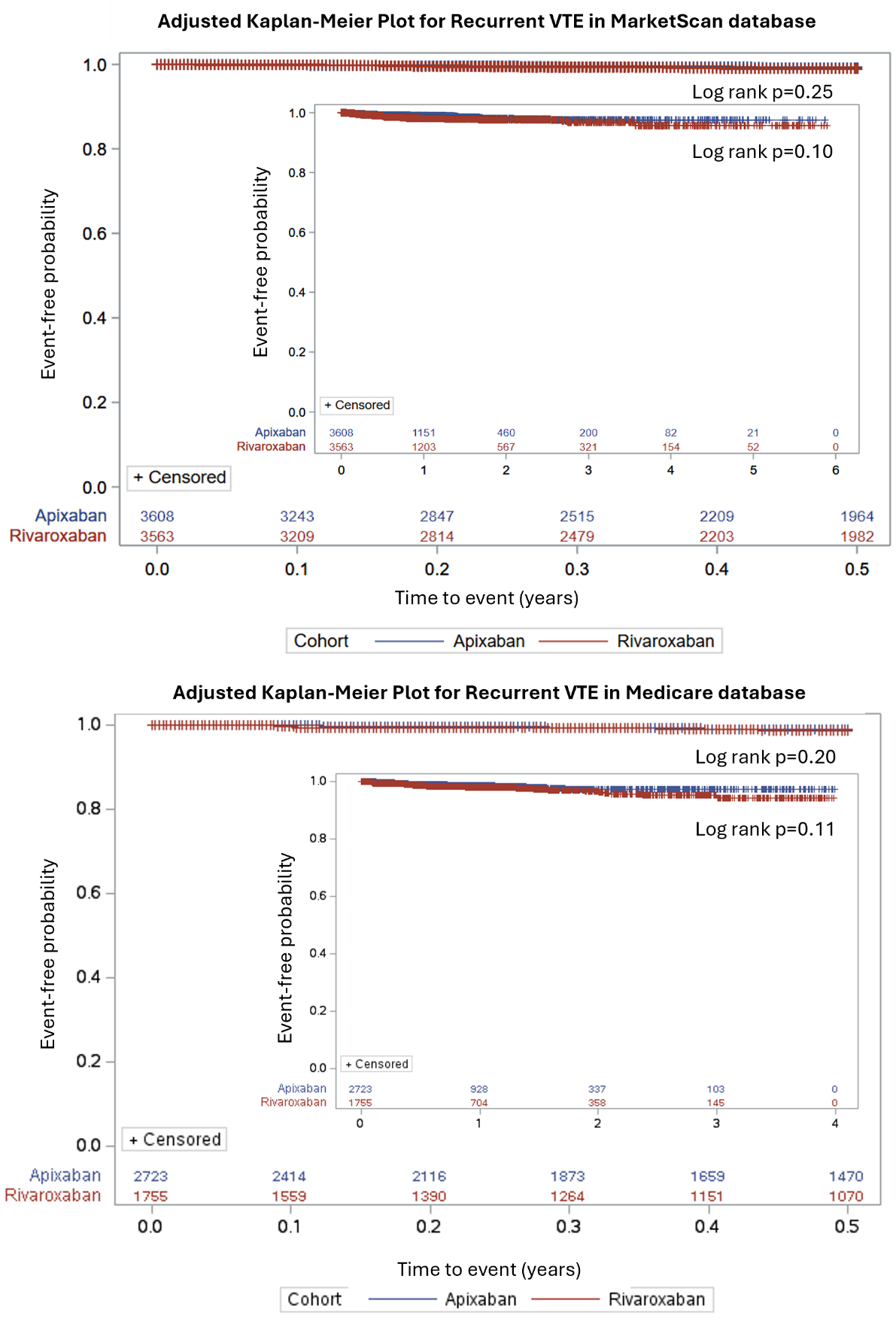
***

Table A. Fine Gray risk regression outcomes for Medicare database

| **Outcome (Apixaban vs. Rivaroxaban [Ref])** | **Hazard Ratio** | **95% CI lower** | **95% CI upper** |
| --- | --- | --- | --- |
| **Censored at 6 Months** |  |  |  |
| Major Bleeding | 0.82 | 0.60 | 1.11 |
| Gastrointestinal Major Bleeding | 0.71 | 0.44 | 1.15 |
| Intracranial Major Bleeding | 0.35 | 0.12 | 1.06 |
| Other Major Bleeding | 1.21 | 0.81 | 1.80 |
| CRNM bleeding | 0.73 | 0.61 | 0.87 |
| Gastrointestinal CRNM Bleeding | 0.73 | 0.56 | 0.97 |
| Other CRNM Bleeding | 0.71 | 0.57 | 0.88 |
| Recurrent VTE | 0.62 | 0.35 | 1.10 |
| **Entire follow-up** |  |  |  |
| Major Bleeding | 0.78 | 0.60 | 1.01 |
| Gastrointestinal Major Bleeding | 0.75 | 0.51 | 1.10 |
| Intracranial Major Bleeding | 0.61 | 0.26 | 1.45 |
| Other Major Bleeding | 0.85 | 0.61 | 1.20 |
| CRNM Bleeding | 0.71 | 0.62 | 0.83 |
| Gastrointestinal CRNM Bleeding | 0.86 | 0.68 | 1.08 |
| Other CRNM Bleeding | 0.67 | 0.56 | 0.80 |
| Recurrent VTE | 0.71 | 0.44 | 1.15 |

CI: confidence interval; CRNM: clinically relevant non-major; Ref: reference group; VTE: venous thromboembolism.

Table B. Incidence rate per 100 person-years by outcome (pooled analysis)

| **Event** | **Cohort** | **Number of events** | **Incidence rate per 100 person-years** |
| --- | --- | --- | --- |
| **Censored at 6 months** |  |  |  |
| Major Bleeding | Apixaban | 185.5 | 7.44 |
| Major Bleeding | Rivaroxaban | 156.8 | 7.36 |
| Gastrointestinal Major Bleeding | Apixaban | 65.3 | 2.59 |
| Gastrointestinal Major Bleeding | Rivaroxaban | 58.4 | 2.72 |
| Intracranial Major Bleeding | Apixaban | 8.9 | 0.35 |
| Intracranial Major Bleeding | Rivaroxaban | 14.9 | 0.69 |
| Other Major Bleeding | Apixaban | 125.9 | 5.03 |
| Other Major Bleeding | Rivaroxaban | 93.7 | 4.38 |
| CRNM Bleeding | Apixaban | 719.3 | 30.54 |
| CRNM Bleeding | Rivaroxaban | 720.5 | 36.43 |
| Gastrointestinal CRNM Bleeding | Apixaban | 266.8 | 10.78 |
| Gastrointestinal CRNM Bleeding | Rivaroxaban | 245.1 | 11.64 |
| Other CRNM Bleeding | Apixaban | 489.8 | 20.38 |
| Other CRNM Bleeding | Rivaroxaban | 509.6 | 25.17 |
| Recurrent VTE | Apixaban | 43.0 | 1.71 |
| Recurrent VTE | Rivaroxaban | 53.9 | 2.50 |
| **Entire follow-up** |  |  |  |
| Major Bleeding | Apixaban | 245.4 | 4.00 |
| Major Bleeding | Rivaroxaban | 218.6 | 3.63 |
| Gastrointestinal Major Bleeding | Apixaban | 91.2 | 1.47 |
| Gastrointestinal Major Bleeding | Rivaroxaban | 82.2 | 1.35 |
| Intracranial Major Bleeding | Apixaban | 16.3 | 0.26 |
| Intracranial Major Bleeding | Rivaroxaban | 17.9 | 0.29 |
| Other Major Bleeding | Apixaban | 156.1 | 2.53 |
| Other Major Bleeding | Rivaroxaban | 141.1 | 2.33 |
| CRNM Bleeding | Apixaban | 954.0 | 17.20 |
| CRNM Bleeding | Rivaroxaban | 963.2 | 18.33 |
| Gastrointestinal CRNM Bleeding | Apixaban | 376.5 | 6.21 |
| Gastrointestinal CRNM Bleeding | Rivaroxaban | 337.4 | 5.72 |
| Other CRNM Bleeding | Apixaban | 646.3 | 11.32 |
| Other CRNM Bleeding | Rivaroxaban | 685.8 | 12.54 |
| Recurrent VTE | Apixaban | 76.8 | 1.23 |
| Recurrent VTE | Rivaroxaban | 98.3 | 1.61 |

CRNM: clinically relevant non-major; VTE: venous thromboembolism.

Table C. Adjusted hazard ratios using an intention-to-treat approach (pooled analysis)

| **Outcome (Apixaban vs. Rivaroxaban [Ref])** | **Hazard Ratio** | **95% CI lower** | **95% CI upper** | **p-value for heterogeneity** | **I^2^** |
| --- | --- | --- | --- | --- | --- |
| **Censored at 6 Months** |  |  |  |  |  |
| Major Bleeding | 0.90 | 0.70 | 1.16 | 0.54 | 0.0 |
| Gastrointestinal Major Bleeding | 0.80 | 0.52 | 1.25 | 0.66 | 0.0 |
| Intracranial Major Bleeding | 0.45 | 0.19 | 1.07 | 0.93 | 0.0 |
| Other Major Bleeding | 1.06 | 0.77 | 1.45 | 0.93 | 0.0 |
| CRNM bleeding | 0.85 | 0.75 | 0.96 | 0.40 | 0.0 |
| Gastrointestinal CRNM Bleeding | 0.91 | 0.74 | 1.12 | 0.39 | 0.0 |
| Other CRNM Bleeding | 0.83 | 0.71 | 0.96 | 0.42 | 0.0 |
| Recurrent VTE | 0.68 | 0.42 | 1.09 | 0.91 | 0.0 |
| **Entire follow-up** |  |  |  |  |  |
| Major Bleeding | 0.90 | 0.72 | 1.12 | 0.99 | 0.0 |
| Gastrointestinal Major Bleeding | 0.87 | 0.59 | 1.28 | 0.73 | 0.0 |
| Intracranial Major Bleeding | 0.60 | 0.29 | 1.21 | 0.98 | 0.0 |
| Other Major Bleeding | 0.90 | 0.69 | 1.17 | 0.90 | 0.0 |
| CRNM Bleeding | 0.86 | 0.77 | 0.96 | 0.17 | 28.0 |
| Gastrointestinal CRNM Bleeding | 0.93 | 0.78 | 1.10 | 0.53 | 0.0 |
| Other CRNM Bleeding | 0.84 | 0.74 | 0.96 | 0.23 | 21.9 |
| Recurrent VTE | 0.75 | 0.54 | 1.03 | 0.57 | 0.0 |

CI: confidence interval; CRNM: clinically relevant non-major; Ref: reference group; VTE: venous thromboembolism.

Table D. Results from additional sensitivity analyses

| **Outcome (Apixaban vs. Rivaroxaban [Ref])** | **Hazard Ratio** | **95% CI lower** | **95% CI upper** |
| --- | --- | --- | --- |
| **Censored at 6 Months** |  |  |  |
| Recurrent VTE outcome using 7-day exclusion window* | 0.80 | 0.57 | 1.12 |
| **Entire follow-up** |  |  |  |
| Recurrent VTE outcome using 7-day exclusion window* | 0.79 | 0.59 | 1.06 |

*Sensitivity analysis using alternative exclusion windows of seven days for defining recurrent VTE, instead of the 30-day window used in the primary analysis.

## List of ICD-10 diagnosis and procedure codes, CPT and HCPCS codes

Table E. List of inclusion and exclusion criteria codes

| **Diagnosis/Procedure** | **Type of Code** | **Codes** |
| --- | --- | --- |
| Atrial fibrillation[[1](#_ENREF_1), [2](#_ENREF_2)] | ICD-10-CM | I480, I482, I481, I483, I484, I4891, I4892 |
| Mechanical Heart Valve[[1](#_ENREF_1), [2](#_ENREF_2)] | ICD-10-PCS | 02RF07Z, 02RF08Z, 02RF0JZ, 02RF0KZ, 02RF37H, 02RF37Z, 02RF38H, 02RF38Z, 02RF3JH, 02RF3JZ, 02RF3KH, 02RF3KZ, 02RF47Z, 02RF48Z, 02RF4JZ, 02RF4KZ, 02RG07Z, 02RG08Z, 02RG0JZ, 02RG0KZ, 02RG37H, 02RG37Z, 02RG38H, 02RG38Z, 02RG3JH, 02RG3JZ, 02RG3KH, 02RG3KZ, 02RG47Z, 02RG48Z, 02RG4JZ, 02RG4KZ, 02RH07Z, 02RH08Z, 02RH0JZ, 02RH0KZ, 02RH37H, 02RH37Z, 02RH38H, 02RH38Z, 02RH3JH, 02RH3JZ, 02RH3KH, 02RH3KZ, 02RH47Z, 02RH48Z, 02RH4JZ, 02RH4KZ, 02RJ07Z, 02RJ08Z, 02RJ0JZ, 02RJ0KZ, 02RJ47Z, 02RJ48Z, 02RJ4JZ, 02RJ4KZ, 02UG3JZ, X2RF032, X2RF332, X2RF432 |
| Venous thromboembolism (VTE)[[3](#_ENREF_3)] | ICD-10-CM | I26.0*, I26.9*, I80.1*, I80.20*, I82.210, I80.22*, I80.23*, I80.29*, I82.40*, I82.41*, I82.42*, I82.43*, I82.44*, I82.49*, I82.4Y*, I82.4Z*, I82.60*, I82.62*, I82.890, I82.A1*, I82.B1*, I82.C1* |
| Malignant Neoplasm (excluding non-melanoma skin) [[1](#_ENREF_1)] [[2](#_ENREF_2)] | ICD-10-CM | C01, C07, C12, C19, C20, C23, C33, C37, C55, C58, C52, C61, C73, D45, C000, C001, C003, C004, C005, C006, C008, C002, C009, C020, C021, C022, C023, C028, C024, C029, C080, C081, C089, C030, C031, C039, C040, C041, C048, C049, C060, C061, C050, C051, C052, C058, C059, C062, C069, C098, C099, C090, C091, C100, C101, C108, C102, C103, C104, C109, C110, C111, C112, C113, C118, C119, C130, C131, C132, C138, C139, C140, C142, C148, C153, C154, C155, C158, C159, C160, C164, C163, C161, C162, C165, C166, C168, C169, C170, C171, C172, C173, C178, C179, C183, C184, C186, C187, C180, C181, C182, C185, C188, C189, C211, C210, C212, C218, C220, C222, C223, C224, C227, C228, C221, C229, C240, C241, C248, C249, C250, C251, C252, C253, C254, C257, C258, C259, C480, C451, C481, C488, C482, C260, C261, C269, C300, C301, C310, C311, C312, C313, C318, C319, C320, C321, C322, C323, C328, C329, C342, C384, C450, C380, C452, C381, C382, C388, C383, C390, C399, C410, C411, C412, C413, C414, C419, C470, C490, C473, C493, C474, C494, C475, C495, C476, C496, C478, C498, C479, C499, C430, C434, C438, C439, C460, C461, C462, C464, C463, C467, C469, C530, C531, C538, C539, C541, C542, C543, C549, C540, C548, C561, C562, C569, C573, C574, C510, C511, C512, C519, C518, C577, C578, C579, C600, C601, C602, C609, C632, C608, C637, C638, C639, C670, C671, C672, C673, C674, C675, C676, C677, C678, C679, C641, C642, C649, C651, C652, C659, C661, C662, C669, C680, C681, C688, C689, C710, C711, C712, C713, C714, C715, C716, C717, C718, C719, C700, C709, C720, C721, C701, C729, C750, C751, C752, C753, C754, C755, C758, C759, C760, C761, C762, C763, C457, C768, C770, C771, C772, C773, C774, C775, C778, C779, C781, C782, C784, C785, C786, C787, C792, C799, C800, C459, C801, C802, C884, C865, C866, C96A, C960, C962, C864, C860, C862, C863, C861, C964, C969, C96Z, C882, C883, C888, C889, C7A1, C7A8, C4A0, C4A4, C4A8, C4A9, C0680, C0689, C3400, C3401, C3402, C3410, C3411, C3412, C3430, C3431, C3432, C3480, C3481, C3482, C3490, C3491, C3492, C4000, C4001, C4002, C4010, C4011, C4012, C4020, C4021, C4022, C4030, C4031, C4032, C4080, C4081, C4082, C4090, C4091, C4092, C4710, C4711, C4712, C4910, C4911, C4912, C4720, C4721, C4722, C4920, C4921, C4922, C4310, C4311, C4312, C4320, C4321, C4322, C4330, C4331, C4339, C4351, C4352, C4359, C4360, C4361, C4362, C4370, C4371, C4372, C4650, C4651, C4652, C5700, C5701, C5702, C5710, C5711, C5712, C5720, C5721, C5722, C6200, C6201, C6202, C6210, C6211, C6212, C6290, C6291, C6292, C6300, C6301, C6302, C6310, C6311, C6312, C6940, C6941, C6942, C6960, C6961, C6962, C6950, C6951, C6952, C6900, C6901, C6902, C6910, C6911, C6912, C6920, C6921, C6922, C6930, C6931, C6932, C6980, C6981, C6982, C6990, C6991, C6992, C7220, C7221, C7222, C7230, C7231, C7232, C7240, C7241, C7242, C7250, C7259, C7400, C7401, C7402, C7410, C7411, C7412, C7490, C7491, C7492, C7640, C7641, C7642, C7650, C7651, C7652, C7800, C7801, C7802, C7830, C7839, C7880, C7889, C7900, C7901, C7902, C7910, C7911, C7919, C7931, C7932, C7940, C7949, C7951, C7952, C7960, C7961, C7962, C7970, C7971, C7972, C7981, C7982, C7989, C8330, C8339, C8331, C8332, C8333, C8334, C8335, C8336, C8337, C8338, C8350, C8359, C8351, C8352, C8353, C8354, C8355, C8356, C8357, C8358, C8370, C8379, C8371, C8372, C8373, C8374, C8375, C8376, C8377, C8378, C8380, C8389, C8381, C8382, C8383, C8384, C8385, C8386, C8387, C8388, C8310, C8319, C8311, C8312, C8313, C8314, C8315, C8316, C8317, C8318, C8460, C8469, C8470, C8479, C8461, C8471, C8462, C8472, C8463, C8473, C8464, C8474, C8465, C8475, C8466, C8476, C8467, C8477, C8468, C8478, C8520, C8529, C8521, C8522, C8523, C8524, C8525, C8526, C8527, C8528, C8300, C8309, C8390, C8399, C8301, C8391, C8302, C8392, C8303, C8393, C8304, C8394, C8305, C8395, C8306, C8396, C8307, C8397, C8308, C8398, C8170, C8179, C8171, C8172, C8173, C8174, C8175, C8176, C8177, C8178, C8100, C8109, C8140, C8149, C8101, C8141, C8102, C8142, C8103, C8143, C8104, C8144, C8105, C8145, C8106, C8146, C8107, C8147, C8108, C8148, C8110, C8119, C8111, C8112, C8113, C8114, C8115, C8116, C8117, C8118, C8120, C8129, C8121, C8122, C8123, C8124, C8125, C8126, C8127, C8128, C8130, C8139, C8131, C8132, C8133, C8134, C8135, C8136, C8137, C8138, C8190, C8199, C8191, C8192, C8193, C8194, C8195, C8196, C8197, C8198, C8200, C8209, C8210, C8219, C8220, C8229, C8230, C8239, C8240, C8249, C8260, C8269, C8280, C8289, C8290, C8299, C8201, C8211, C8221, C8231, C8241, C8261, C8281, C8291, C8202, C8212, C8222, C8232, C8242, C8262, C8282, C8292, C8203, C8213, C8223, C8233, C8243, C8263, C8283, C8293, C8204, C8214, C8224, C8234, C8244, C8264, C8284, C8294, C8205, C8215, C8225, C8235, C8245, C8265, C8285, C8295, C8206, C8216, C8226, C8236, C8246, C8266, C8286, C8296, C8207, C8217, C8227, C8237, C8247, C8267, C8287, C8297, C8208, C8218, C8228, C8238, C8248, C8268, C8288, C8298, C8400, C8409, C8401, C8402, C8403, C8404, C8405, C8406, C8407, C8408, C8410, C8419, C8411, C8412, C8413, C8414, C8415, C8416, C8417, C8418, C9140, C9141, C9142, C8440, C8449, C8441, C8442, C8443, C8444, C8445, C8446, C8447, C8448, C8250, C8259, C8490, C8499, C84A0, C84A9, C84Z0, C84Z9, C8510, C8519, C8580, C8589, C8590, C8599, C8251, C8491, C84A1, C84Z1, C8511, C8581, C8591, C8252, C8492, C84A2, C84Z2, C8512, C8582, C8592, C8253, C8493, C84A3, C84Z3, C8513, C8583, C8593, C8254, C8494, C84A4, C84Z4, C8514, C8584, C8594, C8255, C8495, C84A5, C84Z5, C8515, C8585, C8595, C8256, C8496, C84A6, C84Z6, C8516, C8586, C8596, C8257, C8497, C84A7, C84Z7, C8517, C8587, C8597, C8258, C8498, C84A8, C84Z8, C8518, C8588, C8598, C9000, C9001, C9002, C9010, C9011, C9012, C9020, C9030, C9021, C9031, C9022, C9032, C9100, C9101, C9102, C9110, C9111, C9112, C91Z0, C91Z1, C91Z2, C9130, C9150, C9160, C91A0, C9131, C9151, C9161, C91A1, C9132, C9152, C9162, C91A2, C9190, C9191, C9192, C9200, C9240, C9250, C9260, C92A0, C9201, C9241, C9251, C9261, C92A1, C9202, C9242, C9252, C9262, C92A2, C9210, C9211, C9212, C9220, C9221, C9222, C9230, C9231, C9232, C92Z0, C92Z1, C92Z2, C9290, C9291, C9292, C9300, C9301, C9302, C9310, C9311, C9312, C9390, C9391, C9392, C9330, C93Z0, C9331, C93Z1, C9332, C93Z2, C9400, C9401, C9402, C9420, C9421, C9422, C9430, C9480, C9431, C9481, C9432, C9482, C9500, C9501, C9502, C9510, C9511, C9512, C9590, C9591, C9592, C7A00, C4A10, C4A11, C4A12, C4A20, C4A21, C4A22, C4A30, C4A31, C4A39, C4A60, C4A61, C4A62, C4A70, C4A71, C4A72, C4A51, C4A52, C4A59, C50011, C50012, C50019, C50111, C50112, C50119, C50211, C50212, C50219, C50311, C50312, C50319, C50411, C50412, C50419, C50511, C50512, C50519, C50611, C50612, C50619, C50811, C50812, C50819, C50911, C50912, C50919, C50021, C50022, C50029, C50121, C50122, C50129, C50221, C50222, C50229, C50321, C50322, C50329, C50421, C50422, C50429, C50521, C50522, C50529, C50621, C50622, C50629, C50821, C50822, C50829, C50921, C50922, C50929, C7A019, C7A010, C7A011, C7A012, C7A029, C7A020, C7A021, C7A022, C7A023, C7A024, C7A025, C7A026, C7A090, C7A091, C7A092, C7A093, C7A094, C7A095, C7A096, C7A098 |
| Inferior Vena Cava Filter[[1](#_ENREF_1)] [[2](#_ENREF_2)] | ICD-10-PCS | 02HV0DZ, 02HV3DZ, 02HV4DZ, 02LV0CZ, 02LV0DZ, 02LV0ZZ, 02LV3CZ, 02LV3DZ, 02LV3ZZ, 02LV4CZ, 02LV4DZ, 02LV4ZZ, 02VV0CZ, 02VV0DZ, 02VV0ZZ, 02VV3CZ, 02VV3DZ, 02VV3ZZ, 02VV4CZ, 02VV4DZ, 02VV4ZZ, 06H00DZ, 06H03DZ, 06H04DZ, 06L00CZ, 06L00DZ, 06L00ZZ, 06L03CZ, 06L03DZ, 06L03ZZ, 06L04CZ, 06L04DZ, 06L04ZZ, 06V00CZ, 06V00DZ, 06V00ZZ, 06V03CZ, 06V03DZ, 06V03ZZ, 06V04CZ, 06V04DZ, 06V04ZZ |
|  | CPT | 37191, 37192, 37193, 37620 |
| Cancer-related surgery[[1](#_ENREF_1)] [[2](#_ENREF_2)] | ICD-10-PCS | 0HBT7ZZ, 0HBT8ZZ, 0HBTXZZ, ,0HBU7ZZ, 0HBU8ZZ, 0HBUXZZ, 0HBV7ZZ, 0HBV8ZZ, 0HBVXZZ, 0HBT0ZZ, 0HBT3ZZ, 0HBU0ZZ, 0HBU3ZZ, 0HBV0ZZ, 0HBV3ZZ, 0HBT0ZZ, 0HBT3ZZ, 0HBU0ZZ, 0HBU3ZZ, 0HBV0ZZ, 0HBV3ZZ, 0H0T0JZ, 0H0T3JZ, 0H0U0JZ, 0H0U3JZ, 0HRT0JZ, 0HRT3JZ, 0HRU0JZ, 0HRU3JZ, 0HRV0JZ, 0HRV3JZ, 0HBT0ZZ, 0HBT3ZZ, 0HBU0ZZ, 0HBU3ZZ, 0H0V0JZ, 0H0V3JZ, 0HBV0ZZ, 0HBV3ZZ, 0HTT0ZZ, 0HTU0ZZ, 0HTV0ZZ, 07T50ZZ, 07T60ZZ, 0HTT0ZZ, 0HTU0ZZ, 07T50ZZ, 07T60ZZ, 0HTV0ZZ, 07T50ZZ, 07T60ZZ, 0HTT0ZZ, 0HTU0ZZ, 0KTH0ZZ, 0KTJ0ZZ, 07T50ZZ, 07T60ZZ, 0HTV0ZZ, 0KTH0ZZ, 0KTJ0ZZ, 07T50ZZ, 07T60ZZ, 07T70ZZ, 07T70ZZ, 07T80ZZ, 07T90ZZ, 0HTT0ZZ, 0HTU0ZZ, 0KTH0ZZ, 0KTJ0ZZ, 07T50ZZ, 07T60ZZ, 07T70ZZ, 07T80ZZ, 07T90ZZ, 0HTV0ZZ, 0KTH0ZZ, 0KTJ0ZZ, 3E03305, 3E04305, 3E00X0M, 3E0130M, 3E0230M, 3E03303, 3E0330M, 3E04303, 3E0430M, 3E05303, 3E0530M, 3E06303, 3E0630M, DWY18ZZ, DWY28ZZ, DWY38ZZ, DWY48ZZ, DWY58ZZ, DWY68ZZ |
|  | CPT | 19120, 19125, 19126, 19301, 19302-19307, 19140, 19160, 19162, 19180, 19182, 19200, 19220, 19240, 19260, 19271, 19272, 19300, 19301, 19302, 19303, 19304, 19305, 19306, 19307, 19316, 19318, 19324, 19325, 19357, 19361, 19364, 19366, 19367, 19368, 19369, 21015, 21016, 21034, 21044, 21045, 21557, 21935, 21936, 23200, 23210, 23220, 23221, 23222, 24077, 24079, 24150, 24151, 24152, 24153, 25077, 25170, 26117, 26250, 26255, 26260, 26261, 26262, 27049, 27075, 27076, 27077, 27078, 27079, 27329, 27365, 27615, 27645, 27646, 27647, 28046, 28171, 28173, 28175, 31785, 31786, 32503, 32504, 33050, 33120, 33130, 45160, 45170, 47711, 47712, 49200, 49201, 49215, 54530, 54535, 58950, 58951, 58952, 58957, 58958, 58960, 60252, 60254, 60600, 60605, 61510, 61517, 61518, 61519, 61520, 61521, 61526, 61530, 61546, 61548, 63275, 63276, 63277, 63278, 63280, 63281, 63282, 63283, 63285, 63286, 63287, 63290 |
| Pregnancy codes[[1](#_ENREF_1), [2](#_ENREF_2)] | ICD-10-CM | O00.*, O01.*, O02.*, O03.*, O04.*, O07.*, O08.*, O09.*, O10.*, O11.*, O12.*, O13.*, O14.*, O15.*, O16.*, O20.*, O21.*, O22.*, O23.*, O24.*, O25.*, O26.*, O28.*, O29.*, O30.*, O31.*, O32.*, O33.*, O34.*, O35.*, O36.*, O40.*, O41.*, O42.*, O43.*, O44.*, O45.*, O46.*, O47.*, O48.*, O60.*, O61.*, O62.*, O63.*, O64.*, O65.*, O66.*, O67.*, O68, O69.*, O70.*, O71.*, O72.*, O73.*, O74.*, O75.*  , O76, O77.*, O80, O82, O85, O86.*, O87.*, O88.*, O89.*, O90.*, O91.*, O92.*, O94, O98.*, O99.*, O9A.*, Z33.1, Z33.2, Z34.*, Z36, Z37.*, Z64.0 (see detailed list: [1, 2]) |
|  | ICD-10-PCS | 0DQP0ZZ, 0DQP3ZZ, 0DQP4ZZ, 0DQP7ZZ, 0DQP8ZZ, 0DQR0ZZ, 0DQR3ZZ, 0DQR4ZZ, 0JCB0ZZ, 0JCB3ZZ, 0Q820ZZ, 0Q823ZZ, 0Q824ZZ, 0Q830ZZ, 0Q833ZZ, 0Q834ZZ, 0TQB0ZZ, 0TQB3ZZ, 0TQB4ZZ, 0TQB7ZZ, 0TQB8ZZ, 0TQD0ZZ, 0TQD3ZZ, 0TQD4ZZ, 0TQD7ZZ, 0TQD8ZZ, 0TQDXZZ, 0U7C7ZZ, 0UCG0ZZ, 0UCG3ZZ, 0UCG4ZZ, 0UCM0ZZ, 0UJD7ZZ, 0UQ90ZZ, 0UQ93ZZ, 0UQ94ZZ, 0UQ97ZZ, 0UQ98ZZ, 0UQC0ZZ, 0UQC3ZZ, 0UQC4ZZ, 0UQC7ZZ, 0UQC8ZZ, 0UQG0ZZ, 0UQG3ZZ, 0UQG4ZZ, 0UQG7ZZ, 0UQG8ZZ, 0UQGXZZ, 0UQM0ZZ, 0UQMXZZ, 0US90ZZ, 0US94ZZ, 0US9XZZ, 0W3R0ZZ, 0W3R3ZZ, 0W3R4ZZ, 0W3R7ZZ, 0W3R8ZZ, 0W8NXZZ, 0WQNXZZ, 10900Z9, 10900ZA, 10900ZB, 10900ZC, 10900ZD, 10900ZU, 10903Z9, 10903ZA, 10903ZB, 10903ZC, 10903ZD, 10903ZU, 10904Z9, 10904ZA, 10904ZB, 10904ZC, 10904ZD, 10904ZU, 10907Z9, 10907ZA, 10907ZB, 10907ZC, 10907ZD, 10907ZU, 10908Z9, 10908ZA, 10908ZB, 10908ZC, 10908ZD, 10908ZU, 10A00ZZ, 10A03ZZ, 10A04ZZ, 10A07Z6, 10A07ZX, 10A07ZZ, 10A08ZZ, 10D00Z0, 10D00Z1, 10D00Z2, 10D07Z3, 10D07Z4, 10D07Z5, 10D07Z6, 10D07Z7, 10D07Z8, 10D17ZZ, 10D18ZZ, 10E0XZZ, 10H003Z, 10H00YZ, 10H073Z, 10H07YZ, 10J00ZZ, 10J03ZZ, 10J04ZZ, 10J07ZZ, 10J08ZZ, 10J0XZZ, 10J10ZZ, 10J13ZZ, 10J14ZZ, 10J17ZZ, 10J18ZZ, 10J1XZZ, 10J20ZZ, 10J23ZZ, 10J24ZZ, 10J27ZZ, 10J28ZZ, 10J2XZZ, 10P003Z, 10P00YZ, 10P073Z, 10P07YZ, 10Q00YE, 10Q00YF, 10Q00YG, 10Q00YH, 10Q00YJ, 10Q00YK, 10Q00YL, 10Q00YM, 10Q00YN, 10Q00YP, 10Q00YQ, 10Q00YR, 10Q00YS, 10Q00YT, 10Q00YV, 10Q00YY, 10Q00ZE, 10Q00ZF, 10Q00ZG, 10Q00ZH, 10Q00ZJ, 10Q00ZK, 10Q00ZL, 10Q00ZM, 10Q00ZN, 10Q00ZP, 10Q00ZQ, 10Q00ZR, 10Q00ZS, 10Q00ZT, 10Q00ZV, 10Q00ZY, 10Q03YE, 10Q03YF, 10Q03YG, 10Q03YH, 10Q03YJ, 10Q03YK, 10Q03YL, 10Q03YM, 10Q03YN, 10Q03YP, 10Q03YQ, 10Q03YR, 10Q03YS, 10Q03YT, 10Q03YV, 10Q03YY, 10Q03ZE, 10Q03ZF, 10Q03ZG, 10Q03ZH, 10Q03ZJ, 10Q03ZK, 10Q03ZL, 10Q03ZM, 10Q03ZN, 10Q03ZP, 10Q03ZQ, 10Q03ZR, 10Q03ZS, 10Q03ZT, 10Q03ZV, 10Q03ZY, 10Q04YE, 10Q04YF, 10Q04YG, 10Q04YH, 10Q04YJ, 10Q04YK, 10Q04YL, 10Q04YM, 10Q04YN, 10Q04YP, 10Q04YQ, 10Q04YR, 10Q04YS, 10Q04YT, 10Q04YV, 10Q04YY, 10Q04ZE, 10Q04ZF, 10Q04ZG, 10Q04ZH, 10Q04ZJ, 10Q04ZK, 10Q04ZL, 10Q04ZM, 10Q04ZN, 10Q04ZP, 10Q04ZQ, 10Q04ZR, 10Q04ZS, 10Q04ZT, 10Q04ZV, 10Q04ZY, 10Q07YE, 10Q07YF, 10Q07YG, 10Q07YH, 10Q07YJ, 10Q07YK, 10Q07YL, 10Q07YM, 10Q07YN, 10Q07YP, 10Q07YQ, 10Q07YR, 10Q07YS, 10Q07YT, 10Q07YV, 10Q07YY, 10Q07ZE, 10Q07ZF, 10Q07ZG, 10Q07ZH, 10Q07ZJ, 10Q07ZK, 10Q07ZL, 10Q07ZM, 10Q07ZN, 10Q07ZP, 10Q07ZQ, 10Q07ZR, 10Q07ZS, 10Q07ZT, 10Q07ZV, 10Q07ZY, 10Q08YE, 10Q08YF, 10Q08YG, 10Q08YH, 10Q08YJ, 10Q08YK, 10Q08YL, 10Q08YM, 10Q08YN, 10Q08YP, 10Q08YQ, 10Q08YR, 10Q08YS, 10Q08YT, 10Q08YV, 10Q08YY, 10Q08ZE, 10Q08ZF, 10Q08ZG, 10Q08ZH, 10Q08ZJ, 10Q08ZK, 10Q08ZL, 10Q08ZM, 10Q08ZN, 10Q08ZP, 10Q08ZQ, 10Q08ZR, 10Q08ZS, 10Q08ZT, 10Q08ZV, 10Q08ZY, 10S07ZZ, 10S0XZZ, 10T20ZZ, 10T23ZZ, 10T24ZZ, 10Y03ZE, 10Y03ZF, 10Y03ZG, 10Y03ZH, 10Y03ZJ, 10Y03ZK, 10Y03ZL, 10Y03ZM, 10Y03ZN, 10Y03ZP, 10Y03ZQ, 10Y03ZR, 10Y03ZS, 10Y03ZT, 10Y03ZV, 10Y03ZY, 10Y04ZE, 10Y04ZF, 10Y04ZG, 10Y04ZH, 10Y04ZJ, 10Y04ZK, 10Y04ZL, 10Y04ZM, 10Y04ZN, 10Y04ZP, 10Y04ZQ, 10Y04ZR, 10Y04ZS, 10Y04ZT, 10Y04ZV, 10Y04ZY, 10Y07ZE, 10Y07ZF, 10Y07ZG, 10Y07ZH, 10Y07ZJ, 10Y07ZK, 10Y07ZL, 10Y07ZM, 10Y07ZN, 10Y07ZP, 10Y07ZQ, 10Y07ZR, 10Y07ZS, 10Y07ZT, 10Y07ZV, 10Y07ZY, 2Y44X5Z, 30273H1, 30273J1, 30273K1, 30273L1, 30273M1, 30273N1, 30273P1, 30273Q1, 30273R1, 30273S1, 30273T1, 30273V1, 30273W1, 30277H1, 30277J1, 30277K1, 30277L1, 30277M1, 30277N1, 30277P1, 30277Q1, 30277R1, 30277S1, 30277T1, 30277V1, 30277W1, 3E0DXGC, 3E0E305, 3E0E329, 3E0E33Z, 3E0E36Z, 3E0E37Z, 3E0E3BZ, 3E0E3GC, 3E0E3HZ, 3E0E3KZ, 3E0E3NZ, 3E0E3SF, 3E0E3TZ, 3E0E705, 3E0E729, 3E0E73Z, 3E0E76Z, 3E0E77Z, 3E0E7BZ, 3E0E7GC, 3E0E7HZ, 3E0E7KZ, 3E0E7NZ, 3E0E7SF, 3E0E7TZ, 3E0E805, 3E0E829, 3E0E83Z, 3E0E86Z, 3E0E87Z, 3E0E8BZ, 3E0E8GC, 3E0E8HZ, 3E0E8KZ, 3E0E8NZ, 3E0E8SF, 3E0E8TZ, 3E0P3VZ, 3E0P7VZ , 4A0H74Z, 4A0H7CZ, 4A0H7FZ, 4A0H7HZ, 4A0H84Z, 4A0H8CZ, 4A0H8FZ, 4A0H8HZ, 4A0HX4Z, 4A0HXCZ, 4A0HXFZ, 4A0HXHZ, 4A0J72Z, 4A0J74Z, 4A0J7BZ, 4A0J82Z, 4A0J84Z, 4A0J8BZ, 4A0JX2Z, 4A0JX4Z, 4A0JXBZ, 4A1H74Z, 4A1H7CZ, 4A1H7FZ, 4A1H7HZ, 4A1H84Z, 4A1H8CZ, 4A1H8FZ, 4A1H8HZ, 4A1HX4Z, 4A1HXCZ, 4A1HXFZ, 4A1HXHZ, 4A1J72Z, 4A1J74Z, 4A1J7BZ, 4A1J82Z, 4A1J84Z, 4A1J8BZ, 4A1JX2Z, 4A1JX4Z, 4A1JXBZ |
|  | HCPCS | 59000, 59001, 59012, 59015, 59020, 59025, 59030, 59050, 59051, 59070, 59072, 59074, 59076, 59100, 59120, 59121, 59130, 59135, 59136, 59140, 59150, 59151, 59160, 59200, 59300, 59320, 59325, 59350, 59400, 59409, 59410, 59412, 59414, 59425, 59426, 59430, 59510, 59514, 59515, 59525, 59610, 59612, 59614, 59618, 59620, 59622, 59812, 59820, 59821, 59830, 59840, 59841, 59850, 59851, 59852, 59855, 59856, 59857, 59866, 59870, 59871, 59897, 59898, 59899, 76801, 76802, 76805, 76810, 76811, 76812, 76815, 76816, 76817, 76818, 76819, 76820, 76821, 76825, 76826, 76827, 76828, 83661, 83662, 83663, 83664 |

CPT: Current Procedural Terminology; HCPCS: Healthcare Common Procedure Coding System; ICD-10-CM: International Classification of Diseases, 10th Revision, Clinical Modification; ICD-10-PCS: International Classification of Diseases, 10th Revision, Procedure Coding System; VTE: venous thromboembolism.

Table F. List of chemotherapy agents, chemotherapy administration, and radiation therapy codes

| **Medication[**[**1**](#_ENREF_1)**,** [**2**](#_ENREF_2)**]** | **Type[**[**1**](#_ENREF_1)**,** [**2**](#_ENREF_2)**]** | **HCPCS Codes (use NDC code wherever applicable)** **[**[**1**](#_ENREF_1)**,** [**2**](#_ENREF_2)**]** |
| --- | --- | --- |
| Aldesleukin | IV | J9015 |
| Alemtuzumab | Immunotherapy | J0202, J9010 |
| Amifostine | IV | J0207 |
| Arsenic Trioxide | IV | J9017 |
| Atezolizumab | Immunotherapy | C9483, J9022 |
| Avelumab | Immunotherapy | C9491, J9023 |
| Axicabtagene Ciloleucel | IV | Q2041 |
| Azacitidine | IV | J9025 |
| Bendamustine Hydrochloride | IV | J9033, J9034 |
| Bevacizumab | IV | C9257, J9035 |
| Brentuximab Vedotin | IV | J9042 |
| Busulfan | IV | J0594, J8510 |
| Cabazitaxel | IV | J9043 |
| Capecitabine | IV | J8520, J8521 |
| Carboplatin | IV | J9045 |
| Carfilzomib | IV | J9047 |
| Carmustine | IV | J9050 |
| Cetuximab | IV | J9055 |
| Cisplatin | IV | J9060 |
| Cladribine | IV | J9065 |
| Clofarabine | IV | J9027 |
| Copanlisib Hydrochloride | IV | C9030 |
| Cyclophosphamide | IV | J8530, J9070 |
| Cytarabine | IV | J9100 |
| Cytarabine Liposome | IV | J9098, C9024 |
| Dacarbazine | IV | J9130 |
| Dactinomycin | IV | J9120 |
| Daratumumab | IV | C9476, J9145 |
| Darbepoetin Alfa | IV | J0881, J0882 |
| Daunorubicin Hydrochloride | IV | J9150, J9151 |
| Daunorubicin Hydrochloride and Cytarabine Liposome | IV | C9024 |
| Decitabine | IV | J0894 |
| Degarelix | IV | J9155 |
| Denileukin Diftitox | IV | J9160 |
| Denosumab | IV | J0897 |
| Dexamethasone | IV | J1094, J1100, J7312, J7637, J7638, J8540 |
| Dexrazoxane Hydrochloride | IV | J1190 |
| Docetaxel | IV | J9171 |
| Doxorubicin Hydrochloride | IV | J9000 |
| Doxorubicin Hydrochloride Liposome | IV | Q2049, Q2050 |
| Durvalumab | Immunotherapy | C9492 |
| Elotuzumab | IV | C9477, C9176 |
| Epirubicin Hydrochloride | IV | J9178 |
| Epoetin Alfa | IV | J0885, J0886, Q4081, Q5105, Q5106 |
| Eribulin Mesylate | IV | J9179 |
| Etoposide | IV | J8560, J9181 |
| Everolimus | IV | J7527 |
| Filgrastim | IV | J1442, J1446, J1447, Q5101 |
| Fludarabine Phosphate | IV | J8562, J9185 |
| Fluorouracil Injection | IV | J9190 |
| Fulvestrant | IV | J9395 |
| Gefitinib | IV | J8565 |
| Gemcitabine Hydrochloride | IV | J9201 |
| Gemtuzumab Ozogamicin | IV | J9203, J9300 |
| Glucarpidase | IV | C9293 |
| Goserelin Acetate | IV | J9202 |
| Ibritumomab Tiuxetan | IV | A9542, A9543 |
| Idarubicin Hydrochloride | IV | J9211 |
| Ifosfamide | IV | J9208 |
| IL-2 (Aldesleukin) | IV | J9015 |
| Inotuzumab Ozogamicin | IV | C9028 |
| Ipilimumab | Immunotherapy | J9228 |
| Irinotecan Hydrochloride | IV | J9206 |
| Irinotecan Hydrochloride Liposome | IV | J9205, C9474 |
| Ixabepilone | IV | J9207 |
| Lanreotide Acetate | IV | J1930 |
| Leucovorin Calcium | IV | J0640 |
| Leuprolide Acetate | IV | J1950, J9217, J9218, J9219 |
| Mechlorethamine Hydrochloride | IV | J9230 |
| Melphalan | IV | J8600 |
| Melphalan Hydrochloride | IV | J9245 |
| Mesna | IV | J9209 |
| Methylnaltrexone Bromide | IV | J2212 |
| Mitomycin C | IV | J7315, J9280 |
| Mitoxantrone Hydrochloride | IV | J9293 |
| Necitumumab | IV | C9475, J9292 |
| Nelarabine | IV | J9261 |
| Nivolumab | Immunotherapy | C9453, J9299 |
| Obinutuzumab | IV | C9021, J9301 |
| Ofatumumab | Immunotherapy | J9302 |
| Olaratumab | IV | C9485, J9285 |
| Omacetaxine Mepesuccinate | IV | J9262 |
| Ondansetron Hydrochloride | IV | J2405, Q0162, S0119 |
| Oxaliplatin | IV | J9263 |
| Paclitaxel | IV | J9264, J9265, J9267, S1016 |
| Palifermin | IV | J2425 |
| Palonosetron Hydrochloride | IV | J2469 |
| Pamidronate Disodium | IV | J2430 |
| Panitumumab | IV | J9303 |
| Pegaspargase | IV | J9266 |
| Pegfilgrastim | IV | J2505 |
| Peginterferon Alfa-2b | IV | S0148 |
| Pembrolizumab | Immunotherapy | C9027, J9271 |
| Pemetrexed Disodium | IV | J9305 |
| Pertuzumab | IV | J9306 |
| Plerixafor | IV | J2562 |
| Pralatrexate | IV | J9307 |
| Radium 223 Dichloride | IV | A9606 |
| Ramucirumab | IV | C9025, J9308 |
| Rasburicase | IV | J2783 |
| Recombinant Interferon Alfa-2b | IV | J9214 |
| Rituxan (Rituximab) | IV | J9310 |
| Rituxan Hycela (Rituximab and Hyaluronidase Human) | IV | C9467 |
| Romidepsin | IV | J9315 |
| Romiplostim | IV | J2796 |
| Siltuximab | IV | C9455, J2860 |
| Talimogene Laherparepvec | IV | C9472, J9325 |
| Temozolomide | IV | J8700, J9328 |
| Temsirolimus | IV | J9330 |
| Thiotepa | IV | J9340 |
| Tocilizumab | IV | J3262 |
| Topotecan Hydrochloride | IV | J8705, J9351 |
| Trabectedin | IV | C9480, J9352 |
| Trastuzumab | IV | J9354. J9355 |
| Valrubicin | IV | J9357 |
| Vinblastine Sulfate | IV | J9360 |
| Vincristine Sulfate | IV | J9370 |
| Vincristine Sulfate Liposome | IV | J9371 |
| Vinorelbine Tartrate | IV | J9390 |
| Ziv-Aflibercept | IV | J9400 |
| Zoledronic Acid | IV | J3489 |
| Abemaciclib | Oral |  |
| Abiraterone Acetate | Oral |  |
| Acalabrutinib | Oral |  |
| Ado-Trastuzumab Emtansine | Oral |  |
| Afatinib Dimaleate | Oral |  |
| Alectinib | Oral |  |
| Aminolevulinic Acid | Oral | J7308, J7345 |
| Anastrozole | Oral |  |
| Apalutamide | Oral |  |
| Aprepitant | Oral |  |
| Asparaginase Erwinia chrysanthemi | Oral |  |
| Axitinib | Oral |  |
| Bexarotene | Oral |  |
| Bicalutamide | Oral |  |
| Bleomycin | Oral | J9040 |
| Blinatumomab | Oral |  |
| Bortezomib | Oral |  |
| Bosutinib | Oral |  |
| Brigatinib | Oral |  |
| BuMel | Oral |  |
| Cabozantinib-S-Malate | Oral |  |
| Ceritinib | Oral |  |
| Chlorambucil | Oral | S0172 |
| Cobimetinib | Oral |  |
| Crizotinib | Oral |  |
| Dabrafenib | Oral |  |
| Dasatinib | Oral |  |
| Defibrotide Sodium | Oral |  |
| Dinutuximab | Oral |  |
| Eltrombopag Olamine | Oral |  |
| Enasidenib Mesylate | Oral |  |
| Enzalutamide | Oral |  |
| Erlotinib Hydrochloride | Oral |  |
| Exemestane | Oral |  |
| Fluorouracil--Topical | Oral |  |
| Flutamide | Oral |  |
| Fostamatinib Disodium | Oral |  |
| Hydroxyurea | Oral | S0176 |
| Ibrutinib | Oral |  |
| Idelalisib | Oral |  |
| Imatinib Mesylate | Oral | S0088 |
| Imiquimod | Oral |  |
| Ixazomib Citrate | Oral |  |
| Lapatinib Ditosylate | Oral |  |
| Lenalidomide | Oral |  |
| Lenvatinib Mesylate | Oral |  |
| Letrozole | Oral |  |
| Lomustine | Oral | S0178 |
| Megestrol Acetate | Oral | S0179 |
| Mercaptopurine | Oral | S0108 |
| Methotrexate | Oral | J8610, J9250, J9260 |
| Midostaurin | Oral |  |
| Neratinib Maleate | Oral |  |
| Netupitant and Palonosetron Hydrochloride | Oral | C9448, J8655, Q9978 |
| Nilotinib | Oral |  |
| Nilutamide | Oral |  |
| Olaparib | Oral |  |
| Osimertinib | Oral |  |
| Palbociclib | Oral |  |
| Palonosetron Hydrochloride and Netupitant | Oral | J8655, Q9978 |
| Panobinostat | Oral |  |
| Pazopanib Hydrochloride | Oral |  |
| Pomalidomide | Oral |  |
| Ponatinib Hydrochloride | Oral |  |
| Prednisone | Oral | J7506, J7512 |
| Procarbazine Hydrochloride | Oral | S0182 |
| Raloxifene Hydrochloride | Oral |  |
| Regorafenib | Oral |  |
| Ribociclib | Oral |  |
| Rolapitant Hydrochloride | Oral | C9464, J8670, Q9981 |
| Rucaparib Camsylate | Oral |  |
| Ruxolitinib Phosphate | Oral |  |
| Sonidegib | Oral |  |
| Sorafenib Tosylate | Oral |  |
| Sprycel (Dasatinib) | Oral |  |
| Stivarga (Regorafenib) | Oral |  |
| Sunitinib Malate | Oral |  |
| Sutent (Sunitinib Malate) | Oral |  |
| Tamoxifen Citrate | Oral | S0187 |
| Thalidomide | Oral |  |
| Thioguanine | Oral |  |
| Toremifene | Oral |  |
| Trametinib | Oral |  |
| Trifluridine and Tipiracil Hydrochloride | Oral |  |
| Vandetanib | Oral |  |
| Vemurafenib | Oral |  |
| Venetoclax | Oral |  |
| Vismodegib | Oral |  |
| Vorinostat | Oral |  |
| Uridine Triacetate | Granule |  |
| Sipuleucel-T | Infusion | Q2043 |
| Tisagenlecleucel | Infusion | Q2040 |
| Injection, Abarelix For Injectable Suspension, Per 10 Mg | Hormone Therapy | C9216, J9216 |
| Injection, Abarelix, 10 Mg | Hormone Therapy | J0128, S0165 |
| Anastrozole, Oral, 1 Mg | Hormone Therapy | S0170 |
| Injection Degarelix 1 Mg | Hormone Therapy | J9155 |
| Diethylstilbestrol Diphosphate 250 Mh Iv | Hormone Therapy | J9165 |
| Exemestane, 25 Mg | Hormone Therapy | S0156 |
| Gonadorelin Hydrochloride | Hormone Therapy | J1620 |
| Histrelin Implant | Hormone Therapy | J9226, J9225 |
| Injection, Histrelin Acetate, 10 Mcg | Hormone Therapy | Q2020, J1675 |
| Hormonal Anti-Neoplastic | Hormone Therapy | G0356 |
| Injection Lanreotide 1 Mg | Hormone Therapy | J1930, C9237 |
| Injection, Medroxyprogesterone Acetate, 50 Mg | Hormone Therapy | J1051, J1050 |
| Megestrol Acetate, Oral, 20mg | Hormone Therapy | S0179 |
| Nadrolone Phenpropionate | Hormone Therapy | J0340 |
| Nandrolone Decanoate | Hormone Therapy | J2320, J2321, J2322 |
| Octreotide Acetate | Hormone Therapy | J2354, J2352, J2353 |
| Progesterone | Hormone Therapy | J2675 |
| Tamoxifen Citrate, Oral, 10mg | Hormone Therapy | S0187 |
| Testosterone Cypionate | Hormone Therapy | J1070, J1080, J1090 |
| Testosterone Cypionate And Estradiol Cypion | Hormone Therapy | J1060 |
| Testosterone Enanthate | Hormone Therapy | J3120, J3130 |
| Testosterone Enanthate And Estradiol Valera | Hormone Therapy | J0900 |
| Testosterone Propionate | Hormone Therapy | J3150 |
| Testosterone Suspension | Hormone Therapy | J3140 |
| Injection, Triptorelin Pamoate, 3.75 Mg | Hormone Therapy | J3315 |

| **List of chemotherapy administration codes**[[1](#_ENREF_1), [2](#_ENREF_2)] | |
| --- | --- |
| CPT Codes | 36640, 61517, 96401, 96402, 96405, 96406, 96409, 96411, 96413, 96415, 96416, 96417, 96420, 96422, 96423, 96425, 96440, 96445, 96446, 96450, 96542, 96549, 99555, Q0083, Q0084, Q0085, S9329, S9330, S9331, 4180F, 36640, 96410, 96408, 96400, 219583, 96542, 203682, 96414, 96412, 242226, 61517, 219687, 0519F, 96545, 206820, 206929, 96549, 99601, 99602, 62360, 62361, 62362, 36823, 36260, 61215, 90765, 90766, 90767, 90768, 96365, 96366, 96367, 96368, 96523, 90781, 90780, 96530, 96520, 95990, 95991, 96522, 96521, 62365, 0169T, 90775, 90776, 90773, 90774, 90772, 90783, 90784, 90782, 90799, 90779, 96379, 81350, 96912, 96910, 96570, 96571, 96567, 83520 |
| ICD-10-PCS | 3E06003, 3E06003, 3E06303, 3E06303, 3E04003, 3E04003, 3E04303, 3E04303, 3E0S303, 3E0S303, 3E05003, 3E05003, 3E05303, 3E05303, 3E03003, 3E03003, 3E03303, 3E03303, 3E0R303, 3E0R303, 3E0J30M, 3E0J30M, 3E0J70M, 3E0J70M, 3E0J80M, 3E0J80M, 3E0A30M, 3E0A30M, 3E0V30M, 3E0V30M, 3E0600M, 3E0600M, 3E0630M, 3E0630M, 3E0400M, 3E0400M, 3E0430M, 3E0430M, 3E0Q30M, 3E0Q30M, 3E0Q70M, 3E0Q70M, 3E0BX0M, 3E0BX0M, 3E0B30M, 3E0B30M, 3E0B70M, 3E0B70M, 3E0S30M, 3E0S30M, 3E0CX0M, 3E0CX0M, 3E0C30M, 3E0C30M, 3E0C70M, 3E0C70M, 3E0P30M, 3E0P30M, 3E0P70M, 3E0P70M, 3E0P80M, 3E0P80M, 3E0K30M, 3E0K30M, 3E0K70M, 3E0K70M, 3E0K80M, 3E0K80M, 3E0U30M, 3E0U30M, 3E0H30M, 3E0H30M, 3E0H70M, 3E0H70M, 3E0H80M, 3E0H80M, 3E0W30M, 3E0W30M, 3E0N30M, 3E0N30M, 3E0N70M, 3E0N70M, 3E0N80M, 3E0N80M, 3E0DX0M, 3E0DX0M, 3E0D30M, 3E0D30M, 3E0D70M, 3E0D70M, 3E0230M, 3E0230M, 3E09X0M, 3E09X0M, 3E0930M, 3E0930M, 3E0970M, 3E0970M, 3E0Y30M, 3E0Y30M, 3E0Y70M, 3E0Y70M, 3E0500M, 3E0500M, 3E0530M, 3E0530M, 3E0300M, 3E0300M, 3E0330M, 3E0330M, 3E0M30M, 3E0M30M, 3E0M70M, 3E0M70M, 3E0L30M, 3E0L30M, 3E0L70M, 3E0L70M, 3E0E30M, 3E0E30M, 3E0E70M, 3E0E70M, 3E0E80M, 3E0E80M, 3E0F30M, 3E0F30M, 3E0F70M, 3E0F70M, 3E0F80M, 3E0F80M, 3E00X0M, 3E00X0M, 3E0R30M, 3E0R30M, 3E0130M, 3E0130M, 3E0G30M, 3E0G30M, 3E0G70M, 3E0G70M, 3E0G80M, 3E0G80M, 3E06005, 3E06305, 3E04005, 3E04305, 3E0BX05, 3E0B305, 3E0B705, 3E0S305, 3E0CX05, 3E0C305, 3E0C705, 3E0U305, 3E0DX05, 3E0D305, 3E0D705, 3E09X05, 3E09305, 3E09705, 3E05005, 3E05305, 3E03005, 3E03305, 3E0R305, 3E0600P, 3E0630P, 3E0400P, 3E0430P, 3E0500P, 3E0530P, 3E0300P, 3E0330P, XW04351, XW03351 |
| **List of Radiation Therapy Codes[**[**1**](#_ENREF_1)**,** [**2**](#_ENREF_2)**]** | |
| ICD-10- Diagnosis | Z51.0, Z51.89, Z08, Z09, Y63.2, Y84.2 |
| CPT Codes | 77280, 77285, 77290, 77295, 77299, 77300, 77301, 77305, 77310, 77315, 77321, 77326, 77327, 77328, 77331, 77332, 77333, 77334, 77336, 77338, 77371, 77372, 77373, 77399, 77401, 77402, 77403, 77404, 77406, 77407, 77408, 77409, 77411, 77412, 77413, 77414, 77416, 77417, 77418, 77421, 77422, 77423, 77424, 77425, 77427, 77431, 77432, 77435, 77469, 77470, 77499, 77520, 77522, 77523, 77525, 77750, 77761, 77762, 77763, 77776, 77777, 77778, 77785, 77786, 77787, 77789, 77799, 0073T, 0082T, 0083T, 0182T, 0190T, 0197T, 19296, 19297, 19298, 20555, 20660, 31463, 32553, 41019, 49411, 49412, 52250, 55859, 55860, 55875, 55876, 55920, 57155, 57156, 58346, 61720, 61735, 61770, 61781, 61782, 61783, 61793, 61795, 61796, 61797, 61798, 61799, 61800, 63620, 63621, 73670, 76950, 76965, 77014, 77261, 77262, 77263, , 77285, 77306, 77307, 77370, 77380, 77381, 77385, 77386, 77387, 77405, 77410, 77415, 77419, 77420, 77430, 77781, 77782, 77783, 77784, ,77790, 79005, 79030, 79035, 79100, 79101, 79200, 79300, 79400, 79403, 79420, 79440, 79445, 79900, 79999 |
| ICD-10 Procedure Codes | 3E0B304, 3E0B704, 3E0BX04, 3E0C304, 3E0C704, 3E0CX04, 3E0D304, 3E0D704, 3E0DX04, 3E0E304, 3E0E704, 3E0E804, 3E0F304, 3E0F704, 3E0F804, 3E0G304, 3E0G704, 3E0G804, 3E0H304, 3E0H704, 3E0H804, 3E0J304, 3E0J704, 3E0J804, 3E0K304, 3E0K704, 3E0K804, 3E0L304, 3E0L704, 3E0M304, 3E0M704, 3E0N304, 3E0N704, 3E0N804, 3E0P304, 3E0P704, 3E0P804, 3E0Q304, 3E0Q704, 3E0R304, 3E0S304, 3E0U304, 3E0Y304, 3E0Y704, D0Y07ZZ, D0Y17ZZ, D0Y67ZZ, D0Y77ZZ, D8Y07ZZ, D9Y07ZZ, D9Y17ZZ, D9Y37ZZ, D9Y47ZZ, D9Y57ZZ, D9Y67ZZ, D9Y77ZZ, D9Y87ZZ, D9Y97ZZ, D9YB7ZZ, D9YD7ZZ, D9YF7ZZ, DBY07ZZ, DBY17ZZ, DBY27ZZ, DBY57ZZ, DBY67ZZ, DBY77ZZ, DBY87ZZ, DDY07ZZ, DDY17ZZ, DDY27ZZ, DDY37ZZ, DDY47ZZ, DDY57ZZ, DDY77ZZ, DFY07ZZ, DFY17ZZ, DFY27ZZ, DFY37ZZ, DGY07ZZ, DGY17ZZ, DGY27ZZ, DGY47ZZ, DGY57ZZ, DHY27ZZ, DHY37ZZ, DHY47ZZ, DHY67ZZ, DHY77ZZ, DHY87ZZ, DHY97ZZ, DHYB7ZZ, DMY07ZZ, DMY17ZZ, DPY07ZZ, DPY27ZZ, DPY37ZZ, DPY47ZZ, DPY57ZZ, DPY67ZZ, DPY77ZZ, DPY87ZZ, DPY97ZZ, DPYB7ZZ, DPYC7ZZ, DTY07ZZ, DTY17ZZ, DTY27ZZ, DTY37ZZ, DUY07ZZ, DUY17ZZ, DUY27ZZ, DVY07ZZ, DVY17ZZ, DWY17ZZ, DWY27ZZ, DWY37ZZ, DWY47ZZ, DWY57ZZ, DWY67ZZ, D0000ZZ, D0010ZZ, D0060ZZ, D0070ZZ, D7000ZZ, D7010ZZ, D7020ZZ, D7030ZZ, D7040ZZ, D7050ZZ, D7060ZZ, D7070ZZ, D7080ZZ, D8000ZZ, D9000ZZ, D9010ZZ, D9030ZZ, D9040ZZ, D9050ZZ, D9060ZZ, D9070ZZ, D9080ZZ, D9090ZZ, D90B0ZZ, D90D0ZZ, D90F0ZZ, DB000ZZ, DB010ZZ, DB020ZZ, DB050ZZ, DB060ZZ, DB070ZZ, DB080ZZ, DD000ZZ, DD010ZZ, DD020ZZ, DD030ZZ, DD040ZZ, DD050ZZ, DD070ZZ, DF000ZZ, DF010ZZ, DF020ZZ, DF030ZZ, DG000ZZ, DG010ZZ, DG020ZZ, DG040ZZ, DG050ZZ, DH020ZZ, DH030ZZ, DH040ZZ, DH060ZZ, DH070ZZ, DH080ZZ, DH090ZZ, DH0B0ZZ, DM000ZZ, DM010ZZ, DP000ZZ, DP020ZZ, DP030ZZ, DP040ZZ, DP050ZZ, DP060ZZ, DP070ZZ, DP080ZZ, DP090ZZ, DP0B0ZZ, DP0C0ZZ, DT000ZZ, DT010ZZ, DT020ZZ, DT030ZZ, DU000ZZ, DU010ZZ, DU020ZZ, DV000ZZ, DV010ZZ, DW010ZZ, DW020ZZ, DW030ZZ, DW040ZZ, DW050ZZ, DW060ZZ, D01097Z, D01098Z, D01099Z, D0109BZ, D0109CZ, D0109YZ, D010B7Z, D010B8Z, D010B9Z, D010BBZ, D010BCZ, D010BYZ, D01197Z, D01198Z, D01199Z, D0119BZ, D0119CZ, D0119YZ, D011B7Z, D011B8Z, D011B9Z, D011BBZ, D011BCZ, D011BYZ, D01697Z, D01698Z, D01699Z, D0169BZ, D0169CZ, D0169YZ, D016B7Z, D016B8Z, D016B9Z, D016BBZ, D016BCZ, D016BYZ, D01797Z, D01798Z, D01799Z, D0179BZ, D0179CZ, D0179YZ, D017B7Z, D017B8Z, D017B9Z, D017BBZ, D017BCZ, D017BYZ, D71097Z, D71098Z, D71099Z, D7109BZ, D7109CZ, D7109YZ, D710B7Z, D710B8Z, D710B9Z, D710BBZ, D710BCZ, D710BYZ, D71197Z, D71198Z, D71199Z, D7119BZ, D7119CZ, D7119YZ, D711B7Z, D711B8Z, D711B9Z, D711BBZ, D711BCZ, D711BYZ, D71297Z, D71298Z, D71299Z, D7129BZ, D7129CZ, D7129YZ, D712B7Z, D712B8Z, D712B9Z, D712BBZ, D712BCZ, D712BYZ, D71397Z, D71398Z, D71399Z, D7139BZ, D7139CZ, D7139YZ, D713B7Z, D713B8Z, D713B9Z, D713BBZ, D713BCZ, D713BYZ, D71497Z, D71498Z, D71499Z, D7149BZ, D7149CZ, D7149YZ, D714B7Z, D714B8Z, D714B9Z, D714BBZ, D714BCZ, D714BYZ, D71597Z, D71598Z, D71599Z, D7159BZ, D7159CZ, D7159YZ, D715B7Z, D715B8Z, D715B9Z, D715BBZ, D715BCZ, D715BYZ, D71697Z, D71698Z, D71699Z, D7169BZ, D7169CZ, D7169YZ, D716B7Z, D716B8Z, D716B9Z, D716BBZ, D716BCZ, D716BYZ, D71797Z, D71798Z, D71799Z, D7179BZ, D7179CZ, D7179YZ, D717B7Z, D717B8Z, D717B9Z, D717BBZ, D717BCZ, D717BYZ, D71897Z, D71898Z, D71899Z, D7189BZ, D7189CZ, D7189YZ, D718B7Z, D718B8Z, D718B9Z, D718BBZ, D718BCZ, D718BYZ, D81097Z, D81098Z, D81099Z, D8109BZ, D8109CZ, D8109YZ, D810B7Z, D810B8Z, D810B9Z, D810BBZ, D810BCZ, D810BYZ, D91097Z, D91098Z, D91099Z, D9109BZ, D9109CZ, D9109YZ, D910B7Z, D910B8Z, D910B9Z, D910BBZ, D910BCZ, D910BYZ, D91197Z, D91198Z, D91199Z, D9119BZ, D9119CZ, D9119YZ, D911B7Z, D911B8Z, D911B9Z, D911BBZ, D911BCZ, D911BYZ, D91397Z, D91398Z, D91399Z, D9139BZ, D9139CZ, D9139YZ, D913B7Z, D913B8Z, D913B9Z, D913BBZ, D913BCZ, D913BYZ, D91497Z, D91498Z, D91499Z, D9149BZ, D9149CZ, D9149YZ, D914B7Z, D914B8Z, D914B9Z, D914BBZ, D914BCZ, D914BYZ, D91597Z, D91598Z, D91599Z, D9159BZ, D9159CZ, D9159YZ, D915B7Z, D915B8Z, D915B9Z, D915BBZ, D915BCZ, D915BYZ, D91697Z, D91698Z, D91699Z, D9169BZ, D9169CZ, D9169YZ, D916B7Z, D916B8Z, D916B9Z, D916BBZ, D916BCZ, D916BYZ, D91797Z, D91798Z, D91799Z, D9179BZ, D9179CZ, D9179YZ, D917B7Z, D917B8Z, D917B9Z, D917BBZ, D917BCZ, D917BYZ, D91897Z, D91898Z, D91899Z, D9189BZ, D9189CZ, D9189YZ, D918B7Z, D918B8Z, D918B9Z, D918BBZ, D918BCZ, D918BYZ, D91997Z, D91998Z, D91999Z, D9199BZ, D9199CZ, D9199YZ, D919B7Z, D919B8Z, D919B9Z, D919BBZ, D919BCZ, D919BYZ, D91B97Z, D91B98Z, D91B99Z, D91B9BZ, D91B9CZ, D91B9YZ, D91BB7Z, D91BB8Z, D91BB9Z, D91BBBZ, D91BBCZ, D91BBYZ, D91D97Z, D91D98Z, D91D99Z, D91D9BZ, D91D9CZ, D91D9YZ, D91DB7Z, D91DB8Z, D91DB9Z, D91DBBZ, D91DBCZ, D91DBYZ, D91F97Z, D91F98Z, D91F99Z, D91F9BZ, D91F9CZ, D91F9YZ, D91FB7Z, D91FB8Z, D91FB9Z, D91FBBZ, D91FBCZ, D91FBYZ, DB1097Z, DB1098Z, DB1099Z, DB109BZ, DB109CZ, DB109YZ, DB10B7Z, DB10B8Z, DB10B9Z, DB10BBZ, DB10BCZ, DB10BYZ, DB1197Z, DB1198Z, DB1199Z, DB119BZ, DB119CZ, DB119YZ, DB11B7Z, DB11B8Z, DB11B9Z, DB11BBZ, DB11BCZ, DB11BYZ, DB1297Z, DB1298Z, DB1299Z, DB129BZ, DB129CZ, DB129YZ, DB12B7Z, DB12B8Z, DB12B9Z, DB12BBZ, DB12BCZ, DB12BYZ, DB1597Z, DB1598Z, DB1599Z, DB159BZ, DB159CZ, DB159YZ, DB15B7Z, DB15B8Z, DB15B9Z, DB15BBZ, DB15BCZ, DB15BYZ, DB1697Z, DB1698Z, DB1699Z, DB169BZ, DB169CZ, DB169YZ, DB16B7Z, DB16B8Z, DB16B9Z, DB16BBZ, DB16BCZ, DB16BYZ, DB1797Z,  DB1798Z, DB1799Z, DB179BZ, DB179CZ, DB179YZ, DB17B7Z, DB17B8Z, DB17B9Z, DB17BBZ, DB17BCZ, DB17BYZ, DB1897Z, DB1898Z, DB1899Z, DB189BZ, DB189CZ, DB189YZ, DB18B7Z, DB18B8Z, DB18B9Z, DB18BBZ, DB18BCZ, DB18BYZ, DD1097Z, DD1098Z, DD1099Z, DD109BZ, DD109CZ, DD109YZ, DD10B7Z, DD10B8Z, DD10B9Z, DD10BBZ, DD10BCZ, DD10BYZ, DD1197Z, DD1198Z, DD1199Z, DD119BZ, DD119CZ, DD119YZ, DD11B7Z, DD11B8Z, DD11B9Z, DD11BBZ, DD11BCZ, DD11BYZ, DD1297Z, DD1298Z, DD1299Z, DD129BZ, DD129CZ, DD129YZ, DD12B7Z, DD12B8Z, DD12B9Z, DD12BBZ, DD12BCZ, DD12BYZ, DD1397Z, DD1398Z, DD1399Z, DD139BZ, DD139CZ, DD139YZ, DD13B7Z, DD13B8Z, DD13B9Z, DD13BBZ, DD13BCZ, DD13BYZ, DD1497Z, DD1498Z, DD1499Z, DD149BZ, DD149CZ, DD149YZ, DD14B7Z, DD14B8Z, DD14B9Z, DD14BBZ, DD14BCZ, DD14BYZ, DD1597Z, DD1598Z, DD1599Z, DD159BZ, DD159CZ, DD159YZ, DD15B7Z, DD15B8Z, DD15B9Z, DD15BBZ, DD15BCZ, DD15BYZ, DD1797Z, DD1798Z, DD1799Z, DD179BZ, DD179CZ, DD179YZ, DD17B7Z, DD17B8Z, DD17B9Z, DD17BBZ, DD17BCZ, DD17BYZ, DF1097Z, DF1098Z, DF1099Z, DF109BZ, DF109CZ, DF109YZ, DF10B7Z, DF10B8Z, DF10B9Z, DF10BBZ, DF10BCZ, DF10BYZ, DF1197Z, DF1198Z, DF1199Z, DF119BZ, DF119CZ, DF119YZ, DF11B7Z, DF11B8Z, DF11B9Z, DF11BBZ, DF11BCZ, DF11BYZ, DF1297Z, DF1298Z, DF1299Z, DF129BZ, DF129CZ, DF129YZ, DF12B7Z, DF12B8Z, DF12B9Z, DF12BBZ, DF12BCZ, DF12BYZ, DF1397Z, DF1398Z, DF1399Z, DF139BZ, DF139CZ, DF139YZ, DF13B7Z, DF13B8Z, DF13B9Z, DF13BBZ, DF13BCZ, DF13BYZ, DG1097Z, DG1098Z, DG1099Z, DG109BZ, DG109CZ, DG109YZ, DG10B7Z, DG10B8Z, DG10B9Z, DG10BBZ, DG10BCZ, DG10BYZ, DG1197Z, DG1198Z, DG1199Z, DG119BZ, DG119CZ, DG119YZ, DG11B7Z, DG11B8Z, DG11B9Z, DG11BBZ, DG11BCZ, DG11BYZ, DG1297Z, DG1298Z, DG1299Z, DG129BZ, DG129CZ, DG129YZ, DG12B7Z, DG12B8Z, DG12B9Z, DG12BBZ, DG12BCZ, DG12BYZ, DG1497Z, DG1498Z, DG1499Z, DG149BZ, DG149CZ, DG149YZ, DG14B7Z, DG14B8Z, DG14B9Z, DG14BBZ, DG14BCZ, DG14BYZ, DG1597Z, DG1598Z, DG1599Z, DG159BZ, DG159CZ, DG159YZ, DG15B7Z, DG15B8Z, DG15B9Z, DG15BBZ, DG15BCZ, DG15BYZ, DM1097Z, DM1098Z, DM1099Z, DM109BZ, DM109CZ, DM109YZ, DM10B7Z, DM10B8Z, DM10B9Z, DM10BBZ, DM10BCZ, DM10BYZ, DM1197Z, DM1198Z, DM1199Z, DM119BZ, DM119CZ, DM119YZ, DM11B7Z, DM11B8Z, DM11B9Z, DM11BBZ, DM11BCZ, DM11BYZ, DT1097Z, DT1098Z, DT1099Z, DT109BZ, DT109CZ, DT109YZ, DT10B7Z, DT10B8Z, DT10B9Z, DT10BBZ, DT10BCZ, DT10BYZ, DT1197Z, DT1198Z, DT1199Z, DT119BZ, DT119CZ, DT119YZ, DT11B7Z, DT11B8Z, DT11B9Z, DT11BBZ, DT11BCZ, DT11BYZ, DT1297Z, DT1298Z, DT1299Z, DT129BZ, DT129CZ, DT129YZ, DT12B7Z, DT12B8Z, DT12B9Z, DT12BBZ, DT12BCZ, DT12BYZ, DT1397Z, DT1398Z, DT1399Z, DT139BZ, DT139CZ, DT139YZ, DT13B7Z, DT13B8Z, DT13B9Z, DT13BBZ, DT13BCZ, DT13BYZ, DU1097Z, DU1098Z, DU1099Z, DU109BZ, DU109CZ, DU109YZ, DU10B7Z, DU10B8Z, DU10B9Z, DU10BBZ, DU10BCZ, DU10BYZ, DU1197Z, DU1198Z, DU1199Z, DU119BZ, DU119CZ, DU119YZ, DU11B7Z, DU11B8Z, DU11B9Z, DU11BBZ, DU11BCZ, DU11BYZ, DU1297Z, DU1298Z, DU1299Z, DU129BZ, DU129CZ, DU129YZ, DU12B7Z, DU12B8Z, DU12B9Z, DU12BBZ, DU12BCZ, DU12BYZ, DV1097Z, DV1098Z, DV1099Z, DV109BZ, DV109CZ, DV109YZ, DV10B7Z, DV10B8Z, DV10B9Z, DV10BBZ, DV10BCZ, DV10BYZ, DV1197Z, DV1198Z, DV1199Z, DV119BZ, DV119CZ, DV119YZ, DV11B7Z, DV11B8Z, DV11B9Z, DV11BBZ, DV11BCZ, DV11BYZ, DW1197Z, DW1198Z, DW1199Z, DW119BZ, DW119CZ, DW119YZ, DW11B7Z, DW11B8Z, DW11B9Z, DW11BBZ, DW11BCZ, DW11BYZ, DW1297Z, DW1298Z, DW1299Z, DW129BZ, DW129CZ, DW129YZ, DW12B7Z, DW12B8Z, DW12B9Z, DW12BBZ, DW12BCZ, DW12BYZ, DW1397Z, DW1398Z, DW1399Z, DW139BZ, DW139CZ, DW139YZ, DW13B7Z, DW13B8Z, DW13B9Z, DW13BBZ, DW13BCZ, DW13BYZ, DW1697Z, DW1698Z, DW1699Z, DW169BZ, DW169CZ, DW169YZ, DW16B7Z, DW16B8Z, DW16B9Z, DW16BBZ, DW16BCZ, DW16BYZ, D0000ZZ, D0001ZZ, D0002ZZ, D0010ZZ, D0011ZZ, D0012ZZ, D0060ZZ, D0061ZZ, D0062ZZ, D0070ZZ, D0071ZZ, D0072ZZ, D7000ZZ, D7001ZZ, D7002ZZ, D7010ZZ, D7011ZZ, D7012ZZ, D7020ZZ, D7021ZZ, D7022ZZ, D7030ZZ, D7031ZZ, D7032ZZ, D7040ZZ, D7041ZZ, D7042ZZ, D7050ZZ, D7051ZZ, D7052ZZ, D7060ZZ, D7061ZZ, D7062ZZ, D7070ZZ, D7071ZZ, D7072ZZ, D7080ZZ, D7081ZZ, D7082ZZ, D8000ZZ, D8001ZZ, D8002ZZ, D9000ZZ, D9001ZZ, D9002ZZ, D9010ZZ, D9011ZZ, D9012ZZ, D9030ZZ, D9031ZZ, D9032ZZ, D9040ZZ, D9041ZZ, D9042ZZ, D9050ZZ, D9051ZZ, D9052ZZ, D9060ZZ, D9061ZZ, D9062ZZ, D9070ZZ, D9071ZZ, D9072ZZ, D9080ZZ, D9081ZZ, D9082ZZ, D9090ZZ, D9091ZZ, D9092ZZ, D90B0ZZ, D90B1ZZ, D90B2ZZ, D90D0ZZ, D90D1ZZ, D90D2ZZ, D90F0ZZ, D90F1ZZ, D90F2ZZ, DB000ZZ, DB001ZZ, DB002ZZ, DB010ZZ, DB011ZZ, DB012ZZ, DB020ZZ, DB021ZZ, DB022ZZ, DB050ZZ, DB051ZZ, DB052ZZ, DB060ZZ, DB061ZZ, DB062ZZ, DB070ZZ, DB071ZZ, DB072ZZ, DB080ZZ, DB081ZZ, DB082ZZ, DD000ZZ, DD001ZZ, DD002ZZ, DD010ZZ, DD011ZZ, DD012ZZ, DD020ZZ, DD021ZZ, DD022ZZ, DD030ZZ, DD031ZZ, DD032ZZ, DD040ZZ, DD041ZZ, DD042ZZ, DD050ZZ, DD051ZZ, DD052ZZ, DD070ZZ, DD071ZZ, DD072ZZ, DF000ZZ, DF001ZZ, DF002ZZ, DF010ZZ, DF011ZZ, DF012ZZ, DF020ZZ, DF021ZZ, DF022ZZ, DF030ZZ, DF031ZZ, DF032ZZ, DG000ZZ, DG001ZZ, DG002ZZ, DG010ZZ, DG011ZZ, DG012ZZ, DG020ZZ, DG021ZZ, DG022ZZ, DG040ZZ, DG041ZZ, DG042ZZ, DG050ZZ, DG051ZZ, DG052ZZ, DH020ZZ, DH021ZZ, DH022ZZ, DH030ZZ, DH031ZZ, DH032ZZ, DH040ZZ, DH041ZZ, DH042ZZ, DH060ZZ, DH061ZZ, DH062ZZ, DH070ZZ, DH071ZZ, DH072ZZ, DH080ZZ, DH081ZZ, DH082ZZ, DH090ZZ, DH091ZZ, DH092ZZ, DH0B0ZZ, DH0B1ZZ, DH0B2ZZ, DM000ZZ, DM001ZZ, DM002ZZ, DM010ZZ, DM011ZZ, DM012ZZ, DP000ZZ, DP001ZZ, DP002ZZ, DP020ZZ, DP021ZZ, DP022ZZ, DP030ZZ, DP031ZZ, DP032ZZ, DP040ZZ, DP041ZZ, DP042ZZ, DP050ZZ, DP051ZZ, DP052ZZ, DP060ZZ, DP061ZZ, DP062ZZ, DP070ZZ, DP071ZZ, DP072ZZ, DP080ZZ, DP081ZZ, DP082ZZ, DP090ZZ, DP091ZZ, DP092ZZ, DP0B0ZZ, DP0B1ZZ, DP0B2ZZ, DP0C0ZZ, DP0C1ZZ, DP0C2ZZ, DT000ZZ, DT001ZZ, DT002ZZ, DT010ZZ, DT011ZZ, DT012ZZ, DT020ZZ, DT021ZZ, DT022ZZ, DT030ZZ, DT031ZZ, DT032ZZ, DU000ZZ, DU001ZZ, DU002ZZ, DU010ZZ, DU011ZZ, DU012ZZ, DU020ZZ, DU021ZZ, DU022ZZ, DV000ZZ, DV001ZZ, DV002ZZ, DV010ZZ, DV011ZZ, DV012ZZ, DW010ZZ, DW011ZZ, DW012ZZ, DW020ZZ, DW021ZZ, DW022ZZ, DW030ZZ, DW031ZZ, DW032ZZ, DW040ZZ, DW041ZZ, DW042ZZ, DW050ZZ, DW051ZZ, DW052ZZ, DW060ZZ, DW061ZZ, DW062ZZ, D0003ZZ, D0013ZZ, D0063ZZ, D0073ZZ, D7003ZZ, D7013ZZ, D7023ZZ, D7033ZZ, D7043ZZ, D7053ZZ, D7063ZZ, D7073ZZ, D7083ZZ, D8003ZZ, D9003ZZ, D9013ZZ, D9033ZZ, D9043ZZ, D9053ZZ, D9063ZZ, D9073ZZ, D9083ZZ, D9093ZZ, D90B3ZZ, D90D3ZZ, D90F3ZZ, DB003ZZ, DB013ZZ, DB023ZZ, DB053ZZ, DB063ZZ, DB073ZZ, DB083ZZ, DD003ZZ, DD013ZZ, DD023ZZ, DD033ZZ, DD043ZZ, DD053ZZ, DD073ZZ, DF003ZZ, DF013ZZ, DF023ZZ, DF033ZZ, DG003ZZ, DG013ZZ, DG023ZZ, DG043ZZ, DG053ZZ, DH023ZZ, DH033ZZ, DH043ZZ, DH063ZZ, DH073ZZ, DH083ZZ, DH093ZZ, DH0B3ZZ, DM003ZZ, DM013ZZ, DP003ZZ, DP023ZZ, DP033ZZ, DP043ZZ, DP053ZZ, DP063ZZ, DP073ZZ, DP083ZZ, DP093ZZ, DP0B3ZZ, DP0C3ZZ, DT003ZZ, DT013ZZ, DT023ZZ, DT033ZZ, DU003ZZ, DU013ZZ, DU023ZZ, DV003ZZ, DV013ZZ, DW013ZZ, DW023ZZ, DW033ZZ, DW043ZZ, DW053ZZ, DW063ZZ, D0004ZZ, D0005ZZ, D0014ZZ, D0015ZZ, D0064ZZ, D0065ZZ, D0074ZZ, D0075ZZ, D7004ZZ, D7005ZZ, D7014ZZ, D7015ZZ, D7024ZZ, D7025ZZ, D7034ZZ, D7035ZZ, D7044ZZ, D7045ZZ, D7054ZZ, D7055ZZ, D7064ZZ, D7065ZZ, D7074ZZ, D7075ZZ, D7084ZZ, D7085ZZ, D8004ZZ, D8005ZZ, D9004ZZ, D9005ZZ, D9014ZZ, D9015ZZ, D9034ZZ, D9035ZZ, D9044ZZ, D9045ZZ, D9054ZZ, D9055ZZ, D9064ZZ, D9065ZZ, D9074ZZ, D9075ZZ, D9084ZZ, D9085ZZ, D9094ZZ, D9095ZZ, D90B4ZZ, D90B5ZZ, D90D4ZZ, D90D5ZZ, D90F4ZZ, D90F5ZZ, DB004ZZ, DB005ZZ, DB014ZZ, DB015ZZ, DB024ZZ, DB025ZZ, DB054ZZ, DB055ZZ, DB064ZZ, DB065ZZ, DB074ZZ, DB075ZZ, DB084ZZ, DB085ZZ, DD004ZZ, DD005ZZ, DD014ZZ, DD015ZZ, DD024ZZ, DD025ZZ, DD034ZZ, DD035ZZ, DD044ZZ, DD045ZZ, DD054ZZ, DD055ZZ, DD074ZZ, DD075ZZ, DF004ZZ, DF005ZZ, DF014ZZ, DF015ZZ, DF024ZZ, DF025ZZ, DF034ZZ, DF035ZZ, DG005ZZ, DG015ZZ, DG025ZZ, DG045ZZ, DG055ZZ, DH024ZZ, DH025ZZ, DH034ZZ, DH035ZZ, DH044ZZ, DH045ZZ, DH064ZZ, DH065ZZ, DH074ZZ, DH075ZZ, DH084ZZ, DH085ZZ, DH094ZZ, DH095ZZ, DH0B4ZZ, DH0B5ZZ, DM004ZZ, DM005ZZ, DM014ZZ, DM015ZZ, DP004ZZ, DP005ZZ, DP024ZZ, DP025ZZ, DP034ZZ, DP035ZZ, DP044ZZ, DP045ZZ, DP054ZZ, DP055ZZ, DP064ZZ, DP065ZZ, DP074ZZ, DP075ZZ, DP084ZZ, DP085ZZ, DP094ZZ, DP095ZZ, DP0B4ZZ, DP0B5ZZ, DP0C4ZZ, DP0C5ZZ, DT004ZZ, DT005ZZ, DT014ZZ, DT015ZZ, DT024ZZ, DT025ZZ, DT034ZZ, DT035ZZ, DU004ZZ, DU005ZZ, DU014ZZ, DU015ZZ, DU024ZZ, DU025ZZ, DV004ZZ, DV005ZZ, DV014ZZ, DV015ZZ, DW014ZZ, DW015ZZ, DW024ZZ, DW025ZZ, DW034ZZ, DW035ZZ, DW044ZZ, DW045ZZ, DW054ZZ, DW055ZZ, DW064ZZ, DW065ZZ, 08H031Z, 08H0X1Z, 08H131Z, 08H1X1Z, 0BH001Z, 0BH031Z, 0BH041Z, 0BH071Z, 0BH081Z, 0BHK01Z, 0BHK31Z, 0BHK41Z, 0BHK71Z, 0BHK81Z, 0BHL01Z, 0BHL31Z, 0BHL41Z, 0BHL71Z, 0BHL81Z, 0CH701Z, 0CH731Z, 0CH7X1Z, 0DH501Z, 0DH531Z, 0DH541Z, 0DH571Z, 0DH581Z, 0DHP01Z, 0DHP31Z, 0DHP41Z, 0DHP71Z, 0DHP81Z, 0FHB01Z, 0FHB31Z, 0FHB41Z, 0FHB71Z, 0FHB81Z, 0FHD01Z, 0FHD31Z, 0FHD41Z, 0FHD71Z, 0FHD81Z, 0HHT01Z, 0HHT31Z, 0HHT71Z, 0HHT81Z, 0HHTX1Z, 0HHU01Z, 0HHU31Z, 0HHU71Z, 0HHU81Z, 0HHUX1Z, 0HHV01Z, 0HHV31Z, 0HHV71Z, 0HHV81Z, 0HHVX1Z, 0HHW01Z, 0HHW31Z, 0HHW71Z, 0HHW81Z, 0HHWX1Z, 0HHX01Z, 0HHX31Z, 0HHX71Z, 0HHX81Z, 0HHXX1Z, 0JHS01Z, 0JHS31Z, 0JHT01Z, 0JHT31Z, 0JHV01Z, 0JHV31Z, 0JHW01Z, 0JHW31Z, 0UHC01Z, 0UHC31Z, 0UHC41Z, 0UHC71Z, 0UHC81Z, 0UHG01Z, 0UHG31Z, 0UHG41Z, 0UHG71Z, 0UHG81Z, 0UHGX1Z, 0VH001Z, 0VH031Z, 0VH041Z, 0VH071Z, 0VH081Z, 0WH001Z, 0WH031Z, 0WH041Z, 0WH101Z, 0WH131Z, 0WH141Z, 0WH201Z, 0WH231Z, 0WH241Z, 0WH301Z, 0WH331Z, 0WH341Z, 0WH401Z, 0WH431Z, 0WH441Z, 0WH501Z, 0WH531Z, 0WH541Z, 0WH601Z, 0WH631Z, 0WH641Z, 0WH801Z, 0WH831Z, 0WH841Z, 0WH901Z, 0WH931Z, 0WH941Z, 0WHB01Z, 0WHB31Z, 0WHB41Z, 0WHC01Z, 0WHC31Z, 0WHC41Z, 0WHD01Z, 0WHD31Z, 0WHD41Z, 0WHF01Z, 0WHF31Z, 0WHF41Z, 0WHG01Z, 0WHG31Z, 0WHG41Z, 0WHH01Z, 0WHH31Z, 0WHH41Z, 0WHJ01Z, 0WHJ31Z, 0WHJ41Z, 0WHK01Z, 0WHK31Z, 0WHK41Z, 0WHL01Z, 0WHL31Z, 0WHL41Z, 0WHM01Z, 0WHM31Z, 0WHM41Z, 0WHN01Z, 0WHN31Z, 0WHN41Z, 0WHP01Z, 0WHP31Z, 0WHP41Z, 0WHP71Z, 0WHP81Z, 0WHQ01Z, 0WHQ31Z, 0WHQ41Z, 0WHQ71Z, 0WHQ81Z, 0WHR01Z, 0WHR31Z, 0WHR41Z, 0WHR71Z, 0WHR81Z, 0XH201Z, 0XH231Z, 0XH241Z, 0XH301Z, 0XH331Z, 0XH341Z, 0XH401Z, 0XH431Z, 0XH441Z, 0XH501Z, 0XH531Z, 0XH541Z, 0XH601Z, 0XH631Z, 0XH641Z, 0XH701Z, 0XH731Z, 0XH741Z, 0XH801Z, 0XH831Z, 0XH841Z, 0XH901Z, 0XH931Z, 0XH941Z, 0XHB01Z, 0XHB31Z, 0XHB41Z, 0XHC01Z, 0XHC31Z, 0XHC41Z, 0XHD01Z, 0XHD31Z, 0XHD41Z, 0XHF01Z, 0XHF31Z, 0XHF41Z, 0XHG01Z, 0XHG31Z, 0XHG41Z, 0XHH01Z, 0XHH31Z, 0XHH41Z, 0XHJ01Z, 0XHJ31Z, 0XHJ41Z, 0XHK01Z, 0XHK31Z, 0XHK41Z, 0YH001Z, 0YH031Z, 0YH041Z, 0YH101Z, 0YH131Z, 0YH141Z, 0YH501Z, 0YH531Z, 0YH541Z, 0YH601Z, 0YH631Z, 0YH641Z, 0YH701Z, 0YH731Z, 0YH741Z, 0YH801Z, 0YH831Z, 0YH841Z, 0YH901Z, 0YH931Z, 0YH941Z, 0YHB01Z, 0YHB31Z, 0YHB41Z, 0YHC01Z, 0YHC31Z, 0YHC41Z, 0YHD01Z, 0YHD31Z, 0YHD41Z, 0YHF01Z, 0YHF31Z, 0YHF41Z, 0YHG01Z, 0YHG31Z, 0YHG41Z, 0YHH01Z, 0YHH31Z, 0YHH41Z, 0YHJ01Z, 0YHJ31Z, 0YHJ41Z, 0YHK01Z, 0YHK31Z, 0YHK41Z, 0YHL01Z, 0YHL31Z, 0YHL41Z, 0YHM01Z, 0YHM31Z, 0YHM41Z, 0YHN01Z, 0YHN31Z, 0YHN41Z, CW70NZZ, CW70YZZ, CW73NZZ, CW73YZZ, CW7GGZZ, CW7GYZZ, CW7N8ZZ, CW7NGZZ, CW7NNZZ, CW7NPZZ, CW7NYZZ, CW7YYZZ, DWY5GDZ, DWY5GFZ, DWY5GGZ, DWY5GHZ, DWY5GYZ, D0Y0FZZ, D0Y1FZZ, D0Y6FZZ, D0Y7FZZ, D7Y0FZZ, D7Y1FZZ, D7Y2FZZ, D7Y3FZZ, D7Y4FZZ, D7Y5FZZ, D7Y6FZZ, D7Y7FZZ, D7Y8FZZ, D8Y0FZZ, D9Y0FZZ, D9Y1FZZ, D9Y4CZZ, D9Y4FZZ, D9Y5FZZ, D9Y6FZZ, D9Y7FZZ, D9Y8FZZ, D9Y9FZZ, D9YBCZZ, D9YBFZZ, D9YCCZZ, D9YCFZZ, D9YDCZZ, D9YDFZZ, DBY0FZZ, DBY1FZZ, DBY2FZZ, DBY5FZZ, DBY6FZZ, DBY7FZZ, DBY8FZZ, DDY0FZZ, DDY1CZZ, DDY1FZZ, DDY2CZZ, DDY2FZZ, DDY3CZZ, DDY3FZZ, DDY4CZZ, DDY4FZZ, DDY5CZZ, DDY5FZZ, DDY7CZZ, DDY7FZZ, DDY8CZZ, DDY8FZZ, DFY0CZZ, DFY0FZZ, DFY1CZZ, DFY1FZZ, DFY2CZZ, DFY2FZZ, DFY3CZZ, DFY3FZZ, DGY0FZZ, DGY1FZZ, DGY2FZZ, DGY4FZZ, DGY5FZZ, DHY2FZZ, DHY3FZZ, DHY4FZZ, DHY5FZZ, DHY6FZZ, DHY7FZZ, DHY8FZZ, DHY9FZZ, DHYBFZZ, DHYCFZZ, DMY0FZZ, DMY1FZZ, DPY0FZZ, DPY2FZZ, DPY3FZZ, DPY4FZZ, DPY5FZZ, DPY6FZZ, DPY7FZZ, DPY8FZZ, DPY9FZZ, DPYBFZZ, DPYCFZZ, DTY0CZZ, DTY0FZZ, DTY1CZZ, DTY1FZZ, DTY2CZZ, DTY2FZZ, DTY3CZZ, DTY3FZZ, DUY0CZZ, DUY0FZZ, DUY1CZZ, DUY1FZZ, DUY2CZZ, DUY2FZZ, DVY0CZZ, DVY0FZZ, DVY1FZZ, DWY1FZZ, DWY2FZZ, DWY3FZZ, DWY4FZZ, DWY5FZZ, DWY6FZZ, D0003Z0, D0013Z0, D0063Z0, D0073Z0, D7003Z0, D7013Z0, D7023Z0, D7033Z0, D7043Z0, D7053Z0, D7063Z0, D7073Z0, D7083Z0, D8003Z0, D9003Z0, D9013Z0, D9033Z0, D9043Z0, D9053Z0, D9063Z0, D9073Z0, D9083Z0, D9093Z0, D90B3Z0, D90D3Z0, D90F3Z0, DB003Z0, DB013Z0, DB023Z0, DB053Z0, DB063Z0, DB073Z0, DB083Z0, DD003Z0, DD013Z0, DD023Z0, DD033Z0, DD043Z0, DD053Z0, DD073Z0, DF003Z0, DF013Z0, DF023Z0, DF033Z0, DG003Z0, DG013Z0, DG023Z0, DG043Z0, DG053Z0, DH023Z0, DH033Z0, DH043Z0, DH063Z0, DH073Z0, DH083Z0, DH093Z0, DH0B3Z0, DM003Z0, DM013Z0, DP003Z0, DP023Z0, DP033Z0, DP043Z0, DP053Z0, DP063Z0, DP073Z0, DP083Z0, DP093Z0, DP0B3Z0, DP0C3Z0, DT003Z0, DT013Z0, DT023Z0, DT033Z0, DU003Z0, DU013Z0, DU023Z0, DV003Z0, DV013Z0, DW013Z0, DW023Z0, DW033Z0, DW043Z0, DW053Z0, DW063Z0, |
| **HCPCS** | A4650, A9606, A9699, C1715 , C1716 , C1717 , C1718 , C1719 , C1720 , C1728 , C2616 , C2633 , C2634 , C2635 , C2636 , C2637 , C2638 , C2639 , C2640 , C2641 , C2642 , C2643 , C2698 , C2699 ,C9726, C9728, G0173, G0174, G0242, G0243, G0251, G0338, G0339, G0340, G6003, G6004, G6005, G6006, G6007, G6008, G6009, G6010, G6011, G6012, G6013, G6014, G6015, G6016, Q3001 , S2270, S8049, C1325, C1348, C1350, C1700 , C1701, C1702, C1703, C1704, C1705, C1706, C1707, C1708, C1709, C1710, C1711, C1712, C1790 , C1791, C1792, C1793, C1794, C1795, C1796, C1797, C1798, C1799, C1800, C1801, C1802, C1803, C1804, C1805, C1806, C2632, C9714, C9715, G0178, G0256, G0273, G0274, G0338, G0339, G0340, G0458, C2644, C2645 |

CPT: Current Procedural Terminology; HCPCS: Healthcare Common Procedure Coding System; ICD-10-CM: International Classification of Diseases, 10th Revision, Clinical Modification; ICD-10-PCS: International Classification of Diseases, 10th Revision, Procedure Coding System; IV: intravenous; NDC: National Drug Code.

Table G. List of baseline covariates

| **Conditions** | **Type of Codes** | **Codes** |
| --- | --- | --- |
| Acquired immunodeficiency syndrome (AIDS)[[1](#_ENREF_1), [2](#_ENREF_2)] | ICD-10-CM diagnosis | B20, Z21 |
| Alcohol abuse [[1](#_ENREF_1), [2](#_ENREF_2)] | CPT | 99408, 99409, G0396, G0397, H0049, H0050 |
|  | ICD-10-CM diagnosis | G621, I426, K700, K702, K709, R780, E8600, E8601, F1026, F1096, F1097, F1027, F1014, F1024, F1019, F1029, F1094, F1099, F1020, F1021, F1010, K2920, K2921, K7010, K7011, K7030, K7031, K7040, F10121, F10221, F10231, F10921, F10151, F10251, F10951, F10920, F10929, F10150, F10250, F10950, F10230, F10232, F10239, F10182, F10282, F10982, F10159, F10180, F10181, F10188, F10259, F10280, F10281, F10288, F10959, F10980, F10981, F10988, F10220, F10229, F10120, F10129 |
|  | ICD-10-PCS Procedure | HZ2ZZZZ, HZ30ZZZ, HZ31ZZZ, HZ32ZZZ, HZ33ZZZ, HZ34ZZZ, HZ35ZZZ, HZ36ZZZ, HZ37ZZZ, HZ38ZZZ, HZ39ZZZ, HZ3BZZZ, HZ40ZZZ, HZ41ZZZ, HZ42ZZZ, HZ43ZZZ, HZ44ZZZ, HZ45ZZZ, HZ46ZZZ, HZ47ZZZ, HZ48ZZZ, HZ49ZZZ, HZ4BZZZ, HZ50ZZZ, HZ51ZZZ, HZ52ZZZ, HZ53ZZZ, HZ54ZZZ, HZ55ZZZ, HZ56ZZZ, HZ57ZZZ, HZ58ZZZ, HZ59ZZZ, HZ5BZZZ, HZ5CZZZ, HZ5DZZZ, HZ63ZZZ, HZ83ZZZ, HZ86ZZZ, HZ88ZZZ, HZ89ZZZ, HZ93ZZZ, HZ96ZZZ, HZ98ZZZ, HZ99ZZZ |
| Anemia [[1](#_ENREF_1), [2](#_ENREF_2), [4](#_ENREF_4)] | ICD-10-CM diagnosis | C94.6, D46.0, D46.1, D46.20, D46.21, D46.22, D46.4, D46.9, D46.A, D46.B, D46.C, D46.Z, D47.4, D50.0, D50.1, D50.8, D50.9, D51.0, D51.1, D51.2, D51.3, D51.8, D51.9, D52.0, D52.1, D52.8, D52.9, D53.0, D53.1, D53.2, D53.8, D53.9, D55.0, D55.1, D55.2, D55.21, D55.29, D55.3, D55.8, D55.9, D56.0, D56.1, D56.2, D56.3, D56.4, D56.5, D56.8, D56.9, D57.00, D57.01, D57.02, D57.03, D57.04, D57.09, D57.1, D57.20, D57.211, D57.212, D57.213, D57.214, D57.218, D57.219, D57.3, D57.40, D57.411, D57.412, D57.413, D57.414, D57.418, D57.419, D57.42, D57.431, D57.432, D57.433, D57.434, D57.438, D57.439, D57.44, D57.451, D57.452, D57.453, D57.454, D57.458, D57.459, D57.80, D57.811, D57.812, D57.813, D57.814, D57.818, D57.819, D58.0, D58.1, D58.2, D58.8, D58.9, D59.0, D59.1, D59.10, D59.11, D59.12, D59.13, D59.19, D59.2, D59.3, D59.30, D59.31, D59.32, D59.39, D59.4, D59.5, D59.6, D59.8, D59.9, D60.0, D60.1, D60.8, D60.9, D61.01, D61.02, D61.09, D61.1, D61.2, D61.3, D61.810, D61.811, D61.818, D61.82, D61.89, D61.9, D63.0, D63.1, D63.8, D64.0, D64.1, D64.2, D64.3, D64.4, D64.81, D64.89, D64.9, D75.81 |
| Central venous catheter[[1](#_ENREF_1), [2](#_ENREF_2)] | CPT | 36555-36569, S5520, S5522 |
|  | ICD-10-CM diagnosis | T80218A, T80219A, T80211A, T80212A |
|  | ICD-10-PCS procedure: | 02HS03Z, 02HS33Z, 02HS43Z, 02HT03Z, 02HT33Z, 02HT43Z, 02HV03Z, 02HV33Z, 02HV43Z, 05H003Z, 05H033Z, 05H043Z, 05H103Z, 05H133Z, 05H143Z, 05H303Z, 05H333Z, 05H343Z, 05H403Z, 05H433Z, 05H443Z, 05H503Z, 05H533Z, 05H543Z, 05H603Z, 05H633Z, 05H643Z, 05H703Z, 05H733Z, 05H743Z, 05H803Z, 05H833Z, 05H843Z, 05H903Z, 05H933Z, 05H943Z, 05HA03Z, 05HA33Z, 05HA43Z, 05HB03Z, 05HB33Z, 05HB43Z, 05HC03Z, 05HC33Z, 05HC43Z, 05HD03Z, 05HD33Z, 05HD43Z, 05HF03Z, 05HF33Z, 05HF43Z, 05HG03Z, 05HG33Z, 05HG43Z, 05HH03Z, 05HH33Z, 05HH43Z, 05HL03Z, 05HL33Z, 05HL43Z, 05HM03Z, 05HM33Z, 05HM43Z, 05HN03Z, 05HN33Z, 05HN43Z, 05HP03Z, 05HP33Z, 05HP43Z, 05HQ03Z, 05HQ33Z, 05HQ43Z, 05HR03Z, 05HR33Z, 05HR43Z, 05HS03Z, 05HS33Z, 05HS43Z, 05HT03Z, 05HT33Z, 05HT43Z, 05HV03Z, 05HV33Z, 05HV43Z, 05HY03Z, 05HY33Z, 05HY43Z, 06H003Z, 06H033Z, 06H043Z, 06H103Z, 06H133Z, 06H143Z, 06H203Z, 06H233Z, 06H243Z, 06H303Z, 06H333Z, 06H343Z, 06H403Z, 06H433Z, 06H443Z, 06H503Z, 06H533Z, 06H543Z, 06H603Z, 06H633Z, 06H643Z, 06H703Z, 06H733Z, 06H743Z, 06H803Z, 06H833Z, 06H843Z, 06H903Z, 06H933Z, 06H943Z, 06HB03Z, 06HB33Z, 06HB43Z, 06HC03Z, 06HC33Z, 06HC43Z, 06HD03Z, 06HD33Z, 06HD43Z, 06HF03Z, 06HF33Z, 06HF43Z, 06HG03Z, 06HG33Z, 06HG43Z, 06HH03Z, 06HH33Z, 06HH43Z, 06HJ03Z, 06HJ33Z, 06HJ43Z, 06HM03Z, 06HM33Z, 06HM43Z, 06HN03Z, 06HN33Z, 06HN43Z, 06HP03Z, 06HP33Z, 06HP43Z, 06HQ03Z, 06HQ33Z, 06HQ43Z, 06HT03Z, 06HT33Z, 06HT43Z, 06HV03Z, 06HV33Z, 06HV43Z, 06HY03Z, 06HY33Z, 06HY43Z, 0JH60XZ, 0JH63XZ, 0JH80XZ, 0JH83XZ, 0JHD0XZ, 0JHD3XZ, 0JHF0XZ, 0JHF3XZ, 0JHG0XZ, 0JHG3XZ, 0JHH0XZ, 0JHH3XZ, 0JHL0XZ, 0JHL3XZ, 0JHM0XZ, 0JHM3XZ, 0JHN0XZ, 0JHN3XZ, 0JHP0XZ, 0JHP3XZ, 4A02X4A, 4A04XB1, 4A14XB1, B5130ZA, B5131ZA, B513YZA, B513ZZA, B5140ZA, B5141ZA, B514YZA, B514ZZA, B5150ZA, B5151ZA, B515YZA, B515ZZA, B5160ZA, B5161ZA, B516YZA, B516ZZA, B5170ZA, B5171ZA, B517YZA, B517ZZA, B51B0ZA, B51B1ZA, B51BYZA, B51BZZA, B51C0ZA, B51C1ZA, B51CYZA, B51CZZA, B51D0ZA, B51D1ZA, B51DYZA, B51DZZA, B543ZZA, B544ZZA, B546ZZA, B547ZZA, B54BZZA, B54CZZA, B54DZZA |
| Cerebrovascular disease[[1](#_ENREF_1), [2](#_ENREF_2)] | CPT | 35390, 37215, 37216 |
|  | ICD-10-CM diagnosis | I602, I604, I606, I607, I608, I609, I610, I611, I612, I613, I614, I615, I616, I618, I619, I621, I629, I651, I658, I659, I663, I636, I669, I668, I638, I639, G450, G458, G451, G452, G460, G461, G462, G459, I672, G463, G464, G465, G466, G467, G468, I680, I688, I679, I6000, I6001, I6002, I6010, I6011, I6012, I6030, I6031, I6032, I6050, I6051, I6052, I6200, I6201, I6202, I6203, I6302, I6312, I6322, I6521, I6522, I6523, I6529, I6501, I6502, I6503, I6509, I6359, I6309, I6319, I6300, I6310, I6320, I6329, I6601, I6602, I6603, I6609, I6611, I6612, I6613, I6619, I6621, I6622, I6623, I6629, I6330, I6339, I6340, I6349, I6350, I6789, I6781, I6782, I6901, I6911, I6921, I6931, I6981, I6991, I6900, I6910, I6920, I6930, I6980, I6990, I63031, I63032, I63039, I63131, I63132, I63139, I63231, I63232, I63239, I63011, I63012, I63019, I63111, I63112, I63119, I63211, I63212, I63219, I63311, I63312, I63319, I63321, I63322, I63329, I63331, I63332, I63339, I63341, I63342, I63349, I63411, I63412, I63419, I63421, I63422, I63429, I63431, I63432, I63439, I63441, I63442, I63449, I63511, I63512, I63519, I63521, I63522, I63529, I63531, I63532, I63539, I63541, I63542, I63549, I67841, I67848, I69928, I69020, I69120, I69220, I69320, I69820, I69920, I69021, I69121, I69221, I69321, I69821, I69921, I69022, I69122, I69222, I69322, I69822, I69922, I69023, I69123, I69223, I69323, I69823, I69923, I69028, I69128, I69228, I69328, I69828, I69059, I69159, I69259, I69359, I69859, I69959, I69051, I69052, I69151, I69152, I69251, I69252, I69351, I69352, I69851, I69852, I69951, I69952, I69053, I69054, I69153, I69154, I69253, I69254, I69353, I69354, I69853, I69854, I69953, I69954, I69039, I69139, I69239, I69339, I69839, I69939, I69031, I69032, I69131, I69132, I69231, I69232, I69331, I69332, I69831, I69832, I69931, I69932, I69033, I69034, I69133, I69134, I69233, I69234, I69333, I69334, I69833, I69834, I69933, I69934, I69049, I69149, I69249, I69349, I69849, I69949, I69041, I69042, I69141, I69142,  I69241, I69242, I69341, I69342, I69841, I69842, I69941, I69942, I69043, I69044, I69143, I69144, I69243, I69244, I69343, I69344, I69843, I69844, I69943, I69944, I69069, I69169, I69269, I69369, I69869, I69969, I69061, I69062, I69161, I69162, I69261, I69262, I69361, I69362, I69861, I69862, I69961, I69962, I69063, I69064, I69163, I69164, I69263, I69264, I69363, I69364, I69863, I69864, I69963, I69964, I69065, I69165, I69265, I69365, I69865, I69965, I69998, I69090, I69190, I69290, I69390, I69890, I69990, I69091, I69191, I69291, I69391, I69891, I69991, I69092, I69192, I69292, I69392, I69892, I69992, I69093, I69193, I69293, I69393, I69893, I69993, I69098, I69198, I69298, I69398, I69898, G9731, G9732, I97810, I97811, I97820, I9782 |
|  | ICD-10-PCS Procedure | 03CG0ZZ, 03CG4ZZ, 03CH0ZZ, 03CH4ZZ, 03CJ0ZZ, 03CJ4ZZ, 03CK0ZZ, 03CK4ZZ, 03CL0ZZ, 03CL4ZZ, 03CM0ZZ, 03CM4ZZ, 03CN0ZZ, 03CN4ZZ, 03CP0ZZ, 03CP4ZZ, 03CQ0ZZ, 03CQ4ZZ, 03CR0ZZ, 03CR3ZZ, 03CR4ZZ, 03CS0ZZ, 03CS3ZZ, 03CS4ZZ, 03CT0ZZ, 03CT3ZZ, 03CT4ZZ, 03CU0ZZ, 03CU3ZZ, 03CU4ZZ, 03CV0ZZ, 03CV3ZZ, 03CV4ZZ, 03RH07Z, 03RH0JZ, 03RH0KZ, 03RH47Z, 03RH4JZ, 03RH4KZ, 03RJ07Z, 03RJ0JZ, 03RJ0KZ, 03RJ47Z, 03RJ4JZ, 03RJ4KZ, 03RK07Z, 03RK0JZ, 03RK0KZ, 03RK47Z, 03RK4JZ, 03RK4KZ, 03RL07Z, 03RL0JZ, 03RL0KZ, 03RL47Z, 03RL4JZ, 03RL4KZ, 03RM07Z, 03RM0JZ, 03RM0KZ, 03RM47Z, 03RM4JZ, 03RM4KZ, 03RN07Z, 03RN0JZ, 03RN0KZ, 03RN47Z, 03RN4JZ, 03RN4KZ, 03RP07Z, 03RP0JZ, 03RP0KZ, 03RP47Z, 03RP4JZ, 03RP4KZ, 03RQ07Z, 03RQ0JZ, 03RQ0KZ, 03RQ47Z, 03RQ4JZ, 03RQ4KZ, 03RR07Z, 03RR0JZ, 03RR0KZ, 03RR47Z, 03RR4JZ, 03RR4KZ, 03RS07Z, 03RS0JZ, 03RS0KZ, 03RS47Z, 03RS4JZ, 03RS4KZ, 03RT07Z, 03RT0JZ, 03RT0KZ, 03RT47Z, 03RT4JZ, 03RT4KZ, 03RU07Z, 03RU0JZ, 03RU0KZ, 03RU47Z, 03RU4JZ, 03RU4KZ, 03RV07Z, 03RV0JZ, 03RV0KZ, 03RV47Z, 03RV4JZ, 03RV4KZ, 05RM07Z, 05RM0JZ, 05RM0KZ, 05RM47Z, 05RM4JZ, 05RM4KZ, 05RN07Z, 05RN0JZ, 05RN0KZ, 05RN47Z, 05RN4JZ, 05RN4KZ, 05RP07Z, 05RP0JZ, 05RP0KZ, 05RP47Z, 05RP4JZ, 05RP4KZ, 05RQ07Z, 05RQ0JZ, 05RQ0KZ, 05RQ47Z, 05RQ4JZ, 05RQ4KZ, 05RR07Z, 05RR0JZ, 05RR0KZ, 05RR47Z, 05RR4JZ, 05RR4KZ, 05RS07Z, 05RS0JZ, 05RS0KZ, 05RS47Z, 05RS4JZ, 05RS4KZ, 05RT07Z, 05RT0JZ, 05RT0KZ, 05RT47Z, 05RT4JZ, 05RT4KZ, 05RV07Z, 05RV0JZ, 05RV0KZ, 05RV47Z, 05RV4JZ, 05RV4KZ |
| Coagulation Defects[[1](#_ENREF_1), [2](#_ENREF_2)] | ICD-10-CM diagnosis | D65, D66, D67, D68.1, D68.2, D68.0, D68.311, D68312, D68318, D69.0, D69.1, D692, D698, D699, D6832, D684, D688, D689, D693, D696, D6941, D6942, D6949, D6951, D6959, D7582, M311 |
| Ischemic Heart Disease [[4](#_ENREF_4)] [[1](#_ENREF_1), [2](#_ENREF_2)] | ICD-10-CM diagnosis | I20.0, I20.1, I20.2, I20.8, I20.81, I20.89, I24.0, I24.1, I24.8, I24.81, I24.89, I25.10, I25.110, I25.111, I25.112, I25.118, I25.119, I25.3, I25.41, I25.42, I25.5, I25.6, I25.700, I25.701, I25.702, I25.708, I25.710, I25.711, I25.712, I25.718, I25.719, I25.720, I25.721, I25.722, I25.728, I25.729, I25.730, I25.731, I25.732, I25.738, I25.739, I25.750, I25.751, I25.752, I25.758, I25.759, I25.760, I25.761, I25.762, I25.768, I25.769, I25.790, I25.791, I25.792, I25.798, I25.799, I25.810, I25.811, I25.812, I25.82, I25.83, I25.84, I25.85, I25.89, I25.9 |
| Dementia[[1](#_ENREF_1), [2](#_ENREF_2)] | ICD-10-CM diagnosis | F0390, F0150, F0151 |
| Dyspepsia [[1](#_ENREF_1), [2](#_ENREF_2)] | ICD-10-CM diagnosis | K30, R12, R100, R109, R102, R1011, R1012, R1031, R1032, R1033, R1013, R1084, R1010, R1030, R1930, R1931, R1932, R1933, R1934, R1935, R1936, R1937, R10819, R10829, R10811,  R10821, R10812, R10822, R10813, R10823, R10814, R10824, R10815, R10825, R10816, R10826, R10817, R10827 |
| Hemiplegia or Paraplegia | ICD-10-CM diagnosis | G041, G8100, G8101, G8102, G8103, G8104, G8110, G8111, G8112, G8113, G8114, G8190, G8191, G8192, G8193, G8194, G8220, G8221, G8222 |
| Hyperlipidemia[[4](#_ENREF_4)] | ICD-10-CM diagnosis | E78.0, E78.00, E78.01, E78.1, E78.2, E78.3, E78.4, E78.41, E78.49, E78.5 |
| Pneumonia [[4](#_ENREF_4)] | ICD-10-CM diagnosis | A01.03, A02.22, A06.5, A20.2, A21.2, A22.1, A31.0, A37.01, A37.11, A37.81, A37.91, A40.3, A42.0, A43.0, A48.1, A50.04, A54.84, B01.2, B05.2, B06.81, B37.1, B38.0, B38.2, B39.0, B39.2, B40.0, B40.2, B41.0, B58.3, B59, B66.4, B67.1, B77.81, B95.3, B96.0, B96.1, J09.X1, J10.00, J10.01, J10.08, J11.00, J11.08, J12.0, J12.1, J12.2, J12.3, J12.81, J12.82, J12.89, J12.9, J13, J14, J15.0, J15.1, J15.20, J15.211, J15.212, J15.29, J15.3, J15.4, J15.5, J15.6, J15.61, J15.69, J15.7, J15.8, J15.9, J16.0, J16.8, J17, J18.0, J18.1, J18.2, J18.8, J18.9, J20.0, J84.111, J84.116, J84.117, J84.178, J84.2, J85.1, J95.851, P23.0, P23.1, P23.2, P23.3, P23.4, P23.5, P23.6, P23.8, P23.9, Z87.01 |
| Rheumatologic disease/Osteoarthritis[[4](#_ENREF_4)] [[1](#_ENREF_1), [2](#_ENREF_2)] | ICD-10-CM diagnosis | L40.50, L40.51, L40.54, L40.59, M05.00, M05.011, M05.012, M05.019, M05.021, M05.022, M05.029, M05.031, M05.032, M05.039, M05.041, M05.042, M05.049, M05.051, M05.052, M05.059, M05.061, M05.062, M05.069, M05.071, M05.072, M05.079, M05.09, M05.10, M05.111, M05.112, M05.119, M05.121, M05.122, M05.129, M05.131, M05.132, M05.139, M05.141, M05.142, M05.149, M05.151, M05.152, M05.159, M05.161, M05.162, M05.169, M05.171, M05.172, M05.179, M05.19, M05.20, M05.211, M05.212, M05.219, M05.221, M05.222, M05.229, M05.231, M05.232, M05.239, M05.241, M05.242, M05.249, M05.251, M05.252, M05.259, M05.261, M05.262, M05.269, M05.271, M05.272, M05.279, M05.29, M05.30, M05.311, M05.312, M05.319, M05.321, M05.322, M05.329, M05.331, M05.332, M05.339, M05.341, M05.342, M05.349, M05.351, M05.352, M05.359, M05.361, M05.362, M05.369, M05.371, M05.372, M05.379, M05.39, M05.40, M05.411, M05.412, M05.419, M05.421, M05.422, M05.429, M05.431, M05.432, M05.439, M05.441, M05.442, M05.449, M05.451, M05.452, M05.459, M05.461, M05.462, M05.469, M05.471, M05.472, M05.479, M05.49, M05.50, M05.511, M05.512, M05.519, M05.521, M05.522, M05.529, M05.531, M05.532, M05.539, M05.541, M05.542, M05.549, M05.551, M05.552, M05.559, M05.561, M05.562, M05.569, M05.571, M05.572, M05.579, M05.59, M05.60, M05.611, M05.612, M05.619, M05.621, M05.622, M05.629, M05.631, M05.632, M05.639, M05.641, M05.642, M05.649, M05.651, M05.652, M05.659, M05.661, M05.662, M05.669, M05.671, M05.672, M05.679, M05.69, M05.70, M05.711, M05.712, M05.719, M05.721, M05.722, M05.729, M05.731, M05.732, M05.739, M05.741, M05.742, M05.749, M05.751, M05.752, M05.759, M05.761, M05.762, M05.769, M05.771, M05.772, M05.779, M05.79, M05.7A, M05.80, M05.811, M05.812, M05.819, M05.821, M05.822, M05.829, M05.831, M05.832, M05.839, M05.841, M05.842, M05.849, M05.851, M05.852, M05.859, M05.861, M05.862, M05.869, M05.871, M05.872, M05.879, M05.89, M05.8A, M05.9, M06.00, M06.011, M06.012, M06.019, M06.021, M06.022, M06.029, M06.031, M06.032, M06.039, M06.041, M06.042, M06.049, M06.051, M06.052, M06.059, M06.061, M06.062, M06.069, M06.071, M06.072, M06.079, M06.08, M06.09, M06.0A, M06.1, M06.20, M06.211, M06.212, M06.219, M06.221, M06.222, M06.229, M06.231, M06.232, M06.239, M06.241, M06.242, M06.249, M06.251, M06.252, M06.259, M06.261, M06.262, M06.269, M06.271, M06.272, M06.279, M06.28, M06.29, M06.30, M06.311, M06.312, M06.319, M06.321, M06.322, M06.329, M06.331, M06.332, M06.339, M06.341, M06.342, M06.349, M06.351, M06.352, M06.359, M06.361, M06.362, M06.369, M06.371, M06.372, M06.379, M06.38, M06.39, M06.80, M06.811, M06.812, M06.819, M06.821, M06.822, M06.829, M06.831, M06.832, M06.839, M06.841, M06.842, M06.849, M06.851, M06.852, M06.859, M06.861, M06.862, M06.869, M06.871, M06.872, M06.879, M06.88, M06.89, M06.8A, M06.9, M08.00, M08.011, M08.012, M08.019, M08.021, M08.022, M08.029, M08.031, M08.032, M08.039, M08.041, M08.042, M08.049, M08.051, M08.052, M08.059, M08.061, M08.062, M08.069, M08.071, M08.072, M08.079, M08.08, M08.09, M08.0A, M08.1, M08.20, M08.211, M08.212, M08.219, M08.221, M08.222, M08.229, M08.231, M08.232, M08.239, M08.241, M08.242, M08.249, M08.251, M08.252, M08.259, M08.261, M08.262, M08.269, M08.271, M08.272, M08.279, M08.28, M08.29, M08.2A, M08.3, M08.40, M08.411, M08.412, M08.419, M08.421, M08.422, M08.429, M08.431, M08.432, M08.439, M08.441, M08.442, M08.449, M08.451, M08.452, M08.459, M08.461, M08.462, M08.469, M08.471, M08.472, M08.479, M08.48, M08.4A, M08.80, M08.811, M08.812, M08.819, M08.821, M08.822, M08.829, M08.831, M08.832, M08.839, M08.841, M08.842, M08.849, M08.851, M08.852, M08.859, M08.861, M08.862, M08.869, M08.871, M08.872, M08.879, M08.88, M08.89, M08.90, M08.911, M08.912, M08.919, M08.921, M08.922, M08.929, M08.931, M08.932, M08.939, M08.941, M08.942, M08.949, M08.951, M08.952,M08.959, M08.961, M08.962, M08.969, M08.971, M08.972, M08.979, M08.98, M08.99, M08.9A, M15.0, M15.1, M15.2, M15.3, M15.4, M15.8, M15.9, M16.0, M16.10, M16.11, M16.12, M16.2, M16.30, M16.31, M16.32, M16.4, M16.50, M16.51, M16.52, M16.6, M16.7, M16.9, M17.0, M17.10, M17.11, M17.12, M17.2, M17.30, M17.31, M17.32, M17.4, M17.5, M17.9, M18.0, M18.10, M18.11, M18.12, M18.2, M18.30, M18.31, M18.32, M18.4, M18.50, M18.51, M18.52, M18.9, M19.011, M19.012, M19.019, M19.021, M19.022, M19.029, M19.031, M19.032, M19.039, M19.041, M19.042, M19.049, M19.071, M19.072, M19.079, M19.09, M19.111, M19.112, M19.119, M19.121, M19.122, M19.129, M19.131, M19.132, M19.139, M19.141, M19.142, M19.149, M19.171, M19.172, M19.179, M19.19, M19.211, M19.212, M19.219, M19.221, M19.222, M19.229, M19.231, M19.232, M19.239, M19.241, M19.242, M19.249, M19.271, M19.272, M19.279, M19.29, M19.90, M19.91, M19.92, M19.93, M45.0, M45.1, M45.2, M45.3, M45.4, M45.5, M45.6, M45.7, M45.8, M45.9, M45.A0, M45.A1, M45.A2, M45.A3, M45.A4, M45.A5, M45.A6, M45.A7, M45.A8, M45.AB, M46.80, M46.81, M46.82, M46.83, M46.84, M46.85, M46.86, M46.87, M46.88, M46.89, M46.90, M46.91, M46.92, M46.93, M46.94, M46.95, M46.96, M46.97, M46.98, M46.99, M47.011, M47.012, M47.013, M47.014, M47.015, M47.016, M47.019, M47.021, M47.022, M47.029, M47.10, M47.11, M47.12, M47.13, M47.14, M47.15, M47.16, M47.20, M47.21, M47.22, M47.23, M47.24, M47.25, M47.26, M47.27, M47.28, M47.811, M47.812, M47.813, M47.814, M47.815, M47.816, M47.817, M47.818, M47.819, M47.891, M47.892, M47.893, M47.894, M47.895, M47.896, M47.897, M47.898, M47.899, M47.9, M48.8X1, M48.8X2, M48.8X3, M48.8X4, M48.8X5, M48.8X6, M48.8X7, M48.8X8, M48.8X9 |
| Sleep apnea[[1](#_ENREF_1), [2](#_ENREF_2)] | ICD-10-CM diagnosis | G4733, G4730 |
| Thrombophilia[[1](#_ENREF_1), [2](#_ENREF_2)] | ICD-10-CM diagnosis | D6869, D759, D56, D45, D75.1, D6851, D6852, D6859, D6861, D6862, D68.69 |
| Varicose Veins[[1](#_ENREF_1), [2](#_ENREF_2)] | ICD-10-CM diagnosis | I8310, I8311, I8312, I8390, I8391, I8392, I8393, I83001, I83002, I83003, I83004, I83005, I83008, I83009, I83011, I83012, I83013, I83014, I83015, I83018, I83019, I83021, I83022, I83023, I83024, I83025, I83028, I83029, I83201, I83202, I83203, I83204, I83205, I83208, I83209, I83211, I83212, I83213, I83214, I83215, I83218, I83219, I83221, I83222, I83223, I83224, I83225, I83228, I83229, I83811, I83812, I83813, I83819, I83891, I83892, I83893, I83899 |
| Congestive Heart Failure[[1](#_ENREF_1), [2](#_ENREF_2)] | ICD-10-CM diagnosis | I110, I130, I132, I509, I501, I5020, I5021, I5022, I5023, I5030, I5031, I5032, I5033, I5040, I5041, I5042, I5043, I0981 |
|  | ICD-10-PCS Procedure | 02HA0QZ, 02HA0RS, 02HA0RZ, 02HA3QZ, 02HA3RS, 02HA3RZ, 02HA4QZ, 02HA4RS, 02HA4RZ, 02HN0MZ, 02HN3MZ, 02HN4MZ, 02K80ZZ, 02K83ZZ, 02K84ZZ, 02PA0QZ, 02PA0RZ, 02PA3QZ, 02PA3RZ, 02PA4QZ, 02PA4RZ, 02WA0JZ, 02WA0QZ, 02WA0RZ, 02WA3QZ, 02WA3RZ, 02WA4QZ, 02WA4RZ, 02YA0Z0, 02YA0Z1, 02YA0Z2, 0KXF0ZZ, 0KXG0ZZ, 0PT10ZZ, 0PT20ZZ, 3E053KZ, 3E063KZ, 4A023FZ, 4A02X4Z, 4A02XFZ, 5A02110, 5A02116, 5A0211D, 5A02210, 5A02216, 5A0221D |
|  | CPT | 33930, 33933, 33935, 33940, 33944, 33945, 33960, 33961, 33967, 33968, 33970, 33971, 33973, 33974, 33975, 33976, 33977, 33978, 33979, 33980, 33981, 33982, 33983, 33990, 33991, 33992, 33993 |
| Diabetes Mellitus[[4](#_ENREF_4)] [[1](#_ENREF_1), [2](#_ENREF_2)] | ICD-10-CM diagnosis | E08.00, E08.01, E08.10, E08.11, E08.21, E08.22, E08.29, E08.311, E08.319, E08.321, E08.3211, E08.3212, E08.3213, E08.3219, E08.329, E08.3291, E08.3292, E08.3293, E08.3299, E08.331, E08.3311, E08.3312, E08.3313, E08.3319, E08.339, E08.3391, E08.3392, E08.3393, E08.3399, E08.341, E08.3411, E08.3412, E08.3413, E08.3419, E08.349, E08.3491, E08.3492, E08.3493, E08.3499, E08.351, E08.3511, E08.3512, E08.3513, E08.3519, E08.3521, E08.3522, E08.3523, E08.3529, E08.3531, E08.3532, E08.3533, E08.3539, E08.3541, E08.3542, E08.3543, E08.3549, E08.3551, E08.3552, E08.3553, E08.3559, E08.359, E08.3591, E08.3592, E08.3593, E08.3599, E08.36, E08.37X1, E08.37X2, E08.37X3, E08.37X9, E08.39, E08.40, E08.41, E08.42, E08.43, E08.44, E08.49, E08.51, E08.52, E08.59, E08.610, E08.618, E08.620, E08.621, E08.622, E08.628, E08.630, E08.638, E08.641, E08.649, E08.65, E08.69, E08.8, E08.9, E09.00, E09.01, E09.10, E09.11, E09.21, E09.22, E09.29, E09.311, E09.319, E09.321, E09.3211, E09.3212, E09.3213, E09.3219, E09.329, E09.3291, E09.3292, E09.3293, E09.3299, E09.331, E09.3311, E09.3312, E09.3313, E09.3319, E09.339, E09.3391, E09.3392, E09.3393, E09.3399, E09.341, E09.3411, E09.3412, E09.3413, E09.3419, E09.349, E09.3491, E09.3492, E09.3493, E09.3499, E09.351, E09.3511, E09.3512, E09.3513, E09.3519, E09.3521, E09.3522, E09.3523, E09.3529, E09.3531, E09.3532, E09.3533, E09.3539, E09.3541, E09.3542, E09.3543, E09.3549, E09.3551, E09.3552, E09.3553, E09.3559, E09.359, E09.3591, E09.3592, E09.3593, E09.3599, E09.36, E09.37X1, E09.37X2, E09.37X3, E09.37X9, E09.39, E09.40, E09.41, E09.42, E09.43, E09.44, E09.49, E09.51, E09.52, E09.59, E09.610, E09.618, E09.620, E09.621, E09.622, E09.628, E09.630, E09.638, E09.641, E09.649, E09.65, E09.69, E09.8, E09.9, E10.10, E10.11, E10.21, E10.22, E10.29, E10.311, E10.319, E10.321, E10.3211, E10.3212, E10.3213, E10.3219, E10.329, E10.3291, E10.3292, E10.3293, E10.3299, E10.331, E10.3311, E10.3312, E10.3313, E10.3319, E10.339, E10.3391, E10.3392, E10.3393, E10.3399, E10.341, E10.3411, E10.3412, E10.3413, E10.3419, E10.349, E10.3491, E10.3492, E10.3493, E10.3499, E10.351, E10.3511, E10.3512, E10.3513, E10.3519, E10.3521, E10.3522, E10.3523, E10.3529, E10.3531, E10.3532, E10.3533, E10.3539, E10.3541, E10.3542, E10.3543, E10.3549, E10.3551, E10.3552, E10.3553, E10.3559, E10.359, E10.3591, E10.3592, E10.3593, E10.3599, E10.36, E10.37X1, E10.37X2, E10.37X3, E10.37X9, E10.39, E10.40, E10.41, E10.42, E10.43, E10.44, E10.49, E10.51, E10.52, E10.59, E10.610, E10.618, E10.620, E10.621, E10.622, E10.628, E10.630, E10.638, E10.641, E10.649, E10.65, E10.69, E10.8, E10.9, E11.00, E11.01, E11.10, E11.11, E11.21, E11.22, E11.29, E11.311, E11.319, E11.321, E11.3211, E11.3212, E11.3213, E11.3219, E11.329, E11.3291, E11.3292, E11.3293, E11.3299, E11.331, E11.3311, E11.3312, E11.3313, E11.3319, E11.339, E11.3391, E11.3392, E11.3393, E11.3399, E11.341, E11.3411, E11.3412, E11.3413, E11.3419, E11.349, E11.3491, E11.3492, E11.3493, E11.3499, E11.351, E11.3511, E11.3512, E11.3513, E11.3519, E11.3521, E11.3522, E11.3523, E11.3529, E11.3531, E11.3532, E11.3533, E11.3539, E11.3541, E11.3542, E11.3543, E11.3549, E11.3551, E11.3552, E11.3553, E11.3559, E11.359, E11.3591, E11.3592, E11.3593, E11.3599, E11.36, E11.37X1, E11.37X2, E11.37X3, E11.37X9, E11.39, E11.40, E11.41, E11.42, E11.43, E11.44, E11.49, E11.51, E11.52, E11.59, E11.610, E11.618, E11.620, E11.621, E11.622, E11.628, E11.630, E11.638, E11.641, E11.649, E11.65, E11.69, E11.8, E11.9, E13.00, E13.01, E13.10, E13.11, E13.21, E13.22, E13.29, E13.311, E13.319, E13.321, E13.3211, E13.3212, E13.3213, E13.3219, E13.329, E13.3291, E13.3292, E13.3293, E13.3299, E13.331, E13.3311, E13.3312, E13.3313, E13.3319, E13.339, E13.3391, E13.3392, E13.3393, E13.3399, E13.341, E13.3411, E13.3412, E13.3413, E13.3419, E13.349, E13.3491, E13.3492, E13.3493, E13.3499, E13.351, E13.3511, E13.3512, E13.3513, E13.3519, E13.3521, E13.3522, E13.3523, E13.3529, E13.3531, E13.3532, E13.3533, E13.3539, E13.3541, E13.3542, E13.3543, E13.3549, E13.3551, E13.3552, E13.3553, E13.3559, E13.359, E13.3591, E13.3592, E13.3593, E13.3599, E13.36, E13.39, E13.40, E13.41, E13.42, E13.43, E13.44, E13.49, E13.51, E13.52, E13.59, E13.610, E13.618, E13.620, E13.621, E13.622, E13.628, E13.630, E13.638, E13.641, E13.649, E13.65, E13.69, E13.8, E13.9 |
| Hypertension [[1](#_ENREF_1), [2](#_ENREF_2), [4](#_ENREF_4)] | ICD-10-CM diagnosis | H35.031, H35.032, H35.033, H35.039, I10, I11.0, I11.9, I12.0, I12.9, I13.0, I13.10, I13.11, I13.2, I15.0, I15.1, I15.2, I15.8, I15.9, I1A.0, I67.4, N26.2 |
| Renal Disease [[1](#_ENREF_1), [2](#_ENREF_2)] | ICD-10-CM diagnosis | ICD-10 diagnosis: N08, N16, N19, B520, I129, K767, N044, N021, N022, N023, N041, N042, N024, N025, N026, N027, N043, N045,  N046, N020, N040, N028, N047, N048, N029, N049, N032, N031, N033, N034, N035, N036, N037, N038, N030, N039, N059, N052, N062, N072, N053, N054, N055, N063, N064, N065, N073, N074, N075, N171, N172, N050, N051, N056, N057, N058, N060, N061, N066, N067, N068, N070, N071, N076, N077, N078, N140, N141, N142, N143, N144, N150, N158, N069, N079, N159, N181, N182, N183, N184, N185, N186, N189, N250, N251, N259, R800, R801, R803, R808, R809, M3214, M3215, M3504, Z4822, E1121, E1122, E1129, E1321, E1322, E1329, E1021, E1022, E1029, I1310, N2581, N2589, Z940, Z992, Z9115, Z4931, Z4901, Z4902, Z4932 |
|  | ICD-10-PCS Procedure | 031209D, 031209F, 03120AD, 03120AF, 03120JD, 03120JF, 03120KD, 03120KF, 03120ZD, 03120ZF, 031309D, 031309F, 03130AD, 03130AF, 03130JD, 03130JF, 03130KD, 03130KF, 03130ZD, 03130ZF, 031409D, 031409F, 03140AD, 03140AF, 03140JD, 03140JF, 03140KD, 03140KF, 03140ZD, 03140ZF, 031509D, 031509F, 03150AD, 03150AF, 03150JD, 03150JF, 03150KD, 03150KF, 03150ZD, 03150ZF, 031609D, 031609F, 03160AD, 03160AF, 03160JD, 03160JF, 03160KD, 03160KF, 03160ZD, 03160ZF, 031709D, 031709F, 03170AD, 03170AF, 03170JD, 03170JF, 03170KD, 03170KF, 03170ZD, 03170ZF, 031809D, 031809F, 03180AD, 03180AF, 03180JD, 03180JF, 03180KD, 03180KF, 03180ZD, 03180ZF, 031909F, 03190AF, 03190JF, 03190KF, 03190ZF, 031A09F, 031A0AF, 031A0JF, 031A0KF, 031A0ZF, 031B09F, 031B0AF, 031B0JF, 031B0KF, 031B0ZF, 031C09F, 031C0AF, 031C0JF, 031C0KF, 031C0ZF, 03PY07Z, 03PY0JZ, 03PY0KZ, 03PY37Z, 03PY3JZ, 03PY3KZ, 03PY47Z, 03PY4JZ, 03PY4KZ, 05HY33Z, 06HY33Z, 0TY00Z0, 0TY00Z1, 0TY00Z2, 0TY10Z0, 0TY10Z1, 0TY10Z2, 3E1M39Z, 5A1D70Z, 5A1D80Z, 5A1D90Z |
|  | CPT | 36147, 36148, 36800-36821, 36825, 36830-36833, 36838, 36870, 90918-90925, 90935-90999, 50340, 50360, 50365, 50370 |
| Liver Disease [[1](#_ENREF_1), [2](#_ENREF_2)] | ICD-10-CM diagnosis | ICD-10-CM Codes: K77, B251, B181, B180, B170, B172, B182, B178, B188, B189, B190, B179, B199, E804, E805, E806, E807, K700, K702, K709, K739, K730, K754, K731, K732, K738, K740, K743, K744, K745, K760, K741, K742, K769, K750, K751, K766, K767, K761, K710, K712, K713, K714, K716, K717, K718, K719, K752, K753, K759, K764, K763, K765, K7291, K7041, K7111, K7201, K7211, Z4823, B1920, B1921, I8501, I8500, I8511, I8510, K7010, K7011, K7030, K7031, K7040, K7460, K7469, K7581, K7689, K7290, K7210, K7110, K7150, K7151, K7589, K7681, T8640, T8641, T8642, T8643, T8649, Z944 |
|  | ICD-10-PCS Procedure | ICD-10-PCS Codes**:** 0610075, 0610075, 0610076, 0610076, 061007Y, 0610095, 0610095, 0610096, 0610096, 061009Y, 06100A5, 06100A6, 06100AY, 06100J5, 06100J6, 06100JY, 06100K5, 06100K6, 06100KY, 06100Z5, 06100Z6, 06100ZY, 0610475, 0610475, 0610476, 0610476, 061047Y, 0610495, 0610495, 0610496, 0610496, 061049Y, 06104A5, 06104A6, 06104AY, 06104J5, 06104J6, 06104JY, 06104K5, 06104K6, 06104KY, 06104Z5, 06104Z6, 06104ZY, 0611079, 0611079, 061107B, 061107Y, 0611099, 0611099, 061109B, 061109Y, 06110A9, 06110AB, 06110AY, 06110J9, 06110JB, 06110JY, 06110K9, 06110KB, 06110KY, 06110Z9, 06110ZB, 06110ZY,  0611479, 0611479, 061147B, 061147Y, 0611499, 0611499, 061149B, 061149Y, 06114A9, 06114AB, 06114AY, 06114J9, 06114JB, 06114JY, 06114K9, 06114KB, 06114KY, 06114Z9, 06114ZB, 06114ZY, 061207Y, 061209Y, 06120AY, 06120JY, 06120KY, 06120ZY, 061247Y, 061249Y, 06124AY, 06124JY, 06124KY, 06124ZY, 061407Y, 061409Y, 06140AY, 06140JY, 06140KY, 06140ZY, 061447Y, 061449Y, 06144AY, 06144JY, 06144KY, 06144ZY, 061507Y, 061509Y, 06150AY, 06150JY, 06150KY, 06150ZY, 061547Y, 061549Y, 06154AY, 06154JY, 06154KY, 06154ZY, 061607Y, 061609Y, 06160AY, 06160JY, 06160KY, 06160ZY, 061647Y, 061649Y, 06164AY, 06164JY, 06164KY, 06164ZY, 061707Y, 061709Y, 06170AY, 06170JY, 06170KY, 06170ZY, 061747Y, 061749Y, 06174AY, 06174JY, 06174KY, 06174ZY, 0618079, 0618079, 061807B, 061807Y, 0618099, 0618099, 061809B, 061809Y, 06180A9, 06180AB, 06180AY, 06180J9, 06180JB, 06180JY, 06180K9, 06180KB, 06180KY, 06180Z9, 06180ZB, 06180ZY, 0618479, 0618479, 061847B, 061847Y, 0618499, 0618499, 061849B, 061849Y, 06184A9, 06184AB, 06184AY, 06184J9, 06184JB, 06184JY, 06184K9, 06184KB, 06184KY, 06184Z9, 06184ZB, 06184ZY, 061907Y, 061909Y, 06190AY, 06190JY, 06190KY, 06190ZY, 061947Y, 061949Y, 06194AY, 06194JY, 06194KY, 06194ZY, 061B07Y, 061B09Y, 061B0AY, 061B0JY, 061B0KY, 061B0ZY, 061B47Y, 061B49Y, 061B4AY, 061B4JY, 061B4KY, 061B4ZY, 061J07Y, 061J09Y, 061J0AY, 061J0JY, 061J0KY, 061J0ZY, 061J47Y, 061J49Y, 061J4AY, 061J4JY, 061J4KY, 061J4ZY, 06L30ZZ, 06L33ZZ, 06L34ZZ, 0FY00Z0, 0FY00Z1, 0FY00Z2 |
|  | CPT | 47135, 47136 |
| Chronic Obstructive Pulmonary Disease (COPD)[[1](#_ENREF_1), [2](#_ENREF_2), [4](#_ENREF_4)] | ICD-10-CM diagnosis | J40, J41.0, J41.1, J41.8, J42, J43.0, J43.1, J43.2, J43.9, J44.0, J44.1, J44.81, J44.89, J44.9, J47.0, J47.1, J47.9, J98.2, J98.3 |
| Peptic Ulcer Disease[[1](#_ENREF_1), [2](#_ENREF_2)] | ICD-10-CM diagnosis | K250, K251, K252, K253, K254, K255, K256, K257, K259, K260, K261, K262, K263, K264, K265, K266, K267, K269, K270, K271, K272, K273, K274, K275, K276, K277, K279, K280, K281, K282, K283, K284, K285, K286, K287, K289 |
| Inflammatory Bowel Disease [[1](#_ENREF_1), [2](#_ENREF_2)] | ICD-10-CM diagnosis | K5000, K5010, K5080, K5090, K5180, K5120, K5130, K5140, K5150, K5100, K5190, K50012, K50013, K50014, K50112, K50113, K50114, K50812, K50813, K50814, K50912, K50913, K50914, K51012, K51013, K51014, K51212, K51213, K51214, K51312, K51313, K51314, K51412, K51413, K51414, K51512, K51513, K51514, K51812, K51813, K51814, K51912, K51913, K51914, K50011, K50018, K50019, K50111, K50118, K50119, K50811, K50818, K50819, K50911, K50918, K50919, K51211, K51218, K51219, K51311, K51318, K51319, K51411, K51418, K51419, K51511, K51518, K51519, K51011, K51018, K51019, K51811, K51818, K51819, K51911, K51918, K51919 |
| Peripheral Vascular Disease [[1](#_ENREF_1), [2](#_ENREF_2)] | ICD-10-CM diagnosis | I700, I701, I708, I711, I712, I713, I714, I718, I715, I716, I719, I790, I721, I722, I723, I724, I720, I728, I729, I731, I670, I791, I798, I739, I742, I743, I744, I745, I748, I749, I7025, I7035, I7045, I7055, I7065, I7075, I7301, I7092, I7090, I7091, I7100, I7101, I7102, I7103, I7300, I7771, I7772, I7773, I7774, I7779, I7381, I7389, I7401, I7409, I7410, I7419, I7411, I7581, I7589, I70231, I70232, I70233, I70234, I70235, I70238, I70239, I70241, I70242, I70243, I70244, I70245, I70248, I70249, I70331, I70332, I70333, I70334, I70335, I70338, I70339, I70341, I70342, I70343, I70344, I70345, I70348, I70349, I70361, I70362, I70363, I70368, I70369, I70431, I70432, I70433, I70434, I70435, I70438, I70439, I70441, I70442, I70443, I70444, I70445, I70448, I70449, I70461, I70462, I70463, I70468, I70469, I70531, I70532, I70533, I70534, I70535, I70538, I70539, I70541, I70542, I70543, I70544, I70545, I70548, I70549, I70561, I70562, I70563, I70568, I70569, I70631, I70632, I70633, I70634, I70635, I70638, I70639, I70641, I70642, I70643, I70644, I70645, I70648, I70649, I70661, I70662, I70663, I70668, I70669, I70731, I70732, I70733, I70734, I70735, I70738, I70739, I70741, I70742, I70743, I70744, I70745, I70748, I70749, I70761, I70762, I70763, I70768, I70769, I70201, I70202, I70203, I70208, I70209, I70211, I70212, I70213, I70218, I70219, I70221, I70222, I70223, I70228, I70229, I70261, I70262, I70263, I70268, I70269, I70291, I70292, I70293, I70298, I70299, I70301, I70302, I70303, I70308, I70309, I70311, I70312, I70313, I70318, I70319, I70321, I70322, I70323, I70328, I70329, I70391, I70392, I70393, I70398, I70399, I70601, I70602, I70603, I70608, I70609, I70611, I70612, I70613, I70618, I70619, I70621, I70622, I70623, I70628, I70629, I70691, I70692, I70693, I70698, I70699, I70701, I70702, I70703, I70708, I70709, I70711, I70712, I70713, I70718, I70719, I70721, I70722, I70723, I70728, I70729, I70791, I70792, I70793, I70798, I70799, I70401, I70402, I70403, I70408, I70409, I70411, I70412, I70413, I70418, I70419, I70421, I70422, I70423, I70428, I70429, I70491, I70492, I70493, I70498, I70499, I70501, I70502, I70503, I70508, I70509, I70511, I70512, I70513, I70518, I70519, I70521, I70522, I70523, I70528, I70529, I70591, I70592, I70593, I70598, I70599, I75011, I75012, I75013, I75019, I75021, I75022, I75023, I75029 |
| Hip/Pelvic fractures[[4](#_ENREF_4)] [[1](#_ENREF_1), [2](#_ENREF_2)] | ICD-10-CM diagnosis | M80.051A, M80.052A, M80.059A, M80.0B1A, M80.0B2A, M80.0B9A, M80.851A, M80.852A, M80.859A, M80.8B1A, M80.8B2A, M80.8B9A, M84.350A, M84.351A, M84.352A, M84.353A, M84.359A, M84.451A, M84.452A, M84.453A, M84.459A, M84.550A, M84.551A, M84.552A, M84.553A, M84.559A, M84.650A, M84.651A, M84.652A, M84.653A, M84.659A, M97.01XA, M97.02XA, S32.301A, S32.301B, S32.302A, S32.302B, S32.309A, S32.309B, S32.311A, S32.311B, S32.312A, S32.312B, S32.313A, S32.313B, S32.314A, S32.314B, S32.315A, S32.315B, S32.316A, S32.316B, S32.391A, S32.391B, S32.392A, S32.392B, S32.399A, S32.399B, S32.401A, S32.401B, S32.402A, S32.402B, S32.409A, S32.409B, S32.411A, S32.411B, S32.412A, S32.412B, S32.413A, S32.413B, S32.414A, S32.414B, S32.415A, S32.415B, S32.416A, S32.416B, S32.421A, S32.421B, S32.422A, S32.422B, S32.423A, S32.423B, S32.424A, S32.424B, S32.425A, S32.425B, S32.426A, S32.426B, S32.431A, S32.431B, S32.432A, S32.432B, S32.433A, S32.433B, S32.434A, S32.434B, S32.435A, S32.435B, S32.436A, S32.436B, S32.441A, S32.441B, S32.442A, S32.442B, S32.443A, S32.443B, S32.444A, S32.444B, S32.445A, S32.445B, S32.446A, S32.446B, S32.451A, S32.451B, S32.452A, S32.452B, S32.453A, S32.453B, S32.454A, S32.454B, S32.455A, S32.455B, S32.456A, S32.456B, S32.461A, S32.461B, S32.462A, S32.462B, S32.463A, S32.463B, S32.464A, S32.464B, S32.465A, S32.465B, S32.466A, S32.466B, S32.471A, S32.471B, S32.472A, S32.472B, S32.473A, S32.473B, S32.474A, S32.474B, S32.475A, S32.475B, S32.476A, S32.476B, S32.481A, S32.481B, S32.482A, S32.482B, S32.483A, S32.483B, S32.484A, S32.484B, S32.485A, S32.485B, S32.486A, S32.486B, S32.491A, S32.491B, S32.492A, S32.492B, S32.499A, S32.499B, S32.501A, S32.501B, S32.502A, S32.502B, S32.509A, S32.509B, S32.511A, S32.511B, S32.512A, S32.512B, S32.519A, S32.519B, S32.591A, S32.591B, S32.592A, S32.592B, S32.599A, S32.599B, S32.601A, S32.601B, S32.602A, S32.602B, S32.609A, S32.609B, S32.611A, S32.611B, S32.612A, S32.612B, S32.613A, S32.613B, S32.614A, S32.614B, S32.615A, S32.615B, S32.616A, S32.616B, S32.691A, S32.691B, S32.692A, S32.692B, S32.699A, S32.699B, S32.810A, S32.810B, S32.811A, S32.811B, S32.82XA, S32.82XB, S32.89XA, S32.89XB, S32.9XXA, S32.9XXB, S72.001A, S72.001B, S72.001C, S72.002A, S72.002B, S72.002C, S72.009A, S72.009B, S72.009C, S72.011A, S72.011B, S72.011C, S72.012A, S72.012B, S72.012C, S72.019A, S72.019B, S72.019C, S72.021A, S72.021B, S72.021C, S72.022A, S72.022B, S72.022C, S72.023A, S72.023B, S72.023C, S72.024A, S72.024B, S72.024C, S72.025A, S72.025B, S72.025C, S72.026A, S72.026B, S72.026C, S72.031A, S72.031B, S72.031C, S72.032A, S72.032B, S72.032C, S72.033A, S72.033B, S72.033C, S72.034A, S72.034B, S72.034C, S72.035A, S72.035B, S72.035C, S72.036A, S72.036B, S72.036C, S72.041A, S72.041B, S72.041C, S72.042A, S72.042B, S72.042C, S72.043A, S72.043B, S72.043C, S72.044A, S72.044B, S72.044C, S72.045A, S72.045B, S72.045C, S72.046A, S72.046B, S72.046C, S72.051A, S72.051B, S72.051C, S72.052A, S72.052B, S72.052C, S72.059A, S72.059B, S72.059C, S72.061A, S72.061B, S72.061C, S72.062A, S72.062B, S72.062C, S72.063A, S72.063B, S72.063C, S72.064A, S72.064B, S72.064C, S72.065A, S72.065B, S72.065C, S72.066A, S72.066B, S72.066C, S72.091A, S72.091B, S72.091C, S72.092A, S72.092B, S72.092C, S72.099A, S72.099B, S72.099C, S72.101A, S72.101B, S72.101C, S72.102A, S72.102B, S72.102C, S72.109A, S72.109B, S72.109C, S72.111A, S72.111B, S72.111C, S72.112A, S72.112B, S72.112C, S72.113A, S72.113B, S72.113C, S72.114A, S72.114B, S72.114C, S72.115A, S72.115B, S72.115C, S72.116A, S72.116B, S72.116C, S72.121A, S72.121B, S72.121C, S72.122A, S72.122B, S72.122C, S72.123A, S72.123B, S72.123C, S72.124A, S72.124B, S72.124C, S72.125A, S72.125B, S72.125C, S72.126A, S72.126B, S72.126C, S72.131A, S72.131B, S72.131C, S72.132A, S72.132B, S72.132C, S72.133A, S72.133B, S72.133C, S72.134A, S72.134B, S72.134C, S72.135A, S72.135B, S72.135C, S72.136A, S72.136B, S72.136C, S72.141A, S72.141B, S72.141C, S72.142A, S72.142B, S72.142C, S72.143A, S72.143B, S72.143C, S72.144A, S72.144B, S72.144C, S72.145A, S72.145B, S72.145C, S72.146A, S72.146B, S72.146C, S72.21XA, S72.21XB, S72.21XC, S72.22XA, S72.22XB, S72.22XC, S72.23XA, S72.23XB, S72.23XC, S72.24XA, S72.24XB, S72.24XC, S72.25XA, S72.25XB, S72.25XC, S72.26XA, S72.26XB, S72.26XC, S79.001A, S79.002A, S79.009A, S79.011A, S79.012A, S79.019A, S79.091A, S79.092A, S79.099A |
| Recent history of falls[[1](#_ENREF_1), [2](#_ENREF_2)] | ICD-10-CM diagnosis | W100XXA, W100XXD, W101XXA, W101XXD, W102XXA, W102XXD, W108XXA, W108XXD, W109XXA, W109XXD, W07XXXA, W07XXXD, W050XXA, W050XXD, W051XXA, W051XXD, W052XXA, W052XXD, W06XXXA, W06XXXD, W08XXXA, W08XXXD, W1811XA, W1811XD, W1812XA, W1812XD, W001XXA, W001XXD, W002XXA, W002XXD, W1789XA, W1789XD, W000XXA, W000XXD, W009XXA, W009XXD, W010XXA, W010XXD, W182XXA, W182XXD, W03XXXA, W03XXXD, W19XXXA, W0110XA, W0110XD, W01110A, W01110D, W01111A, W01111D, W01118A, W01118D, W01119A, W01119D, W1802XA, W1802XD, W01190A, W01190D, W01198A, W01198D, W1800XA, W1800XD, W1809XD, W04XXXA, W04XXXD, W1830XA, W1830XD, W1831XA, W1831XD, W1839XA, W1839XD, W19XXXD, W11XXXA, W11XXXD |
| **List of Baseline Bleeding Codes[**[**1**](#_ENREF_1)**,** [**2**](#_ENREF_2)**]** | | |
| Baseline gastrointestinal bleed[[1](#_ENREF_1), [2](#_ENREF_2)] | ICD-10-CM diagnosis | I8501 , I8511 , K2211 , K226 , K250 , K252 , K254 , K256 , K260 , K262 , K264 , K266 , K270 , K272 , K274 , K276 , K280 , K282 , K284 , K286 , K2901 , K2921 , K2931 , K2941 , K2951 , K2961 , K2971 , K2981 , K2991 , K31811 , K3182 , K5521 , K5701 , K5711 , K5713 , K5721 , K5731 , K5733 , K5741 , K5751 , K5753 , K5781 , K5791 , K5793 , K625 , K6381 , K661 , K920 , K921 , K922 , R041 , R58, |
|  | CPT | 43227, 43255, 43501, 44366, 44378, 44391, 45317, 45334, 45382, 46614 |
|  | ICD-10-PCS Procedure | 04L23DZ, 06L13DZ, 06L43DZ, 06L53DZ, 0DQ60ZZ, 0DQ63ZZ, 0DQ64ZZ, 0DQ67ZZ, 0DQ68ZZ, 0DQ90ZZ, 0DQ93ZZ, 0DQ94ZZ, 0DQ97ZZ, 0DQ98ZZ, 0W3G0ZZ, 0W3G3ZZ, 0W3G4ZZ, 0W3P8ZZ |
| Baseline intracranial bleed[[1](#_ENREF_1), [2](#_ENREF_2)] | ICD-10-CM diagnosis | I6000, I6001, I6002, I6010, I6011, I6012, I602, I6030, I6031, I6032, I604, I6050, I6051, I6052, I606, I607, I608, I609, I610, I611, I612, I613, I614, I615, I616, I618, I619, I6200, I6201, I6202, I6203, I621, I629, S0190XA, S062X0A, S062X1A, S062X2A, S062X3A, S062X4A, S062X5A, S062X6A, S062X7A, S062X8A, S062X9A, S06300A, S06301A, S06302A, S06303A, S06304A, S06305A, S06306A, S06307A, S06308A, S06309A, S06340A, S06341A, S06342A, S06343A, S06344A, S06345A, S06346A, S06347A, S06348A, S06349A, S06350A, S06351A, S06352A, S06353A, S06354A, S06355A, S06356A, S06357A, S06358A, S06359A, S06360A, S06361A, S06362A, S06363A, S06364A, S06365A, S06366A, S06367A, S06368A, S06369A, S064X0A, S064X1A, S064X2A, S064X3A, S064X4A, S064X5A, S064X6A, S064X7A, S064X8A, S064X9A, S065X0A, S065X1A, S065X2A, S065X3A, S065X4A, S065X5A, S065X6A, S065X7A, S065X8A, , S066X0A, S066X1A, S066X2A, S066X3A, S065X9A S066X4A, S066X5A, S066X6A, S066X7A, S066X8A, S066X9A, S06890A, S06891A, S06892A, S06893A, S06894A, S06895A, S06896A, S06897A,  S06898A, S06899A, S069X0A, S069X1A, S069X2A, S069X3A, S069X4A, S069X5A, S069X6A, S069X7A, S069X8A, S069X9A |
| Baseline other bleed[[1](#_ENREF_1), [2](#_ENREF_2)] | ICD-10-CM diagnosis | A985, D62, D7801, D7802, D7821, D7822, E3601, E3602, E89810, E89811, G9731, G9732, G9751, G9752, H05231, H05232, H05233, H05239, H1130, H1131, H1132, H1133, H2100, H2101, H2102, H2103, H31301, H31302, H31303, H31309, H31311, H31312, H31313, H31319, H31411, H31412, H31413, H31419, H3560, H3561, H3562, H3563, H35731, H35732, H35733, H35739, H4310, H4311, H4312, H4313, H44811, H44812, H44813, H44819, H47021, H47022, H47023, H47029, H59111, H59112, H59113, H59119, H59121, H59122, H59123, H59129, H59311, H59312, H59313, H59319, H59321, H59322, H59323, H59329, H61121, H61122, H61123, H61129, H9521, H9522, H9541, H9542, I312, I97410, I97411, I97418, I9742, I97610, I97611, I97618, I97620, J9561, J9562, J95830, J95831, K661, K9161, K9162, K91840, K91841, L7601, L7602, L7621, L7622, M2500, M25011, M25012, M25019, M25021, M25022, M25029, M25031, M25032, M25039, M25041, M25042, M25049, M25051, M25052, M25059, M25061, M25062, M25069, M25071, M25072, M25073, M25074, M25075, M25076, M2508, M96810, M96811, M96830, M96831, N421, N837, N857, N920, N9961, N9962, N99820, N99821, R040, R041, R042, R0489, R049, R310, R319, R58, R710, S36112A, S37021A, S37022A, S37029A, T792XXA |
|  | CPT | CPT codes: 30901, 30903, 30905, 31238, 42970, 32658, 33020, 47350-47362, 65815, 65930 |
|  | ICD-10-PCS Procedure | 2Y41X5Z, 30230H1, 30230N1, 30230P1, 30233H1, 30233N1, 30233P1, 30240H1, 30240N1, 30240P1, 30243H1, 30243N1, 30243P1, 30250H1, 30250N1, 30250P1, 30253H1, 30253N1,  30253P1, 30260H1, 30260N1, 30260P1, 30263H1, 30263N1, 30263P1 |
| **Cancer type and sites of occurrences[**[**1**](#_ENREF_1)**,** [**2**](#_ENREF_2)**]** | | |
| Leukemia[[1](#_ENREF_1), [2](#_ENREF_2)] | ICD-10-CM diagnosis | C91, C92, C93, C94, C95 |
| Bladder[[1](#_ENREF_1), [2](#_ENREF_2)] | ICD-10-CM diagnosis | C67 |
| Brain Tumor[[1](#_ENREF_1), [2](#_ENREF_2)] | ICD-10-CM diagnosis | C69-C71, D43.2, D43.4, D42.0, D42.1, D42.9, D49.6 |
| Gynecologic (uterus, cervix, placenta, ovary, other female genital organs) [[1](#_ENREF_1), [2](#_ENREF_2)] | ICD-10-CM diagnosis | C51-C58 |
| Lung[[1](#_ENREF_1), [2](#_ENREF_2)] | ICD-10-CM diagnosis | C34.00, C34.01, C34.02, C34.10, C34.11, C34.12, C34.2, C34.30, C34.31, C34.32, C34.80, C34.81, C34.82, C34.90, C34.91, C34.92, D02.20, D02.21, D02.22 |
| Lymphoma[[1](#_ENREF_1), [2](#_ENREF_2)] | ICD-10-CM diagnosis | C81-C88 |
| Pancreas[[1](#_ENREF_1), [2](#_ENREF_2)] | ICD-10-CM diagnosis | C25 |
| Renal Cell Carcinoma[[1](#_ENREF_1), [2](#_ENREF_2)] | ICD-10-CM diagnosis | C64, C65, C66, C68 |
| Stomach[[1](#_ENREF_1), [2](#_ENREF_2)] | ICD-10-CM diagnosis | C16 |
| Testicular[[1](#_ENREF_1), [2](#_ENREF_2)] | ICD-10-CM diagnosis | C62 |
| Gastrointestinal Cancer (GI; esophagus, stomach, biliary system, pancreas, small intestine, large intestine, rectum and anus) [[1](#_ENREF_1), [2](#_ENREF_2)] | ICD-10-CM diagnosis | C15-C25 |
| Upper GI cancer (esophagus, stomach) [[1](#_ENREF_1), [2](#_ENREF_2)] | ICD-10-CM diagnosis | C15, C16 |
| Lower GI cancer (small intestine, large intestine, rectum and anus) [[1](#_ENREF_1), [2](#_ENREF_2)] | ICD-10-CM diagnosis | C17, C18, C19. C20, C21 |
| Female Breast Cancer[[1](#_ENREF_1), [2](#_ENREF_2)] | ICD-10-CM diagnosis | C50, D05.00, D05.01, D05.02, D05.10, D05.11, D05.12, D05.80, D05.81, D05.82, D05.90, D05.91, D05.92 |
| Prostate Cancer[[1](#_ENREF_1), [2](#_ENREF_2)] | ICD-10-CM diagnosis | C61, D07.5 |
| Multiple Myeloma[[1](#_ENREF_1), [2](#_ENREF_2)] | ICD-10-CM diagnosis | C90.0 |
| Metastatic Cancer Diagnosis[[1](#_ENREF_1), [2](#_ENREF_2)] | ICD-10-CM diagnosis | C770, C771, C772, C773, C774, C775, C778, C779, C7800, C7801, C7802, C781, C782, C7830, C7839, C784, C785, C786, C787, C7880, C7889, C7900, C7901, C7902, C7910, C7911, C7919, C792, C7931, C7932, C7940, C7949, C7951, C7952, C7960, C7961, C7962, C7963, C7970, C7971, C7972, C7981, C7982, C7989, C799 |

AIDS: acquired immunodeficiency syndrome; COPD: chronic obstructive pulmonary disease; CPT: Current Procedural Terminology; GI: gastrointestinal; HCPCS: Healthcare Common Procedure Coding System; ICD-10-CM: International Classification of Diseases, 10th Revision, Clinical Modification; ICD-10-PCS: International Classification of Diseases, 10th Revision, Procedure Coding System.

Table H. List of baseline medication codes

| **Drug** | **NDCs** | **HCPCS Codes*** |
| --- | --- | --- |
| ACE (angiotensin converting enzyme) inhibitors and ARBs (angiotensin receptor blockers)[[1](#_ENREF_1), [2](#_ENREF_2)] | Benazepril, Captopril, Enalapril, Fosinopril, Lisinopril , Moexipril, Perindopril, Quinapril, Ramipril, Trandolapril, azilsartan, candesartan, eprosartan, irbesartan, losartan, olmesartan, telmisartan, valsartan, | N/A |
| Antiarrhythmic[[1](#_ENREF_1), [2](#_ENREF_2)] | Alinidine, Bretylium, Digitalis, Diltiazem, Diphenylhydantoin, Disopyramide, Dofetilide, Encainide, Flecainide, Mexiletine, Moricizine, Nadolol, Propafenone, Quinidine, Tocainide, Verapamil | N/A |
|  | Adenosine | J0150, J0151, J0152, J0153 |
|  | Amiodarone | J0282 |
|  | Atropine | J0461, J7635, J7636 |
|  | Digoxin | J1160, J1162 |
|  | Ibutilide | J1742 |
|  | Lidocaine | C9285, J2001 |
|  | Procainamide | J2690 |
|  | Sotalol | C9482 |
| Antiplatelet[[1](#_ENREF_1), [2](#_ENREF_2)] | Abciximab | J0130 |
|  | Anagrelide Hydrochloride | N/A |
|  | Aspirin/Dipyridamole | G8598, G8599, G8895, G8896, G8897, G9277, G9278, G9435, G9436, G9437, G9793, G9794, G9795 |
|  | Cilostazol | G9531 |
|  | Clopidogrel Hydrogen Sulfate | G9531 |
|  | Dipyridamole | G9531, J1245 |
|  | Eptifibatide | J1327 |
|  | Prasugrel Hydrochloride | G9531 |
|  | Ticagrelor | G9531 |
|  | Ticlopidine Hydrochloride | G9531 |
|  | Tirofiban Hydrochloride | J3246 |
| Aromatase inhibitors[[1](#_ENREF_1), [2](#_ENREF_2)] | Arimidex, aromasin, femara | N/A |
| Beta blockers[[1](#_ENREF_1), [2](#_ENREF_2)] | Acebutolol , Atenolol, Betaxolol, Bisoprolol, Esmolol, Carteolol, Carvedilol, Labetalol, Levobunolol, Metipranolol, Metoprolol, Nebivolol, Nadolol, Penbutolol, Pindolol, Propranolol, Sotalol, Timolol, | N/A |
| Gastroprotective agents[[1](#_ENREF_1), [2](#_ENREF_2)] | Celecoxib, ketoprofen, meloxicam, etoricoxib, misoprostol, dexlansoprazole, esomeprazole, esomeprazole, lansoprazole, omeprazole, pantoprazole, rabeprazole | N/A |
| Nonsteroidal anti-inflammatory drug (NSAID)[[1](#_ENREF_1), [2](#_ENREF_2)] | Diclofenac | J 1130 |
|  | Bromfenac, Choline and Magnesium salicylate, Methenamine and sodium salicylate , Fenoprofen, Flurbiprofen, Ketoprofen, Naproxen, Oxaprozin, Sulindac, Piroxicam, Etodolac, Meloxicam, Nabumetone, Celecoxib/Celebrex, Indomethacin, Mefenamic acid, Meclofenamate, Diflunisal, Tolmetin, Salsalate, | N/A |
|  | Aspirin | G8598, G8895, G9277, G9793 |
|  | Ibuprofen | J1741 |
|  | Ketorolac | J1885, C9447 |
|  | Diclofenac | J 1130 |
| Selective estrogen receptor modulators (SERMs) [[1](#_ENREF_1), [2](#_ENREF_2)] | Tamoxifen, raloxifene, arzoxifene, bazedoxifene, lasofoxifene, ospemifene, clomifene, cyclofenil, ormeloxifene, toremifene, raloxifene, | N/A |
|  | Tamoxifen | S0187 |
| Statins[[1](#_ENREF_1), [2](#_ENREF_2)] | Atorvastatin, Fluvastatin, Lovastatin, Pitavastatin, Pravastatin, Rosuvastatin, Simvastatin. | N/A |

*NDC codes will be used wherever applicable. ACE: angiotensin-converting enzyme; ARBs: angiotensin II receptor blockers; HCPCS: Healthcare Common Procedure Coding System; NDC: National Drug Code; NSAID: nonsteroidal anti-inflammatory drug.

Table I. List of major bleeding, clinically relevant non-major bleeding, and recurrent VTE codes

| **Category** | **ICD-10 Codes** |
| --- | --- |
| **Major bleeding codes** | |
| Gastrointestinal major bleed (inpatient, first-listed diagnosis position) [[1](#_ENREF_1), [2](#_ENREF_2), [5](#_ENREF_5)] | **ICD-10-CM Codes:** K921, K922, K5731, K625, K31811, K920, K661, K2971, K2211, K5521, K264, K254, K2981, K226, K6381, K3182, K2901, K274, K5711, K5793, K51911, K5791, K282, K276, K272, K266, K262, K256, K252, K250, K5781, K260, K270, K5751, K280, K5741, K284, I8511, K5733, K5721, K5713, K51811, K51411, K51211, K50911, K51011, K51311, K50811, K51511, K50111, K5701, I8501, K2991, K5753, K2961, K2951, K2941, K2931, K2921, K286  **ICD-10-PCS Codes**: no codes |
| Intracranial major bleed (inpatient, first-listed diagnosis position) [[1](#_ENREF_1), [2](#_ENREF_2), [5](#_ENREF_5)] | S065X0A, I615, S066X0A, I6201, I618, I610, I609, S065X9A, I629, I619, I614, I611, S066X9A, I6200, I6202, I6203, I608, S06350A, S065X7A, S066X1A, I613, S065X1A, S06360A, S064X0A, S065X8A, S066X8A, S06358A, S064X9A, S066X3A, I6011, I606, S066X7A, S066X4A, S066X2A, I6052, S066X5A, I6002, I6051, S065X6A, S065X5A, I6010, I607, S065X3A, S065X2A, I6012, S064X7A, S064X5A, S064X3A, I604, S064X1A, S06369A, S06367A, S06366A, S06365A, S06363A, S06362A, S06361A, S06359A, S06357A, S06355A, I6000, I6031, S06354A, S06353A, S06351A, S06349A, S06352A, S06348A, S06356A, S06347A, I6030, S06346A, S06345A, I6032, S06364A, I621, S06368A, S06344A, S06343A, S06342A, S06341A, I6001, S064X2A, S064X4A, S064X6A, I6050, S064X8A, I6022, I616, S06340A, S065X4A, I6021, S066X6A, I6020, I612 |
| Other major bleed (inpatient, first-listed diagnosis position) [[1](#_ENREF_1), [2](#_ENREF_2), [5](#_ENREF_5)] | **ICD-10 CM Codes:** D62, D7801, D7802, D7821, D7822, E3601, E3602, E89810, E89811, G9731, G9732, G9751, G9752, H05231, H05232, H05233, H05239, H1130, H1131, H1132, H1133, H2100, H2101, H2102, H2103, H31301, H31302, H31303, H31309, H31311, H31312, H31313, H31319, H31411, H31412, H31413, H31419, H3560, H3561, H3562, H3563, H35731, H35732, H35733, H35739, H4310, H4311, H4312, H4313, H44811, H44812, H44813, H44819, H47021, H47022, H47023, H47029, H59111, H59112, H59113, H59119, H59121, H59122, H59123, H59129, H59311, H59312, H59313, H59319, H59321, H59322, H59323, H59329, H9521, H9522, H9541, H9542, I312, I97410, I97411, I97418, I9742, I97610, I97611, I97618, I97620, J9561, J9562, J95830, J95831, L7601, L7602, L7621, L7622, M2500, M25011, M25012, M25019, M25021, M25022, M25029, M25031, M25032, M25039, M25041, M25042, M25049, M25051, M25052, M25059, M25061, M25062, M25069, M25071, M25072, M25073, M25074, M25075, M25076, M2508, M96810, M96811, M96830, M96831, N421, N857, N897, N920, N923, N930, N938, N939, N9961, N9962, N99820, N99821, R040, R041, R042, R0489, R049, R233, R310, R319, R58, T792XXA  **ICD-10 PCS Codes:** 30230N1, 30230P1, 30233N1, 30233P1, 30240N1, 30240P1, 30243N1, 30243P1, 30250N1, 30250P1, 30253N1, 30253P1, 30260N1, 30260P1, 30263N1, 30263P1 |
| **Clinically relevant non-major bleeding (CRNMB)** | |
| Gastrointestinal (non-critical care site codes) [[1](#_ENREF_1), [2](#_ENREF_2)] | I8501, I8511, K228, K250, K250, K252, K252, K254, K254, K256, K256, K260, K260, K262, K262, K264, K264, K266, K266, K270, K270, K272, K272, K274, K274, K276, K276, K280, K280, K282, K282, K284, K284, K286, K286, K2901, K2931, K2941, K2951, K2961, K2921, K2931, K2961, K2971, K2991, K2981, K31811, K5711, K5713, K5731, K5791, K5733, K5793, K661, K625, K5521, K920, K921, K922 |
| Gastrointestinal (likely critical care site codes) [[1](#_ENREF_1), [2](#_ENREF_2)] | K661 |
| Intracranial hemorrhage (critical care site codes) [[1](#_ENREF_1), [2](#_ENREF_2)] | I6000, I6001, I6002, I6010, I6011, I6012, I602, I6030, I6031, I6032, I604, I6050, I6051, I6052, I606, I607, I608, I609, I610, I611, I612, I613, I614, I615, I616, I618, I619, I621, I6200, I6201, I6202, I6203, I629, S066X0A, S066X0A, S066X1A, S066X2A, S066X3A, S066X4A, S066X5A, S066X6A, S066X7A, S066X8A, S066X9A, S066X0A, S066X9A, S065X0A, S065X0A, S065X1A, S065X2A, S065X3A, S065X4A, S065X5A, S065X6A, S065X7A, S065X8A, S065X9A, S065X0A, S065X9A, S064X0A, S064X0A, S064X1A, S064X2A, S064X3A, S064X4A, S064X5A, S064X6A, S064X7A, S064X8A, S064X9A, S064X0A, S064X9A, S06360A, S06340A, S06350A, S06360A, S06341A, S06342A, S06351A, S06352A, S06361A, S06362A, S06343A, S06344A, S06353A, S06354A, S06363A, S06364A, S06345A, S06355A, S06365A, S06346A, S06347A, S06348A, S06356A, S06357A, S06358A, S06366A, S06367A, S06368A, S06349A, S06359A, S06369A, S06360A, S06369A |
| Other bleed (non-critical care site codes) [[1](#_ENREF_1), [2](#_ENREF_2)] | H1130, H1131, H1132, H1133, H0289, N3289, R319, R310, R311, R312, N421, N831, N857, N920, N923, N930, N897, N925, N938, N926, N939, R233, R040, R041, R042, R049, R0481, R0489, T792XXA, H9521, H9522, H9541, H9542, J9562, J95830, J95831, K9162, K91840, K91841, L7601, L7602, L7621, L7622, K9161, J9561, I97810, I97811, I97820, I97821, M96810, M96811, M96830, M96831, N9961, N9962, N99820, N99821 |
| Other bleed (critical care site codes) [[1](#_ENREF_1), [2](#_ENREF_2)] | H44811, H44812, H44813, H44819, H35731, H35732, H35733, H35739, H3560, H3561, H3562, H3563, H31301, H31302, H31303, H31309, H31311, H31312, H31313, H31319, H31411, H31412, H31413, H31419, H2100, H2101, H2102, H2103, H05231, H05232, H05233, H05239, H47021, H47022, H47023, H47029, H4310, H4311, H4312, H4313, I312, M2500, M25011, M25012, M25019, M25021, M25022, M25029, M25031, M25032, M25039, M25041, M25042, M25049, M25051, M25052, M25059, M25061, M25062, M25069, M25071, M25072, M25073, M25074, M25075, M25076, M2508, M2500, H59111, H59112, H59113, H59119, H59121, H59122, H59123, H59129, H59311, H59312, H59313, H59319, H59321, H59322, H59323, H59329, I97410, I97411, I97418, I9742, I97610, I97611, I97618, I9762, D7801, D7802, D7821, D7822, G9731, G9732, G9731, G9732, G9751, G9752, E3601, E3602 |
| **Recurrent venous thromboembolism (VTE)** | |
| Recurrent VTE[[1](#_ENREF_1), [3](#_ENREF_3), [6-8](#_ENREF_6)] (inpatient, first-listed diagnosis code) | I26.0*, I26.9*, I80.1*, I80.20*, I82.210, I80.22*, I80.23*, I80.29*, I82.40*, I82.41*, I82.42*, I82.43*, I82.44*, I82.49*, I82.4Y*, I82.4Z*, I82.60*, I82.62*, I82.890, I82.A1*, I82.B1*, I82.C1* |

CRNMB: clinically relevant non-major bleeding; ICD-10-CM: International Classification of Diseases, 10th Revision, Clinical Modification; ICD-10-PCS: International Classification of Diseases, 10th Revision, Procedure Coding System; MB: major bleeding; VTE: venous thromboembolism.

Table J. List of oral and parenteral anticoagulation codes

| **Drug [**[**1**](#_ENREF_1)**,** [**2**](#_ENREF_2)**]** | **Anticoagulant Type** | **Codes*[**[**1**](#_ENREF_1)**,** [**2**](#_ENREF_2)**]** |
| --- | --- | --- |
| Low Molecular Weight Heparin | PAC | - |
| Dalteparin | PAC | **HCPCS code:** J1645 |
| Enoxaparin | PAC | **HCPCS code**: J1650 |
| Tinzaparin | PAC | **HCPCS code**: J1655 |
| Heparin | PAC | **HCPCS code**: J1642, J1644 |
| Fondaparinux | PAC | **HCPCS code**: J1652 |
| Warfarin | OAC | - |
| Apixaban | OAC | **NDC codes:** 00003-0894-31, 00003-0893-91, 00003-0893-31, 00003-0893-21, 00003-0894-91, 00003-0894-70, 00003-0894-21, 55154-0613-00, 55154-0613-08, 00003-3764-74, 00003-3764-42, 00003-3764-32 |
| Dabigatran etexilate mesylate | OAC | - |
| Rivaroxaban | OAC | **NDC codes:** 55154-1422-00, 55154-1424-00, 55154-1424-08, 55154-1423-08, 50458-0580-10, 50458-0578-01, 50458-0578-90, 50458-0578-10, 50458-0578-30, 50458-0579-01, 50458-0579-89, 50458-0579-10, 50458-0579-90, 50458-0579-30, 50458-0577-01, 50458-0577-10, 50458-0577-60, 50458-0577-18, 50458-0575-01, 50458-0580-01, 50458-0580-90, 50458-0580-30 |
| Edoxaban | OAC | - |

*Codes from the NDC system will also be used to characterize the use of PAC. HCPCS: Healthcare Common Procedure Coding System; NDC: National Drug Code; OAC: oral anticoagulant; PAC: parenteral anticoagulation.

## References

1. Cohen A, Keshishian A, Lee T, Wygant G, Rosenblatt L, Hlavacek P, et al. Effectiveness and safety of apixaban, low-molecular-weight heparin, and warfarin among venous thromboembolism patients with active cancer: a US claims data analysis. Thrombosis and Haemostasis. 2021;121(03):383-95.

2. Cichhetti ML, Xuemei. Evaluation of Anticoagulants among Venous Thromboembolism Patients with Active Cancer: Pooled Analysis from Claims Databases (EU PAS number: EUPAS25308): European Medicines Agency (EMA); 2018 [updated 02/04/2024; accessed 2025-04-21]. Available from: <https://catalogues.ema.europa.eu/node/3382/administrative-details>.

3. Lutsey PL, Zakai NA, MacLehose RF, Norby FL, Walker RF, Roetker NS, et al. Risk of hospitalised bleeding in comparisons of oral anticoagulant options for the primary treatment of venous thromboembolism. British journal of haematology. 2019;185(5):903-11.

4. Chronic Conditions Data Warehouse (CCW). 30 CCW Chronic Conditions Algorithms 2024 [accessed 2025-03-03]. Available from: <https://www2.ccwdata.org/documents/10280/19139421/chr-chronic-condition-algorithms.pdf>.

5. Shehab N, Ziemba R, Campbell KN, Geller AI, Moro RN, Gage BF, et al. Assessment of ICD‐10‐CM code assignment validity for case finding of outpatient anticoagulant‐related bleeding among Medicare beneficiaries. Pharmacoepidemiology and drug safety. 2019;28(7):951-64.

6. Fang MC, Fan D, Sung SH, Witt DM, Schmelzer JR, Steinhubl SR, et al. Validity of using inpatient and outpatient administrative codes to identify acute venous thromboembolism: the CVRN VTE study. Medical care. 2017;55(12):e137-e43.

7. Tamariz L, Harkins T, Nair V. Mini-sentinel systematic evaluation of health outcome of interest definitions for studies using administrative data venous thromboembolism report. Mini-Sentinel website August. 2011;11.

8. Dawwas GK, Dietrich E, Smith SM, Davis K, Park H. Comparative effectiveness and safety of direct-acting oral anticoagulants and warfarin in patients with venous thromboembolism and active cancer: an observational analysis. Clinical Therapeutics. 2020;42(9):e161-e76.
